# Unraveling the habitat preferences of two closely related bumble bee species in Eastern Europe

**DOI:** 10.1002/ece3.6232

**Published:** 2020-04-15

**Authors:** Julia C. Geue, Henri A. Thomassen

**Affiliations:** ^1^ Comparative Zoology Institute for Evolution and Ecology University of Tübingen Tübingen Germany

**Keywords:** *Bombus lucorum*, *Bombus terrestris*, Eastern Europe, pollinators, random forests, relative abundance, species distribution

## Abstract

Co‐occurrence of closely related species is often explained through resource partitioning, where key morphological or life‐history traits evolve under strong divergent selection. In bumble bees (genus *Bombus*), differences in tongue lengths, nest sites, and several life‐history traits are the principal factors in resource partitioning. However, the buff‐tailed and white‐tailed bumble bee (*Bombus terrestris* and *B. lucorum* respectively) are very similar in morphology and life history, but their ranges nevertheless partly overlap, raising the question how they are ecologically divergent. What little is known about the environmental factors determining their distributions stems from studies in Central and Western Europe, but even less information is available about their distributions in Eastern Europe, where different subspecies occur. Here, we aimed to disentangle the broad habitat requirements and associated distributions of these species in Romania and Bulgaria. First, we genetically identified sampled individuals from many sites across the study area. We then not only computed species distributions based on presence‐only data, but also expanded on these models using relative abundance data. We found that *B. terrestris* is a more generalist species than previously thought, but that *B. lucorum* is restricted to forested areas with colder and wetter climates, which in our study area are primarily found at higher elevations. Both vegetation parameters such as annual mean Leaf Area Index and canopy height, as well as climatic conditions, were important in explaining their distributions. Although our models based on presence‐only data suggest a large overlap in their respective distributions, results on their relative abundance suggest that the two species replace one another across an environmental gradient correlated to elevation. The inclusion of abundance enhances our understanding of the distribution of these species, supporting the emerging recognition of the importance of abundance data in species distribution modeling.

## Graphical abstract

We used genetic barcoding and species distribution models to disentangle the habitat requirements of two closely related bumble bee species in Romania and Bulgaria. We found that *Bombus terrestris* is a more generalist species than previously thought, but that *B. lucorum* is restricted to forested areas with colder and wetter climates. Models based on presence‐only data suggest a large overlap in their respective distributions, but results on their relative abundance suggest that the two species replace one another across an environmental gradient correlated to elevation.

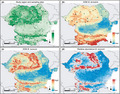

## 1 INTRODUCTION

The co‐occurrence of closely related species has long puzzled evolutionary ecologists. Closely related species are expected to occupy similar niche space through niche conservatism and thus occur in the same regions, but also to compete more strongly with one another than more distantly related species (Anacker & Strauss, 2016). Strong competition may result in the exclusion of the weaker competitor, as well as in rapid divergent evolution of key life‐history traits or phenotypes as a result of resource partitioning (Gause, 1934). In bumble bees (genus *Bombus*), a classical theory is that species evolved a range of different tongue lengths, allowing them to specialize on different floral resources, and to occur sympatrically as a result (Goulson & Darvill, 2004). Other mechanisms of resource partitioning include differences in nest sites, foraging distances, and the spatial use of habitat (Stanley, Knight, & Stout, 2013; Westphal, Steffan‐Dewenter, & Tscharntke, 2006). However, two of the most common bumble bee species in Europe, the buff‐tailed bumble bee (*Bombus terrestris*) and the white‐tailed bumble bee (*Bombus lucorum*), co‐occur (pers. obs.; Goulson, 2010; Goulson, Lye, & Darvill, 2008b; Kells, Holland, & Goulson, 2001; Stanley et al., 2013), despite being very similar in their morphology, choice of nest sites, and life‐history (Goulson, 2010; Stanley et al., 2013). They even possess the same tongue lengths (Goulson, Hanley, Darvill, Ellis, & Knight, 2005; Stanley et al., 2013) and hence occupy a very similar dietary niche space (Goulson et al., 2008b). This begs the question to what extent their ecological niches overlap and—conversely—how they are divergent. Despite many studies into their ecology and behavior (e.g. Bossert, Gereben‐Krenn, Neumayer, Schneller, & Krenn, 2016; Scriven et al., 2015; Stanley et al., 2013; Walther‐Hellwig & Frankl, 2000), and broad‐scale evidence that their ranges only partially overlap (e.g. Rasmont, Franzen, et al., 2015), their distributions at smaller scales remain equivocal. One reason for this ambiguity may be the fact that these species are morphologically highly variable within species, yet very similar between species (Bossert, 2014; Murray, Fitzpatrick, Brown, & Paxton, 2007; Waters, Darvill, Lye, & Goulson, 2011). As a consequence, they may be difficult to distinguish in the field, depending on where they occur and whether queens, males, or workers are compared. In mainland Europe, identification can be complex because both species possess a white abdomen (Gammans, Comont, Morgan, & Perkins, 2018; Rasmont, Coppee, Michez, & Meulemeester, 2013), in contrast to those in Great Britain (Murray et al., 2007). Queens and males can be distinguished (Bertsch, Schweer, & Titze, 2004; Goulson, 2010), but workers (especially of some subspecies of *B. terrestris*) are difficult to discriminate (Williams, 1994). Indeed, in Central Europe, only 45.5% of *B. lucorum* workers could be correctly identified and distinguished from *B. terrestris* workers using the most up‐to‐date morphological key (Wolf, Rohde, & Moritz, 2009). As a consequence, many studies focusing on the ecology or behavior of European bumble bees group these taxonomically well‐recognized species together (Bommarco, Lundin, Smith, & Rundlöf, 2011; Carvell, 2002; Goulson et al., 2005; Meek et al., 2002; Pywell et al., 2005; Walther‐Hellwig & Frankl, 2000), leading to imprecise information on habitat preferences (Murray et al., 2007; Scriven et al., 2015) and other life‐history traits (Stanley et al., 2013). One of the few studies that investigated the ecological preferences of those species separately identified differences in nesting site selection at small spatial scales in Sweden (Svensson, Lagerlöf, & Svensson, 2000) where *B. terrestris* preferred more open habitat, such as fields in agricultural landscapes, and *B. lucorum* preferentially built nests close to forest boundaries. Also in Austria, *B. lucorum* appeared to frequently occur in forested areas (Bossert et al., 2016). At the scale of Europe, *B. lucorum* occurs at higher latitudes than *B. terrestris*, suggesting a differentiation based on temperature (Rasmont, Franzen, et al., 2015). Most studies on these species focused on Western and Central Europe, but little attention has been paid to Eastern Europe, where the situation is complicated by the occurrence of two subspecies of *B. terrestris, B. t. terrestris* and *B. t. dalmatinus,* that are morphologically variable (Lecocq, Rasmont, Harpke, & Schweiger, 2016; Rasmont et al., 2013). Hence, their distribution patterns in Eastern Europe remain equivocal.

Here, we investigate the distributions and broad habitat characteristics of buff‐tailed (*B. terrestris*) and white‐tailed bumble bee (*B. lucorum*) in Bulgaria and Romania, where they are the two most common bumble bee species. *B. lucorum* is one of three cryptic species, the other two being *B. cryptarum* and *B. magnus*. Here, we only include *B. lucorum*, because we did not find any individuals of the latter two species, despite extensive sampling efforts. We hypothesized that the differential habitat use at small scales can be scaled up to landscape scale habitat preferences across a spatial extent of hundreds of kilometers. We first genetically identified the species at sites where multiple individuals were sampled, providing a reliable tool for species identification (Bossert et al., 2016; Murray et al., 2007; Stanley et al., 2013; Waters et al., 2011; Williams et al., 2012; Wolf et al., 2009). We subsequently created species distribution models (SDMs), which have been used previously in quantitative ecological studies of bumble bees (Casey, Rebelo, Rotheray, & Goulson, 2015; Herrera, Ploquin, Rodríguez‐Pérez, & Obeso, 2014; Kadoya, Ishii, Kikuchi, Suda, & Washitani, 2009; Pradervand, Pellissier, Randin, & Guisan, 2014; Rasmont, Franzen, et al., 2015). They are usually based on presence‐only or presence–absence data, with the assumption that the modeled probabilities of occurrence are indicative of abundance (e.g. Dallas & Hastings, 2018). However, recent work across multiple species suggests that these so‐called abundance–suitability relationships are often weak (Dallas & Hastings, 2018; Howard et al., 2014; Mi, Huettmann, Sun, & Guo, 2017). For that reason, the collection and use of abundance data to improve the accuracy of species distribution models was highly recommended (Howard et al., 2014), but still not commonly applied to date. To improve our distribution models, and to specifically investigate their co‐occurrence and the associated abiotic factors driving variation in abundance patterns, we therefore also collected and modeled the relative abundance of these species.

## 2 METHODS

### 2.1 Study species and study area

Bumble bees (*Bombus* sp.) are important pollinators for crops and wild plants, in particular in temperate ecosystems (Corbet, Williams, & Osborne, 1991; Murray et al., 2007). Their body is covered in a dense, colored fur that enables them to be endothermic (Heinrich, 1993), and hence adapt to cold climates, such as alpine and arctic environments. Their distribution extends much further north than that of other bees, and their colonies have been found in the extreme northern regions of the northern hemisphere (Goulson, 2010). *Bombus terrestris* and *Bombus lucorum* are two of the most common bumble bee species in Europe*.* These species have very similar life cycles and are often found in the same areas. Both species use underground nest sites and often choose already existing holes, previously used by small rodents (Goulson, 2010). They possess similar tongue lengths, and as a result forage primarily on the same short corollas and daisy type of flowers (personal observation and (Goulson, Lye, & Darvill, 2008a). In addition, their workers almost perfectly resemble each other, and only the queens and males can be identified reliably (Murray et al., 2007; Wolf et al., 2009), but field identification remains complicated due to the subtlety of morphological differences.

We conducted our study in Bulgaria and Romania, two neighboring countries in southeastern Europe, covering an area of approximately 350.000 km^2^. These countries present a heterogeneous landscape, comprising continental, Mediterranean, and temperate climatic zones, consisting of natural areas such as mountains, river valleys, forests, open woodlands, and grasslands, as well as areas inhabited and influenced by humans, including extensive agricultural lands. The Danube River forms a natural border along much of its length between Romania in the north and Bulgaria in the south**.** Large mountainous areas are present in both countries: the Carpathians in Romania, and the Balkan, Rila, Rhodope, and Pirin mountains in Bulgaria. As a result of this variety of habitats, different biogeographical regions are recognized: the continental, alpine, steppic, Black Sea, and pannonian regions (Council of Europe (COE) (2015)). This high habitat heterogeneity represents an interesting area for evaluating habitat preferences and niche differentiation within and among species.

### 2.2 Sampling

**FIGURE 1:**
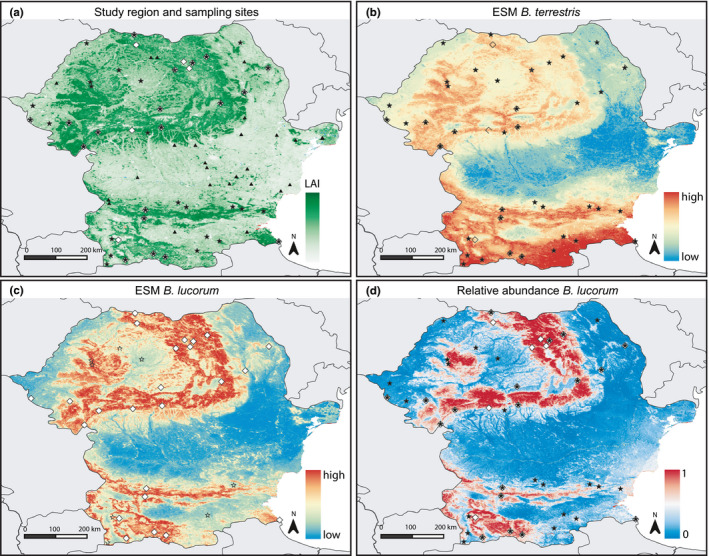
Study region with sampling sites, species distribution modeling results based on ensembles of small models, and relative abundance modeling results. (a) Study area with sampling sites indicated in black stars for *Bombus terrestris* and in white diamonds for *B. lucorum*. Sites where we searched for bumble bees, but did not find any are indicated in black triangles. The background map is annual mean Leaf Area Index (LAI mean), a measure of vegetation greenness. (b) Ensemble of small models for *B. terrestris*. (c) Ensemble of small models for *B. lucorum*. The colors in panel (b) and (c) indicate the probability of occurrence, with warmer colors indicating higher probabilities. Black stars (b) and white diamonds (c) indicate the sampling populations. (d) Machine learning ensemble for the relative abundance of *B. lucorum.* Warmer colors indicate a higher abundance of *B. lucorum* relative to *B. terrestris*

We collected 743 individuals compromising queens and workers of *Bombus terrestris* and *Bombus lucorum* over a timeframe of 4 years (2013, 2014, 2015, and 2017), in each between April and July. We sampled 44 locations in total (Figure 1a and Table 1), which were selected to span a broad range of habitat conditions in both entirely natural and semi‐natural or cultivated environments, as well as along environmental gradients (altitude, vegetation, and climate). We visited additional locations where we searched for, but did not find any bumble bees. These locations were not included as true absences in our species distribution models, but served in computing a sampling bias map (see below). Sampling locations were located at least 20 km apart to rule out the possibility of overlapping foraging ranges (Chapman, Wang, & Bourke, 2003; Westphal et al., 2006) and were visited only once. At each sampling location, capturing efforts were undertaken by 2–3 people for 1.5–2 hrs between 1 hr after sunrise and 1 hr before sunset. Individuals were collected on suitable forage patches with a radius of 100 m, using an entomological net. Individuals were visually identified as one of the two study species, anesthetized in a killing jar with a 1.5 cm layer of plaster of paris saturated with ethyl acetate, and immediately upon cessation of movement stored in 96% ethanol (Smithers, 1988). After fieldwork, specimens were stored frozen at −20°C in the laboratory at the University of Tübingen.

**TABLE 1:** Sampling locations and sample sizes of *Bombus terrestris* and *Bombus lucorum*

	Location	Latitude	Longitude	No. of individuals	
				*Bombus terrestris*	*Bombus lucorum*
1	Baita Plai	46.46871	22.61674	21	0
2	Billed	45.91412	20.94701	9	0
3	Blandesti	47.71380	26.86323	33	0
4	Brebu	45.42815	21.97966	20	1
5	Burja	43.02797	25.32507	5	0
6	Carei	47.69646	22.48073	24	0
7	Cerna	45.15962	22.80671	4	16
8	Coastra	45.14758	24.22260	9	0
9	Corbeni	45.29905	24.60912	5	8
10	Dobrovat	46.99043	27.65404	24	1
11	Drӑgusani	46.29929	26.97973	22	2
12	Föen	45.51085	20.87627	31	1
13	Golitsa	42.90956	27.52514	13	0
14	Gothal	45.40790	21.42069	29	0
15	Grohotno	41.70118	24.38684	10	25
16	Gura Glodului	47.13575	25.50107	2	20
17	Gura Haitii	47.17505	25.25018	0	15
18	Handal	47.65028	23.89441	0	21
19	Hlyabovo	42.06055	26.26459	11	0
20	Iesle	47.31038	25.89774	1	12
21	Iod	46.93652	25.00172	0	12
22	Kamenitsa	41.64449	23.17299	12	0
23	Koevtsi	43.15832	25.09082	21	0
24	Levochevo	41.60707	24.72302	2	28
25	Mengishevo	43.03566	26.64753	13	0
26	Ojdula	45.98988	26.29976	1	15
27	Orsova	44.75420	22.39480	14	2
28	Pastra	42.12283	23.20023	3	0
29	Pietroasa	46.58998	22.58807	12	0
30	Pirin	41.52480	23.58790	4	11
31	Poienita	45.82299	24.57591	19	1
32	Polovragi	45.21492	23.77486	0	12
33	Razdelna	42.18144	25.90854	4	0
34	Rilski Manastir	42.09243	23.38633	0	3
35	Rish	42.97442	26.90731	32	0
36	Sinemorets	42.04499	27.95808	11	1
37	Stambolovo	41.78435	25.63166	15	0
38	Strumeshnitsa	41.39833	23.06046	20	0
39	Topa Mica	46.92851	23.40238	21	0
40	Toplita	46.98115	25.40812	0	2
41	Valea Hotarului	47.93870	23.83761	20	1
42	Valea Pӑdurii	46.62236	24.02727	12	0
43	Zdravets	42.94361	24.15964	6	9
44	Zlatitza	42.70908	24.12053	3	6
	Total			518	225

### 2.3 Species identification

Because of the previously described difficulties in distinguishing species based on external morphology, we used a 1,043 bp long fragment of the cytochrome c oxidase subunit I (COI) gene, which is known for its relatively fast mutation rate, and is used across many taxa for genetic identification purposes, including bumble bees (Bertsch et al., 2004; Bossert et al., 2016; Hebert, Stoeckle, Zemlak, & Francis, 2004; Murray et al., 2007; Waugh, 2007; Williams et al., 2012). This long fragment is completely overlapping with an ~890 bp region used by Bertsch et al. (2004) to distinguish between the closely related *Bombus cryptarum, B. magnus,* and *B. lucorum* and was therefore used here to distinguish between *B. lucorum* and *B. terrestris*. DNA was extracted from one or both middle legs using the QIAmp DNA Micro Kit (Qiagen) following the manufacturer's protocol. PCR was performed with primers originally developed for *Apis* (Tanaka, Roubik, Kato, Liew, & Gunsalam, 2001): forward 5'‐ATAATTTTTTTTATAGTTATA‐3' and reverse 5'‐GATATTAATCCTAAAAAATGTTGAGG‐3'. They were used to amplify a 1,043 bp long fragment of the COI gene. The PCR reaction mix consisted of 2.5 μl of 10× PCR Buffer S (Genaxxon), 15.4 μl HPLC water, 1.0 μl dNTP's, 1.0 μl MgCl_2_ (25 mM), 1.0 μl BSA, 1.0 μl of each primer (0.1 mM), 0.1 μl Taq polymerase, and 2 μl extracted DNA. PCRs were performed with a Mastercycler epgradient (Eppendorf) with the following conditions: an initial denaturation step at 95°C for 1 min, followed by 55 cycles of a 3‐step process: denaturation for 40 s at 95°C, annealing for 1 min at 45°C, and extension for 2 min at 60°C with a final extension step at 60°C for 4 min. PCR products were visualized using agarose gel electrophoresis to check for the amplification of the fragment. Successfully amplified PCR products were cleaned up using the Promega Wizard SV Gel and PCR Clean‐Up System according to the manufacturer's protocol. Cleaned up samples were then sent to LGC Genomics for sequencing with the reverse primer only. Sequences were visualized and edited with Geneious R8 (Biomatters, Ltd.; Kearse et al., 2012). None of the obtained sequences showed any signs of cross‐contamination (e.g., double peaks in the chromatograms or ambivalent species identification). We used two methods to assign a species to the sequenced samples. First, we blasted sequences in GenBank (NIH genetic sequences database) and assigned the species with the highest identity (range ~95%–100%) to the corresponding sample (Table S1). In addition, we created a phylogenetic tree (Figure S1), which included reference sequences for various *Bombus* species obtained from Genbank (Table 2). We included reference sequences from various geographic origins, because we expected that genetic intraspecific variability should be smaller than interspecific differences, and thus that a well‐supported clustering of our samples with the reference sequences suggests high confidence in the species identification. To construct the phylogenetic tree, we first identified the most likely substitution model in MEGA‐X (Kumar, Stecher, Li, Knyaz, & Tamura, 2018). We then created a Bayesian phylogenetic tree in Geneious R8 with the MrBayes module (Huelsenbeck & Ronquist, 2001), using one cold and four heated chains with a temperature of 0.2 and a chain length of 1.1 million and a burn‐in of 100,000, five gamma categories, a sampling frequency of 200, unconstrained branch lengths (GammaDir [1,0.1,1,1]), and exponential shape distribution. We used a reference sequence of *B. pascuorum* as an outgroup (Table 2). Individuals that were included in a monophyletic group with reference sequences of *B. terrestris* or *B. lucorum* were considered members of the corresponding target species. Comparisons to the blast results revealed no differences, and all individuals of *B. terrestris* and *B. lucorum* were included in subsequent species distribution models.

**TABLE 2:** Reference COI sequences obtained from GenBank and used for the phylogenetic tree

Species	GenBank accession no.	Author
*Bombus terrestris*	AY181171	Pedersen (2002)
	AY181170	Pedersen (2002)
	AY181169	Pedersen (2002)
	KT164618	Tang et al. (2015)
*Bombus lucorum*	AY181121	Pedersen (2002)
	AY181119	Pedersen (2002)
	AY181117	Pedersen (2002)
	KT164681	Tang et al. (2015)
*Bombus sporadicus*	AF279500	Tanaka, Ito, & Inoue, (2000)
	AY181163	Pedersen, (2002)
	MF409659	Potapov et al. (2017)
*Bombus cryptarum*	AY181100	Pedersen (2002)
	AY530011	Bertsch et al. (2004)
	AF279485	Tanaka et al. (2000)
*Bombus magnus*	AY181123	Pedersen (2002)
	AY530014	Bertsch et al. (2004)
	KC192046	Vesterlund, Sorvari, & Vasemägi, (2014)
*Bombus hortorum*	AY181105	Pedersen (2002)
*Bombus pratorum*	AY181145	Pedersen (2002)
*Bombus pascuorum*	AY181141	Pedersen (2002)

### 2.4 Environmental variables

In order to create species distribution models, we used a set of environmental variables at 30 arcsec resolution (Table 3). We initially considered 19 climate variables from the WorldClim database (http://www.worldclim.org), which included temperature and precipitation variables based on a 30‐year climatology from 1970 to 2000 (Fick & Hijmans, 2017). Additionally, elevation data were acquired from the Shuttle Radar Topography Mission (SRTM; https://www2.jpl.nasa.gov/srtm/), and used directly, as well as to compute aspect and slope. Because distribution patterns and habitat preferences of bumble bees have previously been suggested to relate to vegetation characteristics and forest cover, we also included spatial and temporal vegetation patterns derived from satellite data. We used percent tree cover and Leaf Area Index (LAI, the one‐sided green leaf area per unit ground area), which were both obtained from the Global Land Cover Facility database (http://www.glcf.umd.edu/data/). Information on the vertical forest structure, that is, canopy height, was derived from spaceborne LiDAR from 2011 (Simard, Pinto, Fisher, & Baccini, 2011). Canopy height was found to be a better predictor for species distributions than other remote sensing variables such as canopy cover or land‐use variables (Ficetola, Bonardi, Mücher, Gilissen, & Padoa‐Schioppa, 2014; Goetz, Steinberg, Dubayah, & Blair, 2007), and we hypothesized that it may be related to forest understory flower availability and the presence and abundance of flowering tree species relevant for these bumble bee species. Finally, to include information about surface moisture, we included annual mean, minimum, maximum, and seasonality, computed from raw Quickscat data (Geue, Vágási, Schweizer, Pap, & Thomassen, 2016). To do so, we used daily raw backscatter measurements downloaded from the BYU Scatterometer Climate Record Pathfinder database (http://www.scp.byu.edu/data/Quikscat/SIRv2/qush/Eur.html) over the period the instrument was online (2000–2008). We excluded highly correlated variables, which we identified by means of their variance inflation factor (VIF). To do so, we used the automated stepwise exclusion procedure implemented in the “usdm” package v. 1.1‐18 in R 3.6.1 (R Development Core Team, 2008), keeping only those variables with VIF < 10. The final data set consisted of 16 variables (Table 3).

**TABLE 3:** Environmental variables used for species distribution modeling and random forest analyses

Variable	Meaning	Source
Bio 1	Annual mean temperature	http://www.worldclim.org
Bio 2	Mean diurnal range [mean of monthly (max temp – min temp)]	http://www.worldclim.org
**Bio 3**	Isothermality [(Bio2/Bio7) * 100]	http://www.worldclim.org
**Bio 4**	Temperature seasonality [standard deviation * 100]	http://www.worldclim.org
Bio 5	Max temperature of warmest month	http://www.worldclim.org
Bio 6	Minimum temperature of coldest month	http://www.worldclim.org
Bio 7	Temperature annual range (Bio5‐Bio6)	http://www.worldclim.org
**Bio 8**	Mean temperature of wettest quarter	http://www.worldclim.org
**Bio 9**	Mean temperature of driest quarter	http://www.worldclim.org
Bio 10	Mean temperature of warmest quarter	http://www.worldclim.org
**Bio 11**	Mean temperature of coldest quarter	http://www.worldclim.org
Bio 12	Annual precipitation	http://www.worldclim.org
Bio 13	Precipitation of wettest month	http://www.worldclim.org
**Bio 14**	Precipitation of driest month	http://www.worldclim.org
**Bio 15**	Precipitation seasonality (coefficient of variation)	http://www.worldclim.org
Bio 16	Precipitation of wettest quarter	http://www.worldclim.org
Bio 17	Precipitation of driest quarter	http://www.worldclim.org
Bio 18	Precipitation of warmest quarter	http://www.worldclim.org
**Bio 19**	Precipitation of coldest quarter	http://www.worldclim.org
Elevation	Elevation	https://www2.jpl.nasa.gov/srtm/
**Aspect**	Aspect	https://www2.jpl.nasa.gov/srtm/
**Slope**	Slope	https://www2.jpl.nasa.gov/srtm/
**LAI sd**	Leaf Area Index standard deviation across the year	http://www.glcf.umd.edu/data/
LAI min	Leaf Area Index annual minimum	http://www.glcf.umd.edu/data/
**LAI mean**	Leaf Area Index annual mean	http://www.glcf.umd.edu/data/
LAI max	Leaf Area Index annual maximum	http://www.glcf.umd.edu/data/
**Tree**	Percent tree cover	http://www.glcf.umd.edu/data/
**Canopy**	Canopy height	Simard et al. (2011)
**QSCAT mean**	Surface moisture (mean)	http://www.scp.byu.edu, Geue et al. (2016)
QSCAT min	Surface moisture (min)	http://www.scp.byu.edu, Geue et al. (2016)
QSCAT max	Surface moisture (max)	http://www.scp.byu.edu, Geue et al. (2016)
**QSCAT season**	Surface moisture (coefficient of variation)	http://www.scp.byu.edu, Geue et al. (2016)

Note

Variables marked in bold were selected for our models after stepwise removal of variables with a variance inflation factor > 10.

### 2.5 Species distribution modeling

#### 2.5.1 Spatial autocorrelation and sampling bias

Spatial autocorrelation is a major statistical challenge in spatial analyses, causing inflated measures of predictive power and incorrect distribution models (e.g. Guélat, Kéry, & Isaac, 2018; Segurado, Araujo, & Kunin, 2006). There are two main causes for spatial autocorrelation in species distribution modeling.

First, there is often a spatial clustering of sampling sites. Reasons for such clustering are manifold and may be related to the sampling design (for instance ease of access, or issues with the logistics of evenly spaced sampling), or to the biotic and abiotic drivers of species distributions themselves, such as gaps in a species' range due to unsuitable habitat. Many approaches have been proposed to correct for sampling bias, among which subsampling locations to acquire a more even distribution of known presences are optimal in most cases (Fourcade, Engler, Rodder, & Secondi, 2014). As a first step, we therefore removed one of the sites of pairs that were located within 20 km from one another. However, because in our study the number of locations is rather limited, further subsampling would result in an even smaller data set. Hence, in a second step, we instead weighted each location based on the density of known presences within a given radius, which was shown to be a good alternative to subsampling as a correction method (Fourcade et al., 2014; Stolar, Nielsen, & Franklin, 2015). To do so, we created a bias grid in QGIS 3.4.4. (Development Team QGIS, 2017) at 30 arcsec resolution, with each grid cell representing the density of sampling locations within a 50 km radius, and kernel densities following a Gaussian distribution (Balestrieri et al., 2016). We used the inverse of the density to weight each presence and background location (see below), thus downweighting clustered locations. We not only included locations of known presence in this bias grid, but also locations where we searched for bumble bees with similar effort, but did not find any. We restricted these putative absence locations to those that were at least 50 km apart from known presences. We specifically only included these sites in our sampling bias map, and not in our models, because we cannot be sure that these represent true absences.

The second cause of spatial autocorrelation in SDMs is the often inherent spatial autocorrelation of habitat conditions, in particular climate variables. In this case, species occurrences are spatially dependent on the underlying habitat variables and thus represent a true association between species presence and local conditions. It is often impossible and undesirable to a priori remove spatial autocorrelation due to spatial dependence. Instead, spatial autocorrelation is expected to be absent in model residuals, regardless of the presence of initial spatial dependence. Models should correctly predict the presence or absence of a species at any given location, independent of its spatial relation to other locations. We thus tested for spatial autocorrelation in the probabilities of occurrence at known presence locations using global Moran's I in the R package “lctools” v.0.2‐7. We used four neighbors and tested the significance of correlations with resampling and randomization procedures.

#### 2.5.2 Presence‐only data

To model species distributions based on presence‐only data, we used an ensemble method, which has been shown to perform better than any given individual modeling method (e.g. Araújo & New, 2007; Elith & Graham, 2009; Marmion, Luoto, Heikkinen, & Thuiller, 2009). Because the number of known locations of species presence was limited, we employed the ensemble of small models approach implemented in the “ecospat” R package (Breiner, Guisan, Bergamini, Nobis, & Anderson, 2015; Breiner, Nobis, Bergamini, Guisan, & Isaac, 2018; Di Cola et al., 2017). Ecospat fits bivariate models of presence/(pseudo‐)absence with two predictor variables at a time, creating an ensemble of “small” models weighted by each bivariate model's performance. It can do so for multiple modeling approaches, using the “Biomod2” package for R (Thuiller, Lafourcade, Engler, & Araújo, 2009). Hence, for each modeling approach, bivariate (small) models are combined into a model ensemble, and model ensembles are in turn combined into an overall ensemble. We used ecospat v.3.1 and Biomod2 v.3.3‐19 to run Maxent models (specifically the MAXENT.Phillips models, as implemented by Phillips, Anderson, & Schapire, 2006), generalized linear models (GLM), classification tree analysis (CTA, also known as classification and regression trees (CART); Breiman, Friedman, Olshen, & Stone, 1984), and artificial neural networks (ANN; Ripley, 1996). In a recent study comparing 10 different modeling approaches implemented in ecospat and Biomod2, these were shown to be the top performing ones, while keeping computation times manageable (Breiner et al., 2018). We used model tuning to optimize the parameter settings for each model.

We generated input files using presence‐only sites and 5,000 background points that were sampled randomly at a minimum distance of 20 km from known presences. To correct for sampling bias, we extracted weights for all locations, which were implemented using the Yweights argument in ecospat.

To evaluate model performance, we computed various evaluation scores and used K‐fold cross‐validation with subsets of training and testing data. The Boyce index (Hirzel, Lay, Helfer, Randin, & Guisan, 2006) is specifically designed and hence a particularly appropriate evaluation score for presence‐only models. It is limited between −1 and 1, with 0 indicating model performance no better than random, and values close to 1 indicating high performance. We used the Boyce index to assess model performance, but also report the area under the receiver operator curve (AUC; Swets, 1988), Cohen's kappa (Cohen, 1960; Hirzel et al., 2006), and the true skill statistic (TSS; Allouche, Tsoar, & Kadmon, 2006). To create training and testing data partitions for K‐fold cross‐validation, we used spatial blocking. Partitioning the data into spatial blocks has the advantage over random allocation of sites that it is better suited to evaluate model performance in the potential presence of spatial autocorrelation (e.g. Roberts et al., 2017). If a model performs well, it is expected to correctly predict occurrences in both distant as well as nearby locations (Telford & Birks, 2009). We generated spatial blocks of training and testing data with the R package “blockCV” v.2.0.0. (Valavi, Elith, Lahoz‐Monfort, Guillera‐Arroita, & Warton, 2018). We created fivefold and set the size of the spatial blocks to the median of the spatial autocorrelation range across the input environmental variables, which were sampled at 5,000 random locations. To find evenly dispersed folds, we ran 100 iterations.

Finally, to visually inspect species occurrence as a function of environmental conditions, we created two types of response curves. In the first, we plotted the response as a function of one environmental variable, while letting all other variables covary. These curves are particularly useful to understand the spatial patterns of species distributions. The curves cover the full range of responses, where the model takes advantage of sets of variables changing together. Second, we also plotted marginal response curves, where we plotted the effect of one environmental variable, while keeping all other variables at their sampled median. These curves are informative with respect to the individual contributions of each environmental variable.

#### 2.5.3 Relative abundance data

To test whether the relative abundance of *B. lucorum* compared to *B. terrestris* is associated with environmental conditions, we used a machine learning approach implemented in the “SuperLearner” (v.2.0‐25) package for the R statistical framework. SuperLearner uses model tuning to optimize model parameter settings and cross‐validation to estimate the performance of multiple models. It creates optimized ensembles, weighted by the performance of the individual models. Where possible, we ran models similar to those for the presence‐only data: generalized additive models (GAM; Hastie & Tibshirani, 1986), generalized linear models (GLM), Bayesian additive regression trees (BART; Chipman, George, & McCulloch, 2010), random forests (RF; Breiman, 1996; Breiman, 2001; Breiman et al., 1984), and neural networks (ANN; Ripley, 1996). We also ran a very simple mean‐of‐abundance model as a baseline. We corrected for sampling bias using the weighting method described above, but we also fitted models to uncorrected abundance. We ran models on the full data set, where bagging and randomization were done internally, as well as on a partial data set, where we omitted 20% of the data, which were used as test data for independent cross‐validation. For each model, we report its associated risk (a measure of model performance) and coefficient (the weight with which it is included in the ensemble). Response curves were created as described above.

To subsequently create a map of the predicted relative abundance of *B. lucorum* across the entire study area, we extracted the values for environmental variables at all 30 arcsec gridcells within Bulgaria and Romania. We then used the “predict.SuperLearner” function to project the known relationship between relative abundance and environmental conditions onto the entire landscape. These values were imported and converted to a raster format in QGIS 3.4.4 (Development Team QGIS, 2017).

## 3 RESULTS

### 3.1 Species identification

The most likely substitution model was the General Time Reversible (GTR) model with gamma distribution, which we implemented to create the phylogenetic tree. We found that 514 individuals clustered with reference sequences of *B. terrestris* and 220 with those of *B. lucorum* (Table 1)*.*

### 3.2 Presence‐only models

Boyce indices for individual K‐fold cross‐validated models for *B. terrestris* ranged between 0.434 and 0.878 (median 0.751), suggesting overall decent to good model performance, except for those based on classification trees (CTA; Table 4). CTA models were therefore not included in the final ensemble. Boyce indices for ensemble cross‐validated models ranged between 0.133 and 0.869. For *B. lucorum*, Boyce indices for individual cross‐validated models ranged between 0.594 and 0.936 (median 0.766), and for ensembles between 0.650 and 0.870 (Table 4). CTA models performed as poorly as those for *B. terrestris* and were not included in the ensembles. Overall, models for *B. lucorum* performed slightly better than those for *B. terrestris*.

**TABLE 4:** Performance scores of ESMs using presence‐only data

Model	*Bombus terrestris*				*Bombus lucorum*			
	Boyce	AUC	Kappa	TSS	Boyce	AUC	Kappa	TSS
RUN1_ANN	0.751	0.696	0	0	0.701	0.782	0	0
RUN1_CTA	‐	0.5	0	0	‐	0.5	0	0
RUN1_GLM	0.871	0.708	0	0	0.841	0.754	0	0
RUN1_MAXENT.P	0.553	0.69	0.014	0.36	0.716	0.774	0.027	0.594
RUN1_ENS	0.632	0.69	0.012	0.332	0.725	0.774	0.021	0.561
RUN2_ANN	0.737	0.599	0	0	0.825	0.796	0	0
RUN2_CTA	—	0.5	0	0	—	0.5	0	0
RUN2_GLM	0.694	0.665	0	0	0.936	0.862	0.179	0.327
RUN2_MAXENT.P	0.467	0.65	0.009	0.32	0.837	0.791	0.01	0.654
RUN2_ENS	0.555	0.65	0.027	0.262	0.87	0.796	0.008	0.611
RUN3_ANN	0.814	0.755	0.067	0.149	0.644	0.838	0	0
RUN3_CTA	—	0.5	0	0	—	0.5	0	0
RUN3_GLM	0.833	0.736	0.285	0.167	0.721	0.839	0	0
RUN3_MAXENT.P	0.434	0.71	0.007	0.413	0.766	0.818	0.024	0.638
RUN3_ENS	0.133	0.714	0.006	0.367	0.779	0.82	0.023	0.636
RUN4_ANN	0.764	0.782	0.136	0.27	0.808	0.688	0	0
RUN4_CTA	—	0.5	0	0	—	0.5	0	0
RUN4_GLM	0.636	0.785	0.13	0.355	0.755	0.662	0	0
RUN4_MAXENT.P	0.476	0.724	0.013	0.369	0.844	0.684	0.007	0.515
RUN4_ENS	0.635	0.74	0.015	0.368	0.854	0.685	0.005	0.459
RUN5_ANN	0.852	0.761	0	0	0.819	0.779	0	0
RUN5_CTA	—	0.5	0	0	—	0.5	0	0
RUN5_GLM	0.821	0.779	0.068	0.214	0.721	0.829	0.11	0.334
RUN5_MAXENT.P	0.878	0.768	0.021	0.458	0.594	0.815	0.055	0.677
RUN5_ENS	0.869	0.77	0.017	0.414	0.65	0.816	0.051	0.666

Note

Five cross‐validated models were run based on spatial blocks generated with the R package “blockCV”. MAXENT.P is the MAXENT.Phillips model. ENS is the ensemble of small models.

Spatial autocorrelation in the predicted occurrences was absent for *B. lucorum* (Moran's *I* = 0.08, expected *I* = −0.04, resampling *z* = 1.08, resampling *p* = .280, randomization *z* = 1.09, randomization *p* = .276). However, for *B. terrestris* we still found significant spatial autocorrelation, despite correcting for sampling bias (Moran's *I* = 0.41, expected *I* = −0.03, resampling *z* = 4.44, resampling *p* < .001, randomization *z* = 4.47, and randomization *p* < .001). We visually inspected the predictive map and compared it to maps of important environmental variables. We found that particularly high probability of occurrence was predicted for sites in Mediterranean Bulgaria, which is consistent with the pattern of seasonality in surface moisture (QSCAT season), the most important variable in predicting the species' distribution. We suspected that the remaining spatial autocorrelation was the result of spatial dependence rather than of sampling bias. We further tested for residual spatial autocorrelation in a second analysis, where we also extracted the predictions for sites where we searched for bumble bees, but did not find any, despite making the same sampling effort. These sites were the same as those used to generate a sampling bias grid and were located at least 50 km from known presences. Although these sites were not included in the models as true absences, we expected that a well‐performing model should predict low probability of occurrence for these sites. Indeed, this time we found no evidence for spatial autocorrelation (Moran's *I* = 0.12, expected *I* = −0.02, resampling *z* = 1.67, resampling *p* = .096, randomization *z* = 1.65, and randomization *p* = .099), and we concluded that sampling bias was sufficiently well corrected for.

**FIGURE 2:**
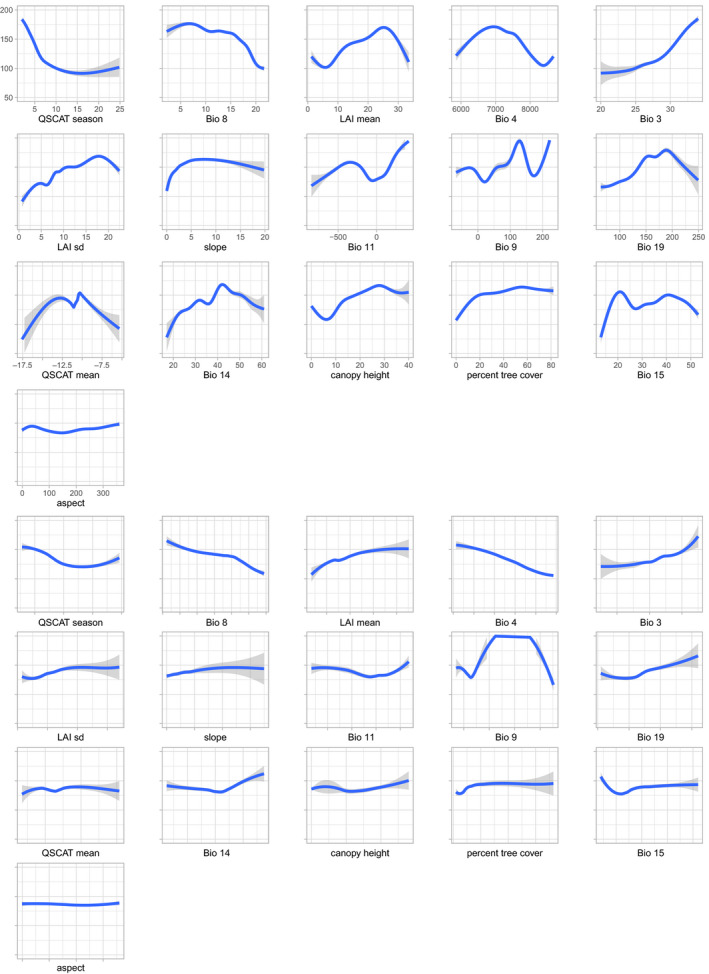
Overall (top panels) and marginal (bottom panels) response curves for presence‐only model predictions for *Bombus terrestris*. Overall response curves were generated for each variable, while letting all other variables covary. In contrast, marginal response curves were created for each variable, while keeping all other variables at their median observed values

**FIGURE 3:**
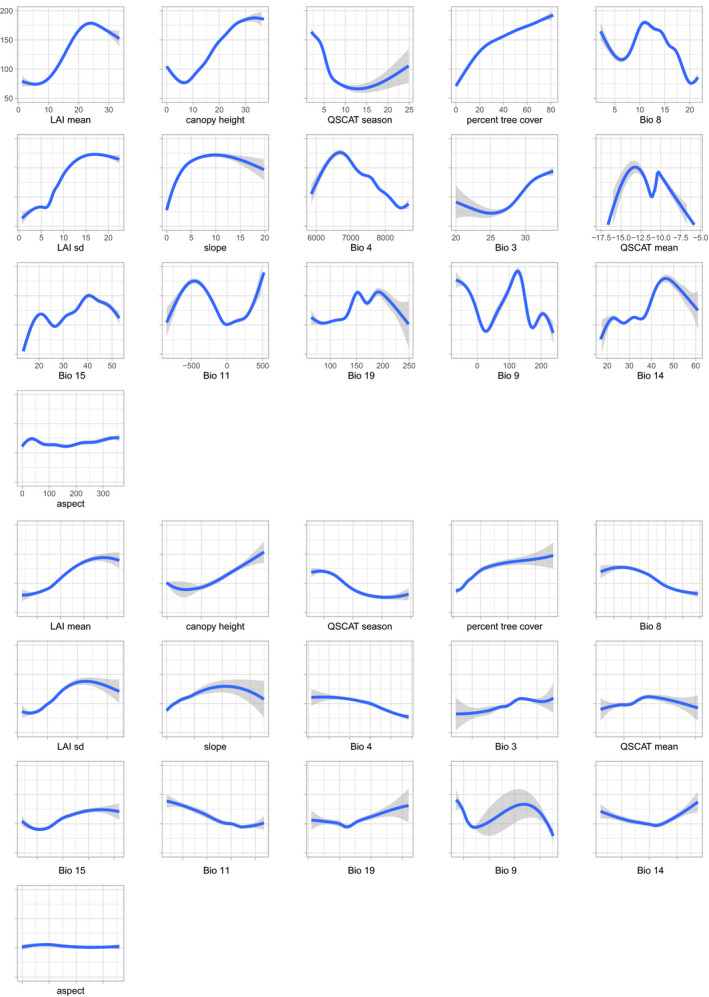
Overall (top panels) and marginal (bottom panels) response curves for presence‐only model predictions for *Bombus lucorum*. Overall response curves were generated for each variable, while letting all other variables covary. In contrast, marginal response curves were created for each variable, while keeping all other variables at their median observed values

Interestingly, the most important variables in limiting each species' distribution overlapped between species. The top four variables for *B. terrestris* were seasonality in surface moisture (QSCAT season), mean temperature of the wettest quarter (Bio 8), mean leaf area index (LAI mean), and temperature seasonality (Bio 4; Table 5). For *B. lucorum*, these variables comprised mean leaf area index (LAI mean), canopy height, seasonality in surface moisture (QSCAT season), and percent tree cover (Tree; Table 6), subsequently followed by mean temperature of the wettest quarter (Bio 8). For both species, the ranking of variables by their importance was largely consistent between modeling approaches. The main difference in the response curves between the two species is that those for *B. lucorum* are generally much steeper than those for *B. terrestris*, suggesting a stronger influence of the environment on *B. lucorum* (Figures 2 and 3). This difference is particularly pronounced for the top most important variables that were not overlapping between species, that is, percent tree cover and canopy height.

**TABLE 5:** Variable importance scores for ESMs based on presence‐only data for *Bombus terrestris*

Variable	ANN	GLM	MAXENT.P	ENS
QSCAT season	0.140	0.099	0.088	0.109
Bio 8	0.104	0.088	0.098	0.096
LAI mean	0.083	0.067	0.088	0.079
Bio 4	0.055	0.080	0.085	0.073
Bio 3	0.047	0.080	0.091	0.073
LAI sd	0.068	0.057	0.060	0.062
Slope	0.071	0.053	0.062	0.062
Bio 11	0.049	0.059	0.064	0.057
Bio 9	0.063	0.055	0.050	0.056
Bio 19	0.045	0.063	0.059	0.056
QSCAT mean	0.079	0.042	0.032	0.051
Bio 14	0.056	0.048	0.046	0.050
Canopy height	0.038	0.053	0.054	0.049
Tree	0.036	0.062	0.037	0.046
Bio 15	0.040	0.049	0.040	0.043
Aspect	0.023	0.045	0.046	0.038

Note

Scores for CTA are not included, because of its low model performance. MAXENT.P is the MAXENT.Phillips models. ENS is the ensemble of small models. See Table 1 for the meaning of the variables.

**TABLE 6:** Variable importance scores for ESMs based on presence‐only data for *Bombus lucorum*

Variable	ANN	GLM	MAXENT.P	ENS
LAI mean	0.091	0.088	0.092	0.090
Canopy height	0.082	0.089	0.083	0.085
QSCAT season	0.087	0.079	0.080	0.082
Tree	0.086	0.075	0.080	0.081
Bio 8	0.068	0.081	0.073	0.074
LAI sd	0.078	0.069	0.075	0.074
Slope	0.080	0.055	0.066	0.067
Bio 4	0.060	0.062	0.055	0.059
Bio 3	0.063	0.054	0.045	0.054
QSCAT mean	0.072	0.048	0.042	0.054
Bio 15	0.048	0.052	0.056	0.052
Bio 11	0.041	0.051	0.054	0.049
Bio 19	0.040	0.053	0.052	0.048
Bio 9	0.040	0.047	0.052	0.046
Bio 14	0.038	0.045	0.046	0.043
Aspect	0.027	0.051	0.048	0.042

Note

Scores for CTA are not included, because of its low model performance. MAXENT.P is the MAXENT.Phillips models. ENS is the ensemble of small models. See Table 1 for the meaning of the variables.

Consistent with the response curves, *B. terrestris* was predicted to be widely distributed, with medium suitability in lowland areas (in the north of Bulgaria and south of Romania) and low suitability in the Danube Delta and at the highest elevations (Figure 1b). Very high suitability was predicted for Mediterranean Bulgaria, south of the Balkan Mountains. Conversely, the range of *B. lucorum* was predicted to be much more restricted to higher elevations (the Carpathian Mountains and surrounding lowlands and the Balkan, Rila, Rhodope, and Pirin Mountains; Figure 1c).

### 3.3 Relative abundance models

**FIGURE 4:**
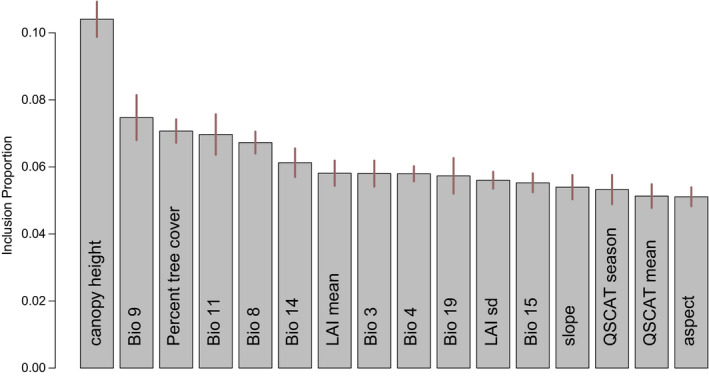
Variable importance inferred from a BART model for the relative abundance of *Bombus lucorum.* This model had a coefficient > 0.9 in the ensemble model, and it was the single best performing one in a nested cross‐validation analysis. We therefore used its robust estimate of variable importance to assess the contribution of each variable in the overall ensemble

**FIGURE 5:**
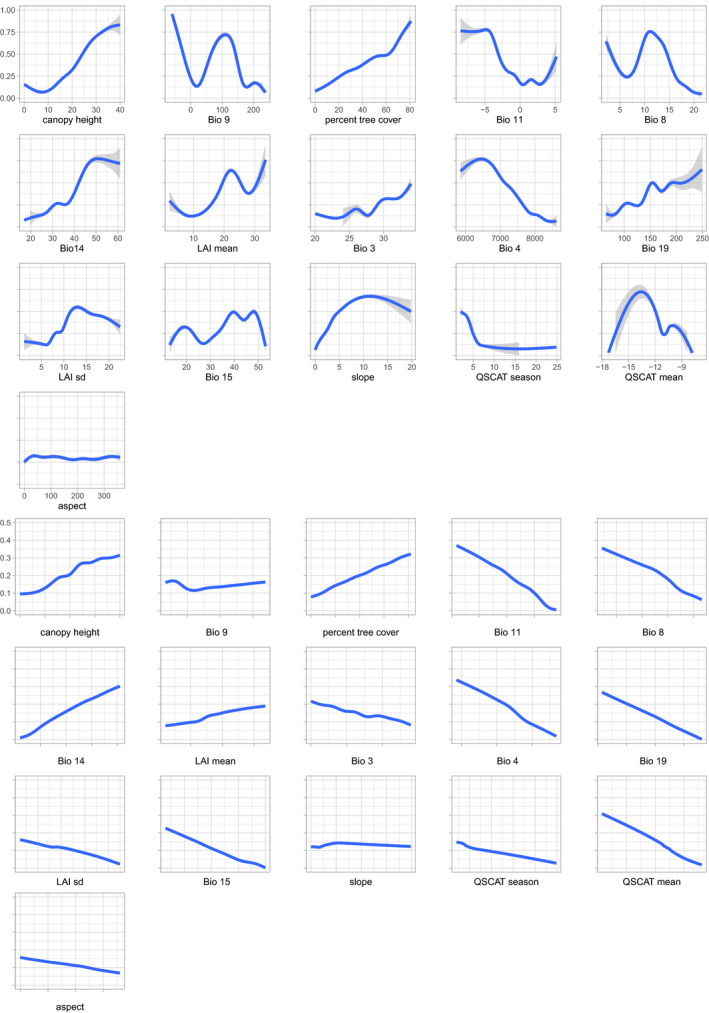
Overall (top panels) and marginal (bottom panels) response curves for relative abundance model predictions. Overall response curves were generated for each variable, while letting all other variables covary. In contrast, marginal response curves were created for each variable, while keeping all other variables at their median observed values

Models of relative abundance that were corrected for sampling bias performed considerably worse than uncorrected models (corrected models: BART coefficient = 0.885, BART risk = 0.404, GLM coefficient = 0.115, and GLM risk = 0.682). We therefore report results for uncorrected models from here onwards. The only two models included in the ensemble were GAM (coefficient = 0.084, risk = 0.199) and BART (coefficient = 0.916, risk = 0.093). K‐fold nested cross‐validation with fivefold suggested that the single best model was BART, which performed even slightly better than the ensemble model, yet statistically nonsignificant (Table 7). Because of the high weight of the BART model, we evaluated variable importance based on BART only, providing a robust posterior importance score (Chipman et al., 2010; Hernández, Raftery, Pennington, & Parnell, 2018). The top most important variable was canopy height, subsequently followed by percent tree cover and three temperature variables (Figure 4), which is broadly consistent with the results for the presence‐only data. Overall and marginal response curves suggest that *B. lucorum* is more abundant in more densely vegetated, wet and cool areas (Figure 5).

**TABLE 7:** Risk scores of fivefold cross‐validated models of relative abundance, with mean, standard error, minimum, and maximum values

Algorithm	Mean	SE	Min	Max
SuperLearner	0.096303	0.020817	0.04578	0.15106
MEAN	0.162742	0.022342	0.130919	0.22429
GLM	0.137423	0.033966	0.058933	0.22817
GAM	0.137423	0.033966	0.058933	0.22817
BART	0.095774	0.019007	0.052584	0.1495
RF	0.096686	0.021774	0.043806	0.15481
ANN	0.162742	0.022342	0.130919	0.22429

Note

The lower the risk, the better the model performance. The single best model was the BART model. SuperLearner is the ensemble of all models.

**FIGURE 6:**
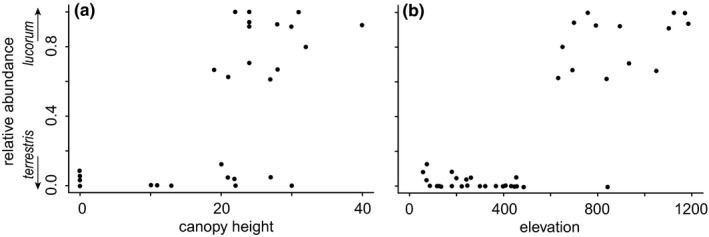
Scatterplots of the observed relative abundance of *Bombus lucorum* as a function of (a) canopy height, the most important variable in the BART model, and (b) elevation. Elevation was not entered in our models, because it was correlated to many environmental variables

To gain further insight in how our two target species differ in their preferred habitat conditions, we visually inspected scatterplots of the relative abundance of *B. lucorum* as a function of the most important variable, canopy height. We noted that the major mountain ranges in Romania and Bulgaria are a prominent feature in our distribution maps, which is consistent with previous descriptions of occurrence patterns. Although we dropped elevation from our analyses because of its high VIF, we also created a scatter plot of relative abundance versus elevation. Visual inspection of these plots suggested that *B. lucorum* does not occur in unforested areas with a canopy height under ~20 m (Figure 6a). Yet, the dichotomy between species is particularly striking for elevation, where *B. lucorum* is almost completely absent below 600 m, but makes up the majority of the two species at higher elevations (Figure 6b). Hence, elevation captures the combined influence of correlated environmental variables on limiting the distribution of *B. lucorum* particularly well.

## 4 DISCUSSION

Here, we modeled the distributions and relative abundance of two cryptic bumble bee species in Bulgaria and Romania from samples that were assigned to one of the species using a long fragment of the COI gene for genetic identification. We demonstrated that even though there is a certain degree of overlap between the ranges of the two species, *B. lucorum* has a much more restricted distribution than *B. terrestris*. Our models suggested that both vegetation and climate variables are key factors in determining their distributions. These results are concordant with previous studies for *B. lucorum* suggesting that it prefers closed habitat near forests (Bossert et al., 2016; Svensson et al., 2000). Our findings also support observations that *B. lucorum* occurs at higher elevations (Ban‐Calefariu & Sárospataki, 2007; Bossert et al., 2016; Goulson et al., 2008b; Ploquin, Herrera, & Obeso, 2013; Tomozei, 2006), which was suggested to be the result of an adaptation to colder climates (Benton, 2006). Indeed, in northern Europe, *B. lucorum* generally occurs in colder areas, where it at least partly substitutes *B. terrestris* (Rasmont, Franzen, et al., 2015). Many environmental variables change along an elevation gradient, and elevation itself is unlikely to determine the distribution of these species, but rather its covariates (Bossert et al., 2016). In our study, mean temperature of the coldest quarter (Bio 11), canopy height, percent tree cover, and mean leaf area index (LAI mean) were particularly highly correlated with elevation (Pearson correlation coefficient > 0.6). Hence, the distribution of *B. lucorum* is clearly restricted to the mountainous areas in Bulgaria and Romania (Figure 1c,d), where temperatures are lower, precipitation is higher, and where most of the forest is remaining.

In contrast, our findings for *B. terrestris* suggest that it is not as restricted to open habitat as previously thought (Bossert et al., 2016; Svensson et al., 2000), but rather is a generalist species, occurring in open as well as more densely vegetated areas. This notion is also apparent in our maps, showing a wide distribution for *B. terrestris*.

Interestingly, our presence‐only species distribution models showed a considerable overlap between the ranges of *B. terrestris* and *B. lucorum*, but analyses of their relative abundance evoke a much stronger separation between these species. Although the use of relative abundance does not allow for conclusions regarding the absolute abundance of either one of the species, the large range of relative abundance values for *B. lucorum*, spanning from 0 to 0.94, suggests that the two species replace one another across an environmental gradient. Thus, the inclusion of abundance enhances our understanding of the distribution of these species based on presence‐only models.

Although the conservation status of our study species across the European continent is “least concern” (Rasmont, Roberts, Cederberg, Radchenko, & Michez, 2015a, 2015b), they are “vulnerable” or “nearly threatened” in a few countries (Kosior et al., 2007). A previous cross‐continent study suggested that both *B. terrestris* and *B. lucorum* may suffer from range contractions under future climate change (Rasmont, Franzen, et al., 2015). The study by Rasmont, Franzen, et al. (2015) provides a great overview of overall distributions and risks posed by climate change. Yet, such large‐scale models of species distributions, spanning major latitudinal and environmental gradients, and based on climate variables only, may be of limited use at intermediate to smaller spatial extents. Indeed, we found that vegetation characteristics were among the most important variables explaining the distribution and relative abundance of our study species, and it will be difficult to predict how these variables will change in the future, both as a result of climate change, as well as due to changes in forest management. We did not proceed with an attempt to predict the distribution of *B. lucorum* onto future climate conditions, because a model based on only current climate conditions failed to even broadly resemble that based on both climate and vegetation variables (not shown). Moreover, populations are likely adapted to local and regional conditions, and may not respond the same to changing environmental conditions. Our study provides further insight by teasing apart the habitat preferences of these species in southeastern Europe, providing higher resolution range maps that are probably more relevant for the region, where the distribution of *B. lucorum* is assumed to be rather disjunct. Despite the complexity of predicting future changes in vegetation characteristics, the difference in habitat requirements between these species is expected to have implications for the way they respond to changing climate conditions. Our finding that *B. lucorum* is rather restricted in its suitable habitat conditions compared to *B. terrestris*, may suggest that it is more vulnerable to climate change than the latter.

We genetically identified a large number of individuals of two closely related bumble bee species sampled at many sites and modeled their distributions and gained insight into their habitat requirements. We showed that *B. terrestris* is rather a generalist species, whereas *B. lucorum* is restricted to colder and wetter climates in forested areas, which in our study area primarily occur at higher elevations. We support the emerging recognition of the importance of abundance data in species distribution modeling. Despite the overlap in occurrence suggested by presence‐only data, their relative abundance gradually changes along a major environmental gradient, with one of the species being virtually absent at the extreme ends of this gradient. Our study contributes to the urgent need to fill a major gap of knowledge in the distribution and ecology of these species that can help facilitate the assessment of their conservation status and the development of management plans where necessary.

## CONFLICT OF INTEREST

None declared.

## AUTHOR CONTRIBUTION

**Julia Geue:** Conceptualization (equal); Data curation (equal); Formal analysis (equal); Investigation (equal); Methodology (equal); Software (equal); Visualization (equal); Writing‐original draft (lead); Writing‐review & editing (lead). **Henri Thomassen:** Conceptualization (equal); Data curation (equal); Funding acquisition (lead); Investigation (supporting); Methodology (supporting); Project administration (lead); Software (equal); Supervision (equal); Validation (equal); Visualization (equal); Writing‐original draft (supporting); Writing‐review & editing (supporting).

## Supporting information

Fig S1

Table S1

## Data Availability

DNA sequences are available from Genbank, accession no. MT174625–MT175367. GIS layers of environmental variables and Variance Inflation Factor (VIF) scores are available through Dryad https://doi.org/10.5061/dryad.18931zcst.
