# Supplementary material for: Unraveling the habitat preferences of two closely related bumble bee species in Eastern Europe
Source: Ecol Evol. 2020 Apr 15;10(11):4773–90. doi: 10.1002/ece3.6232 (PMC7297791; doi:10.1002/ece3.6232)
Supplement: Supplementary file 2 — Table S1 [file ECE3-10-4773-s002.docx]

Table S1 - Detailed sample list (sorted by location). For each sample, the sampling location and geographical coordinates, the collection date, accession number, blast scores (GenBank (NIH genetic sequences database)) and the accession number of the reference each individual best matched are provided.

| **Location** | **Latitude** | **Longitude** | **Voucher Code** | **Collection date** | **Accession No.** | **Max Score** | **Total Score** | **Query Cover** | **E value** | ***%* Ident** | **Accession No. of reference** | **Species** |
| --- | --- | --- | --- | --- | --- | --- | --- | --- | --- | --- | --- | --- |
| Baita Plai | 46.46871 | 22.61674 | Bt_2014_128 | 01.07.2014 | MT175281 | 924 | 924 | 94% | 0.0 | 100.00% | AY181170.1 | *B.terrestris* |
| Baita Plai | 46.46871 | 22.61674 | Bt_2014_129 | 01.07.2014 | MT175282 | 852 | 852 | 100% | 0.0 | 100.00% | NC_045179.1 | *B.terrestris* |
| Baita Plai | 46.46871 | 22.61674 | Bt_2014_130 | 01.07.2014 | MT175283 | 1653 | 1653 | 100% | 0.0 | 98.31% | AY181170.1 | *B.terrestris* |
| Baita Plai | 46.46871 | 22.61674 | Bt_2014_131 | 01.07.2014 | MT175284 | 1517 | 1517 | 92% | 0.0 | 96.66% | NC_045179.1 | *B.terrestris* |
| Baita Plai | 46.46871 | 22.61674 | Bt_2014_132 | 01.07.2014 | MT175285 | 1670 | 1670 | 100% | 0.0 | 98.52% | AY181170.1 | *B.terrestris* |
| Baita Plai | 46.46871 | 22.61674 | Bt_2014_133 | 01.07.2014 | MT175286 | 1352 | 1352 | 100% | 0.0 | 99.73% | AY181170.1 | *B.terrestris* |
| Baita Plai | 46.46871 | 22.61674 | Bt_2014_134 | 01.07.2014 | MT175287 | 1714 | 1714 | 100% | 0.0 | 98.76% | NC_045179.1 | *B.terrestris* |
| Baita Plai | 46.46871 | 22.61674 | Bt_2014_135 | 01.07.2014 | MT175288 | 865 | 964 | 100% | 0.0 | 100.00% | AY181170.1 | *B.terrestris* |
| Baita Plai | 46.46871 | 22.61674 | Bt_2014_136 | 01.07.2014 | MT175289 | 654 | 654 | 100% | 0.0 | 94.60% | AY181170.1 | *B.terrestris* |
| Baita Plai | 46.46871 | 22.61674 | Bt_2014_137 | 01.07.2014 | MT175290 | 1565 | 1565 | 100% | 0.0 | 96.47% | NC_045179. | *B.terrestris* |
| Baita Plai | 46.46871 | 22.61674 | Bt_2014_138 | 01.07.2014 | MT175291 | 1692 | 1692 | 99% | 0.0 | 99.05% | AY181170.1 | *B.terrestris* |
| Baita Plai | 46.46871 | 22.61674 | Bt_2014_139 | 01.07.2014 | MT175292 | 1729 | 1729 | 99% | 0.0 | 98.67% | NC_045179.1 | *B.terrestris* |
| Baita Plai | 46.46871 | 22.61674 | Bt_2014_140 | 01.07.2014 | MT175293 | 1650 | 1650 | 99% | 0.0 | 97.63% | NC_045179.1 | *B.terrestris* |
| Baita Plai | 46.46871 | 22.61674 | Bt_2014_141 | 01.07.2014 | MT175294 | 1563 | 1563 | 98% | 0.0 | 95.85% | NC_045179.1 | *B.terrestris* |
| Baita Plai | 46.46871 | 22.61674 | Bt_2014_142 | 01.07.2014 | MT175295 | 1635 | 1635 | 100% | 0.0 | 97.33% | NC_045179.1 | *B.terrestris* |
| Baita Plai | 46.46871 | 22.61674 | Bt_2014_143 | 01.07.2014 | MT175296 | 1738 | 1738 | 100% | 0.0 | 99.27% | NC_045179.1 | *B.terrestris* |
| Baita Plai | 46.46871 | 22.61674 | Bt_2014_144 | 01.07.2014 | MT175297 | 1698 | 1698 | 100% | 0.0 | 98.84% | NC_045179.1 | *B.terrestris* |
| Baita Plai | 46.46871 | 22.61674 | Bt_2014_146 | 01.07.2014 | MT175298 | 1672 | 1672 | 100% | 0.0 | 98.53% | NC_045179.1 | *B.terrestris* |
| Baita Plai | 46.46871 | 22.61674 | Bt_2014_147 | 01.07.2014 | MT175299 | 1628 | 1628 | 100% | 0.0 | 96.86% | NC_045179.1 | *B.terrestris* |
| Baita Plai | 46.46871 | 22.61674 | Bt_2014_148 | 01.07.2014 | MT175300 | 1712 | 1712 | 100% | 0.0 | 98.56% | NC_045179.1 | *B.terrestris* |
| Baita Plai | 46.46871 | 22.61674 | Bt_2014_149 | 01.07.2014 | MT175301 | 1744 | 1744 | 99% | 0.0 | 99.08% | NC_045179.1 | *B.terrestris* |
|  |  |  |  |  |  |  |  |  |  |  |  |  |
| Billed | 45.91412 | 20.94701 | 2017_Bombus_374 | 03.06.2017 | MT175109 | 1550 | 1550 | 100% | 0.0 | 97.11% | NC_045179.1 | *B.terrestris* |
| Billed | 45.91412 | 20.94701 | 2017_Bombus_375 | 03.06.2017 | MT175110 | 1138 | 1138 | 100% | 0.0 | 100.00% | NC_045179.1 | *B.terrestris* |
| Billed | 45.91412 | 20.94701 | 2017_Bombus_376 | 03.06.2017 | MT175111 | 1783 | 1783 | 99% | 0.0 | 99.39% | NC_045179.1 | *B.terrestris* |
| Billed | 45.91412 | 20.94701 | 2017_Bombus_377 | 03.06.2017 | MT175112 | 1413 | 1413 | 100% | 0.0 | 98.63% | NC_045179.1 | *B.terrestris* |
| Billed | 45.91412 | 20.94701 | 2017_Bombus_378 | 03.06.2017 | MT175113 | 1578 | 1578 | 100% | 0.0 | 97.93% | NC_045179.1 | *B.terrestris* |
| Billed | 45.91412 | 20.94701 | 2017_Bombus_379 | 03.06.2017 | MT175114 | 1646 | 1646 | 100% | 0.0 | 99.78% | NC_045179.1 | *B.terrestris* |
| Billed | 45.91412 | 20.94701 | 2017_Bombus_380 | 03.06.2017 | MT175115 | 1672 | 1672 | 99% | 0.0 | 99.46% | NC_045179.1 | *B.terrestris* |
| Billed | 45.91412 | 20.94701 | 2017_Bombus_381 | 03.06.2017 | MT175116 | 1598 | 1598 | 100% | 0.0 | 97.95% | NC_045179.1 | *B.terrestris* |
| Billed | 45.91412 | 20.94701 | 2017_Bombus_382 | 03.06.2017 | MT175117 | 1452 | 1452 | 100% | 0.0 | 97.24% | NC_045179.1 | *B.terrestris* |
|  |  |  |  |  |  |  |  |  |  |  |  |  |
| Blandesti | 47.71380 | 26.86323 | 2017_Bombus_042 | 07.05.2017 | MT174786 | 1844 | 1844 | 100% | 0.0 | 99.03% | NC_045179.1 | *B.terrestris* |
| Blandesti | 47.71380 | 26.86323 | 2017_Bombus_043 | 07.05.2017 | MT174787 | 1845 | 1845 | 99% | 0.0 | 99.60% | NC_045179.1 | *B.terrestris* |
| Blandesti | 47.71380 | 26.86323 | 2017_Bombus_044 | 07.05.2017 | MT174788 | 1380 | 1380 | 100% | 0.0 | 99.35% | AY181170.1 | *B.terrestris* |
| Blandesti | 47.71380 | 26.86323 | 2017_Bombus_045 | 07.05.2017 | MT174789 | 1834 | 1834 | 100% | 0.0 | 100.00% | NC_045179.1 | *B.terrestris* |
| Blandesti | 47.71380 | 26.86323 | 2017_Bombus_046 | 07.05.2017 | MT174790 | 1829 | 1829 | 99% | 0.0 | 99.80% | NC_045179.1 | *B.terrestris* |
| Blandesti | 47.71380 | 26.86323 | 2017_Bombus_047 | 07.05.2017 | MT174791 | 1144 | 1144 | 99% | 0.0 | 99.21% | AY181170.1 | *B.terrestris* |
| Blandesti | 47.71380 | 26.86323 | 2017_Bombus_048 | 07.05.2017 | MT174792 | 1855 | 1855 | 99% | 0.0 | 99.80% | NC_045179.1 | *B.terrestris* |
| Blandesti | 47.71380 | 26.86323 | 2017_Bombus_049 | 07.05.2017 | MT174793 | 1862 | 1862 | 100% | 0.0 | 99.32% | NC_045179.1 | *B.terrestris* |
| Blandesti | 47.71380 | 26.86323 | 2017_Bombus_050 | 07.05.2017 | MT174794 | 1840 | 1840 | 99% | 0.0 | 99.80% | NC_045179.1 | *B.terrestris* |
| Blandesti | 47.71380 | 26.86323 | 2017_Bombus_051 | 07.05.2017 | MT174795 | 1790 | 1790 | 98% | 0.0 | 99.00% | NC_045179.1 | *B.terrestris* |
| Blandesti | 47.71380 | 26.86323 | 2017_Bombus_052 | 07.05.2017 | MT174796 | 1827 | 1827 | 99% | 0.0 | 99.50% | NC_045179.1 | *B.terrestris* |
| Blandesti | 47.71380 | 26.86323 | 2017_Bombus_053 | 07.05.2017 | MT174797 | 1799 | 1799 | 100% | 0.0 | 99.30% | NC_045179.1 | *B.terrestris* |
| Blandesti | 47.71380 | 26.86323 | 2017_Bombus_054 | 07.05.2017 | MT174798 | 1742 | 1742 | 100% | 0.0 | 99.58% | AY181170.1 | *B.terrestris* |
| Blandesti | 47.71380 | 26.86323 | 2017_Bombus_055 | 07.05.2017 | MT174799 | 1877 | 1877 | 99% | 0.0 | 99.71% | NC_045179.1 | *B.terrestris* |
| Blandesti | 47.71380 | 26.86323 | 2017_Bombus_056 | 07.05.2017 | MT174800 | 1803 | 1803 | 99% | 0.0 | 99.30% | NC_045179.1 | *B.terrestris* |
| Blandesti | 47.71380 | 26.86323 | 2017_Bombus_057 | 07.05.2017 | MT174801 | 1838 | 1838 | 99% | 0.0 | 99.50% | NC_045179.1 | *B.terrestris* |
| Blandesti | 47.71380 | 26.86323 | 2017_Bombus_058 | 07.05.2017 | MT174802 | 1808 | 1808 | 100% | 0.0 | 98.92% | NC_045179.1 | *B.terrestris* |
| Blandesti | 47.71380 | 26.86323 | 2017_Bombus_059 | 07.05.2017 | MT174803 | 1775 | 1775 | 100% | 0.0 | 98.61% | NC_045179.1 | *B.terrestris* |
| Blandesti | 47.71380 | 26.86323 | 2017_Bombus_060 | 07.05.2017 | MT174804 | 1838 | 1838 | 99% | 0.0 | 99.51% | NC_045179.1 | *B.terrestris* |
| Blandesti | 47.71380 | 26.86323 | 2017_Bombus_061 | 07.05.2017 | MT174805 | 1838 | 1838 | 100% | 0.0 | 99.50% | NC_045179.1 | *B.terrestris* |
| Blandesti | 47.71380 | 26.86323 | 2017_Bombus_062 | 07.05.2017 | MT174806 | 1862 | 1862 | 100% | 0.0 | 99.61% | NC_045179.1 | *B.terrestris* |
| Blandesti | 47.71380 | 26.86323 | 2017_Bombus_063 | 07.05.2017 | MT174807 | 1807 | 1807 | 100% | 0.0 | 99.20% | NC_045179.1 | *B.terrestris* |
| Blandesti | 47.71380 | 26.86323 | 2017_Bombus_064 | 07.05.2017 | MT174808 | 1877 | 1877 | 100% | 0.0 | 99.90% | NC_045179.1 | *B.terrestris* |
| Blandesti | 47.71380 | 26.86323 | 2017_Bombus_065 | 07.05.2017 | MT174809 | 1862 | 1862 | 99% | 0.0 | 99.42% | NC_045179.1 | *B.terrestris* |
| Blandesti | 47.71380 | 26.86323 | 2017_Bombus_066 | 07.05.2017 | MT174810 | 1400 | 1400 | 100% | 0.0 | 99.61% | NC_045179.1 | *B.terrestris* |
| Blandesti | 47.71380 | 26.86323 | 2017_Bombus_067 | 07.05.2017 | MT174811 | 1834 | 1834 | 100% | 0.0 | 99.31% | NC_045179.1 | *B.terrestris* |
| Blandesti | 47.71380 | 26.86323 | 2017_Bombus_068 | 07.05.2017 | MT174812 | 1840 | 1840 | 100% | 0.0 | 99.60% | NC_045179.1 | *B.terrestris* |
| Blandesti | 47.71380 | 26.86323 | 2017_Bombus_069 | 07.05.2017 | MT174813 | 1857 | 1857 | 100% | 0.0 | 99.70% | NC_045179.1 | *B.terrestris* |
| Blandesti | 47.71380 | 26.86323 | 2017_Bombus_070 | 07.05.2017 | MT174814 | 1838 | 1838 | 99% | 0.0 | 99.51% | NC_045179.1 | *B.terrestris* |
| Blandesti | 47.71380 | 26.86323 | 2017_Bombus_071 | 07.05.2017 | MT174815 | 1781 | 1781 | 100% | 0.0 | 98.80% | NC_045179.1 | *B.terrestris* |
| Blandesti | 47.71380 | 26.86323 | 2017_Bombus_072 | 07.05.2017 | MT174816 | 1620 | 1620 | 100% | 0.0 | 99.66% | NC_045179.1 | *B.terrestris* |
| Blandesti | 47.71380 | 26.86323 | 2017_Bombus_073 | 07.05.2017 | MT174817 | 1816 | 1816 | 100% | 0.0 | 99.50% | NC_045179.1 | *B.terrestris* |
| Blandesti | 47.71380 | 26.86323 | 2017_Bombus_074 | 07.05.2017 | MT174818 | 1812 | 1812 | 100% | 0.0 | 99.01% | NC_045179.1 | *B.terrestris* |
|  |  |  |  |  |  |  |  |  |  |  |  |  |
| Brebu | 45.42815 | 21.97966 | 2017_Bombus_289 | 03.06.2017 | MT175027 | 1755 | 1755 | 100% | 0.0 | 99.58% | NC_045179.1 | *B.terrestris* |
| Brebu | 45.42815 | 21.97966 | 2017_Bombus_291 | 03.06.2017 | MT175028 | 1544 | 1544 | 100% | 0.0 | 99.76% | NC_045179.1 | *B.terrestris* |
| Brebu | 45.42815 | 21.97966 | 2017_Bombus_292 | 03.06.2017 | MT175029 | 1832 | 1832 | 99% | 0.0 | 99.60% | NC_045179.1 | *B.terrestris* |
| Brebu | 45.42815 | 21.97966 | 2017_Bombus_293 | 03.06.2017 | MT175030 | 1703 | 1703 | 100% | 0.0 | 99.78% | NC_045179.1 | *B.terrestris* |
| Brebu | 45.42815 | 21.97966 | 2017_Bombus_294 | 03.06.2017 | MT175031 | 1664 | 1664 | 99% | 0.0 | 99.67% | NC_045179.1 | *B.terrestris* |
| Brebu | 45.42815 | 21.97966 | 2017_Bombus_295 | 03.06.2017 | MT175032 | 1768 | 1768 | 100% | 0.0 | 99.39% | NC_045179.1 | *B.terrestris* |
| Brebu | 45.42815 | 21.97966 | 2017_Bombus_298 | 03.06.2017 | MT175033 | 1376 | 1376 | 100% | 0.0 | 99.87% | NC_045179.1 | *B.terrestris* |
| Brebu | 45.42815 | 21.97966 | 2017_Bombus_299 | 03.06.2017 | MT175034 | 1506 | 1506 | 100% | 0.0 | 98.60% | AY181170.1 | *B.terrestris* |
| Brebu | 45.42815 | 21.97966 | 2017_Bombus_300 | 03.06.2017 | MT175035 | 893 | 893 | 100% | 0.0 | 99.39% | KT164618.1 | *B.terrestris* |
| Brebu | 45.42815 | 21.97966 | 2017_Bombus_301 | 03.06.2017 | MT175036 | 1450 | 1450 | 99% | 0.0 | 99.38% | KT164681.1 | *B.lucorum* |
| Brebu | 45.42815 | 21.97966 | 2017_Bombus_302 | 03.06.2017 | MT175037 | 1685 | 1685 | 100% | 0.0 | 99.35% | NC_045179.1 | *B.terrestris* |
| Brebu | 45.42815 | 21.97966 | 2017_Bombus_303 | 03.06.2017 | MT175038 | 1539 | 1539 | 100% | 0.0 | 99.41% | NC_045179.1 | *B.terrestris* |
| Brebu | 45.42815 | 21.97966 | 2017_Bombus_304 | 03.06.2017 | MT175039 | 1535 | 1535 | 99% | 0.0 | 98.62% | NC_045179.1 | *B.terrestris* |
| Brebu | 45.42815 | 21.97966 | 2017_Bombus_305 | 03.06.2017 | MT175040 | 1736 | 1736 | 100% | 0.0 | 99.27% | NC_045179.1 | *B.terrestris* |
| Brebu | 45.42815 | 21.97966 | 2017_Bombus_306 | 03.06.2017 | MT175041 | 1801 | 1801 | 100% | 0.0 | 99.70% | NC_045179.1 | *B.terrestris* |
| Brebu | 45.42815 | 21.97966 | 2017_Bombus_307 | 03.06.2017 | MT175042 | 1847 | 1847 | 100% | 0.0 | 99.80% | NC_045179.1 | *B.terrestris* |
| Brebu | 45.42815 | 21.97966 | 2017_Bombus_308 | 03.06.2017 | MT175043 | 1585 | 1585 | 99% | 0.0 | 99.21% | NC_045179.1 | *B.terrestris* |
| Brebu | 45.42815 | 21.97966 | 2017_Bombus_309 | 03.06.2017 | MT175044 | 1810 | 1810 | 100% | 0.0 | 99.30% | NC_045179.1 | *B.terrestris* |
| Brebu | 45.42815 | 21.97966 | 2017_Bombus_310 | 03.06.2017 | MT175045 | 1531 | 1531 | 100% | 0.0 | 99.64% | AY181170.1 | *B.terrestris* |
| Brebu | 45.42815 | 21.97966 | 2017_Bombus_311 | 03.06.2017 | MT175046 | 1829 | 1829 | 100% | 0.0 | 99.60% | NC_045179.1 | *B.terrestris* |
| Brebu | 45.42815 | 21.97966 | 2017_Bombus_312 | 03.06.2017 | MT175047 | 1592 | 1592 | 100% | 0.0 | 96.86% | NC_045179.1 | *B.terrestris* |
|  |  |  |  |  |  |  |  |  |  |  |  |  |
| Burja | 43.02797 | 25.32507 | 2013_Bt_0091 | 24.05.2013 | MT174676 | 1858 | 1858 | 99% | 0.0 | 99.70% | NC_045179.1 | *B.terrestris* |
| Burja | 43.02797 | 25.32507 | 2013_Bt_0092 | 24.05.2013 | MT174677 | 1410 | 1410 | 100% | 0.0 | 100.00% | NC_045179.1 | *B.terrestris* |
| Burja | 43.02797 | 25.32507 | 2013_Bt_0093 | 24.05.2013 | MT174678 | 1794 | 1794 | 100% | 0.0 | 100.00% | NC_045179.1 | *B.terrestris* |
| Burja | 43.02797 | 25.32507 | 2013_Bt_0094 | 24.05.2013 | MT174679 | 1895 | 1895 | 100% | 0.0 | 99.90% | NC_045179.1 | *B.terrestris* |
| Burja | 43.02797 | 25.32507 | 2013_Bt_0095 | 24.05.2013 | MT174680 | 1858 | 1858 | 99% | 0.0 | 100.00% | AY181170.1 | *B.terrestris* |
|  |  |  |  |  |  |  |  |  |  |  |  |  |
| Carei | 47.69646 | 22.48073 | Bt_2014_219 | 07.07.2014 | MT175335 | 1810 | 1810 | 93% | 0.0 | 98.08% | NC_045179.1 | *B.terrestris* |
| Carei | 47.69646 | 22.48073 | Bt_2014_220 | 07.07.2014 | MT175336 | 1832 | 1832 | 92% | 0.0 | 98.65% | NC_045179. | *B.terrestris* |
| Carei | 47.69646 | 22.48073 | Bt_2014_221 | 07.07.2014 | MT175337 | 1842 | 1842 | 100% | 0.0 | 99.41% | NC_045179.1 | *B.terrestris* |
| Carei | 47.69646 | 22.48073 | Bt_2014_222 | 07.07.2014 | MT175338 | 1836 | 1836 | 99% | 0.0 | 99.70% | NC_045179. | *B.terrestris* |
| Carei | 47.69646 | 22.48073 | Bt_2014_223 | 07.07.2014 | MT175339 | 1825 | 1825 | 100% | 0.0 | 99.60% | NC_045179.1 | *B.terrestris* |
| Carei | 47.69646 | 22.48073 | Bt_2014_224 | 07.07.2014 | MT175340 | 1805 | 1805 | 99% | 0.0 | 99.40% | NC_045179.1 | *B.terrestris* |
| Carei | 47.69646 | 22.48073 | Bt_2014_225 | 07.07.2014 | MT175341 | 1818 | 1818 | 99% | 0.0 | 99.40% | NC_045179.1 | *B.terrestris* |
| Carei | 47.69646 | 22.48073 | Bt_2014_226 | 07.07.2014 | MT175342 | 1807 | 1807 | 100% | 0.0 | 99.11% | NC_045179.1 | *B.terrestris* |
| Carei | 47.69646 | 22.48073 | Bt_2014_227 | 07.07.2014 | MT175343 | 1825 | 1825 | 97% | 0.0 | 99.31% | NC_045179.1 | *B.terrestris* |
| Carei | 47.69646 | 22.48073 | Bt_2014_228 | 07.07.2014 | MT175344 | 1832 | 1832 | 100% | 0.0 | 99.41% | NC_045179.1 | *B.terrestris* |
| Carei | 47.69646 | 22.48073 | Bt_2014_229 | 07.07.2014 | MT175345 | 1805 | 1805 | 98% | 0.0 | 98.72% | NC_045179.1 | *B.terrestris* |
| Carei | 47.69646 | 22.48073 | Bt_2014_230 | 07.07.2014 | MT175346 | 1840 | 1840 | 100% | 0.0 | 99.70% | NC_045179.1 | *B.terrestris* |
| Carei | 47.69646 | 22.48073 | Bt_2014_232 | 07.07.2014 | MT175347 | 1836 | 1836 | 99% | 0.0 | 99.51% | NC_045179.1 | *B.terrestris* |
| Carei | 47.69646 | 22.48073 | Bt_2014_233 | 07.07.2014 | MT175348 | 1818 | 1818 | 99% | 0.0 | 99.40% | NC_045179. | *B.terrestris* |
| Carei | 47.69646 | 22.48073 | Bt_2014_234 | 07.07.2014 | MT175349 | 1831 | 1831 | 99% | 0.0 | 99.60% | NC_045179.1 | *B.terrestris* |
| Carei | 47.69646 | 22.48073 | Bt_2014_235 | 07.07.2014 | MT175350 | 1823 | 1823 | 97% | 0.0 | 99.31% | NC_045179.1 | *B.terrestris* |
| Carei | 47.69646 | 22.48073 | Bt_2014_236 | 07.07.2014 | MT175351 | 1844 | 1844 | 96% | 0.0 | 99.51% | NC_045179.1 | *B.terrestris* |
| Carei | 47.69646 | 22.48073 | Bt_2014_237 | 07.07.2014 | MT175352 | 1836 | 1836 | 99% | 0.0 | 99.60% | NC_045179.1 | *B.terrestris* |
| Carei | 47.69646 | 22.48073 | Bt_2014_238 | 07.07.2014 | MT175353 | 1860 | 1860 | 100% | 0.0 | 99.32% | NC_045179.1 | *B.terrestris* |
| Carei | 47.69646 | 22.48073 | Bt_2014_239 | 07.07.2014 | MT175354 | 1877 | 1877 | 100% | 0.0 | 99.23% | NC_045179.1 | *B.terrestris* |
| Carei | 47.69646 | 22.48073 | Bt_2014_240 | 07.07.2014 | MT175355 | 1845 | 1845 | 100% | 0.0 | 99.70% | NC_045179.1 | *B.terrestris* |
| Carei | 47.69646 | 22.48073 | Bt_2014_241 | 07.07.2014 | MT175356 | 1836 | 1836 | 100% | 0.0 | 98.93% | NC_045179.1 | *B.terrestris* |
| Carei | 47.69646 | 22.48073 | Bt_2014_242 | 07.07.2014 | MT175357 | 1832 | 1832 | 100% | 0.0 | 99.31% | NC_045179.1 | *B.terrestris* |
| Carei | 47.69646 | 22.48073 | Bt_2014_243 | 07.07.2014 | MT175358 | 1832 | 1832 | 99% | 0.0 | 99.60% | NC_045179. | *B.terrestris* |
|  |  |  |  |  |  |  |  |  |  |  |  |  |
| Cerna | 45.15962 | 22.80671 | 2013_Bt_0363 | 31.07.2013 | MT175238 | 1794 | 1794 | 99% | 0.0 | 99.30% | NC_045179.1 | *B.terrestris* |
| Cerna | 45.15962 | 22.80671 | 2013_Bt_0364 | 31.07.2013 | MT175239 | 1838 | 1838 | 97% | 0.0 | 99.70% | KT164681.1 | *B.lucorum* |
| Cerna | 45.15962 | 22.80671 | 2013_Bt_0365 | 31.07.2013 | MT175240 | 1838 | 1838 | 100% | 0.0 | 99.90% | NC_045179.1 | *B.terrestris* |
| Cerna | 45.15962 | 22.80671 | 2013_Bt_0366 | 31.07.2013 | MT175241 | 1829 | 1829 | 100% | 0.0 | 99.90% | NC_045179.1 | *B.terrestris* |
| Cerna | 45.15962 | 22.80671 | 2013_Bt_0367 | 31.07.2013 | MT175242 | 1816 | 1816 | 100% | 0.0 | 99.80% | KT164681.1 | *B.lucorum* |
| Cerna | 45.15962 | 22.80671 | 2013_Bt_0368 | 31.07.2013 | MT175243 | 1851 | 1851 | 99% | 0.0 | 100.00% | KT164681.1 | *B.lucorum* |
| Cerna | 45.15962 | 22.80671 | 2013_Bt_0369 | 31.07.2013 | MT175244 | 1820 | 1820 | 100% | 0.0 | 99.70% | KT164681.1 | *B.lucorum* |
| Cerna | 45.15962 | 22.80671 | 2013_Bt_0370 | 31.07.2013 | MT175245 | 1825 | 1825 | 99% | 0.0 | 99.90% | KT164681.1 | *B.lucorum* |
| Cerna | 45.15962 | 22.80671 | 2013_Bt_0371 | 31.07.2013 | MT175246 | 1818 | 1818 | 100% | 0.0 | 99.80% | KT164681.1 | *B.lucorum* |
| Cerna | 45.15962 | 22.80671 | 2013_Bt_0372 | 31.07.2013 | MT175247 | 1805 | 1805 | 98% | 0.0 | 99.40% | KT164681.1 | *B.lucorum* |
| Cerna | 45.15962 | 22.80671 | 2013_Bt_0373 | 31.07.2013 | MT175248 | 1827 | 1827 | 99% | 0.0 | 99.90% | KT164681.1 | *B.lucorum* |
| Cerna | 45.15962 | 22.80671 | 2013_Bt_0374 | 31.07.2013 | MT175249 | 1797 | 1797 | 100% | 0.0 | 99.90% | KT164681.1 | *B.lucorum* |
| Cerna | 45.15962 | 22.80671 | 2013_Bt_0375 | 31.07.2013 | MT175250 | 1814 | 1814 | 100% | 0.0 | 99.80% | KT164681.1 | *B.lucorum* |
| Cerna | 45.15962 | 22.80671 | 2013_Bt_0376 | 31.07.2013 | MT175251 | 1247 | 1247 | 94% | 0.0 | 94.94% | KT164681.1 | *B.lucorum* |
| Cerna | 45.15962 | 22.80671 | 2013_Bt_0377 | 31.07.2013 | MT175252 | 1832 | 1832 | 100% | 0.0 | 99.70% | KT164681.1 | *B.lucorum* |
| Cerna | 45.15962 | 22.80671 | 2013_Bt_0378 | 31.07.2013 | MT175253 | 1832 | 1832 | 100% | 0.0 | 100.00% | KT164681.1 | *B.lucorum* |
| Cerna | 45.15962 | 22.80671 | 2013_Bt_0379 | 31.07.2013 | MT175254 | 1840 | 1840 | 97% | 0.0 | 99.03% | NC_045179.1 | *B.terrestris* |
| Cerna | 45.15962 | 22.80671 | 2013_Bt_0380 | 31.07.2013 | MT175255 | 1840 | 1840 | 100% | 0.0 | 99.90% | KT164681.1 | *B.lucorum* |
| Cerna | 45.15962 | 22.80671 | 2013_Bt_0381 | 31.07.2013 | MT175256 | 1836 | 1836 | 100% | 0.0 | 99.90% | KT164681.1 | *B.lucorum* |
| Cerna | 45.15962 | 22.80671 | 2013_Bt_0382 | 31.07.2013 | MT175257 | 1838 | 1838 | 99% | 0.0 | 99.70% | KT164681.1 | *B.lucorum* |
|  |  |  |  |  |  |  |  |  |  |  |  |  |
| Coastra | 45.14758 | 24.22260 | Bt_2015_020 | 23.08.2015 | MT175359 | 1530 | 1530 | 100% | 0.0 | 95.38% | NC_045179.1 | *B.terrestris* |
| Coastra | 45.14758 | 24.22260 | Bt_2015_021 | 23.08.2015 | MT175360 | 1664 | 1664 | 99% | 0.0 | 97.44% | AY181170.1 | *B.terrestris* |
| Coastra | 45.14758 | 24.22260 | Bt_2015_022 | 23.08.2015 | MT175361 | 1768 | 1768 | 99% | 0.0 | 99.59% | NC_045179.1 | *B.terrestris* |
| Coastra | 45.14758 | 24.22260 | Bt_2015_023 | 23.08.2015 | MT175362 | 1882 | 1882 | 98% | 0.0 | 99.14% | NC_045179.1 | *B.terrestris* |
| Coastra | 45.14758 | 24.22260 | Bt_2015_024 | 23.08.2015 | MT175363 | 1692 | 1692 | 99% | 0.0 | 97.41% | NC_045179.1 | *B.terrestris* |
| Coastra | 45.14758 | 24.22260 | Bt_2015_025 | 23.08.2015 | MT175364 | 1764 | 1764 | 100% | 0.0 | 99.79% | NC_045179.1 | *B.terrestris* |
| Coastra | 45.14758 | 24.22260 | Bt_2015_026 | 23.08.2015 | MT175365 | 1504 | 1504 | 92% | 0.0 | 95.80% | AY181170.1 | *B.terrestris* |
| Coastra | 45.14758 | 24.22260 | Bt_2015_028 | 23.08.2015 | MT175366 | 1064 | 1064 | 99% | 0.0 | 99.83% | AY181170.1 | *B.terrestris* |
| Coastra | 45.14758 | 24.22260 | Bt_2015_030 | 23.08.2015 | MT175367 | 1718 | 1718 | 100% | 0.0 | 99.16% | NC_045179.1 | *B.terrestris* |
|  |  |  |  |  |  |  |  |  |  |  |  |  |
| Corbeni | 45.29905 | 24.60912 | 2013_Bt_0158 | 02.06.2013 | MT174722 | 1696 | 1696 | 100% | 0.0 | 99.89% | KT164681.1 | *B.lucorum* |
| Corbeni | 45.29905 | 24.60912 | 2013_Bt_0159 | 02.06.2013 | MT174723 | 1847 | 1847 | 100% | 0.0 | 99.51% | NC_045179.1 | *B.terrestris* |
| Corbeni | 45.29905 | 24.60912 | 2013_Bt_0161 | 02.06.2013 | MT174724 | 1888 | 1888 | 99% | 0.0 | 99.90% | KT164681.1 | *B.lucorum* |
| Corbeni | 45.29905 | 24.60912 | 2013_Bt_0162 | 02.06.2013 | MT174725 | 1860 | 1860 | 99% | 0.0 | 99.61% | KT164681.1 | *B.lucorum* |
| Corbeni | 45.29905 | 24.60912 | 2013_Bt_0163 | 02.06.2013 | MT174726 | 1834 | 1834 | 99% | 0.0 | 99.60% | NC_045179.1 | *B.terrestris* |
| Corbeni | 45.29905 | 24.60912 | 2013_Bt_0165 | 02.06.2013 | MT174727 | 1860 | 1860 | 100% | 0.0 | 99.51% | NC_045179.1 | *B.terrestris* |
| Corbeni | 45.29905 | 24.60912 | 2013_Bt_0166 | 02.06.2013 | MT174728 | 1866 | 1866 | 100% | 0.0 | 99.71% | NC_045179.1 | *B.terrestris* |
| Corbeni | 45.29905 | 24.60912 | 2013_Bt_0167 | 02.06.2013 | MT174729 | 1860 | 1860 | 94% | 0.0 | 99.61% | KT164681.1 | *B.lucorum* |
| Corbeni | 45.29905 | 24.60912 | 2013_Bt_0168 | 02.06.2013 | MT174730 | 1881 | 1881 | 100% | 0.0 | 99.33% | KT164681.1 | *B.lucorum* |
| Corbeni | 45.29905 | 24.60912 | 2013_Bt_0170 | 02.06.2013 | MT174731 | 1755 | 1755 | 100% | 0.0 | 100.00% | KT164681.1 | *B.lucorum* |
| Corbeni | 45.29905 | 24.60912 | 2013_Bt_0171 | 02.06.2013 | MT174732 | 1864 | 1864 | 99% | 0.0 | 99.61% | KT164681.1 | *B.lucorum* |
| Corbeni | 45.29905 | 24.60912 | 2013_Bt_0172 | 02.06.2013 | MT174733 | 1857 | 1857 | 100% | 0.0 | 99.70% | KT164681.1 | *B.lucorum* |
| Corbeni | 45.29905 | 24.60912 | 2013_Bt_0174 | 02.06.2013 | MT174734 | 1591 | 1591 | 100% | 0.0 | 100.00% | AY181170.1 | *B.terrestris* |
|  |  |  |  |  |  |  |  |  |  |  |  |  |
| Dobrovat | 46.99043 | 27.65404 | 2017_Bombus_075 | 08.05.2017 | MT174819 | 1829 | 1829 | 99% | 0.0 | 99.70% | NC_045179.1 | *B.terrestris* |
| Dobrovat | 46.99043 | 27.65404 | 2017_Bombus_076 | 08.05.2017 | MT174820 | 1749 | 1749 | 100% | 0.0 | 98.31% | NC_045179.1 | *B.terrestris* |
| Dobrovat | 46.99043 | 27.65404 | 2017_Bombus_077 | 08.05.2017 | MT174821 | 1842 | 1842 | 100% | 0.0 | 99.51% | NC_045179.1 | *B.terrestris* |
| Dobrovat | 46.99043 | 27.65404 | 2017_Bombus_078 | 08.05.2017 | MT174822 | 1821 | 1821 | 100% | 0.0 | 99.60% | NC_045179.1 | *B.terrestris* |
| Dobrovat | 46.99043 | 27.65404 | 2017_Bombus_079 | 08.05.2017 | MT174823 | 1836 | 1836 | 99% | 0.0 | 99.80% | NC_045179.1 | *B.terrestris* |
| Dobrovat | 46.99043 | 27.65404 | 2017_Bombus_080 | 08.05.2017 | MT174824 | 1489 | 1489 | 100% | 0.0 | 99.04% | NC_045179.1 | *B.terrestris* |
| Dobrovat | 46.99043 | 27.65404 | 2017_Bombus_081 | 08.05.2017 | MT174825 | 1786 | 1786 | 100% | 0.0 | 99.00% | NC_045179.1 | *B.terrestris* |
| Dobrovat | 46.99043 | 27.65404 | 2017_Bombus_082 | 08.05.2017 | MT174826 | 1814 | 1814 | 100% | 0.0 | 99.21% | KT164681.1 | *B.lucorum* |
| Dobrovat | 46.99043 | 27.65404 | 2017_Bombus_083 | 08.05.2017 | MT174827 | 1711 | 1711 | 99% | 0.0 | 98.85% | NC_045179.1 | *B.terrestris* |
| Dobrovat | 46.99043 | 27.65404 | 2017_Bombus_084 | 08.05.2017 | MT174828 | 948 | 948 | 100% | 0.0 | 99.81% | NC_045179.1 | *B.terrestris* |
| Dobrovat | 46.99043 | 27.65404 | 2017_Bombus_085 | 08.05.2017 | MT174829 | 1712 | 1712 | 100% | 0.0 | 99.37% | NC_045179.1 | *B.terrestris* |
| Dobrovat | 46.99043 | 27.65404 | 2017_Bombus_086 | 08.05.2017 | MT174830 | 1820 | 1820 | 100% | 0.0 | 99.40% | NC_045179.1 | *B.terrestris* |
| Dobrovat | 46.99043 | 27.65404 | 2017_Bombus_087 | 08.05.2017 | MT174831 | 1784 | 1784 | 100% | 0.0 | 99.69% | AY181170.1 | *B.terrestris* |
| Dobrovat | 46.99043 | 27.65404 | 2017_Bombus_088 | 08.05.2017 | MT174832 | 1868 | 1868 | 100% | 0.0 | 99.90% | NC_045179.1 | *B.terrestris* |
| Dobrovat | 46.99043 | 27.65404 | 2017_Bombus_089 | 08.05.2017 | MT174833 | 1724 | 1724 | 100% | 0.0 | 99.47% | AY181170.1 | *B.terrestris* |
| Dobrovat | 46.99043 | 27.65404 | 2017_Bombus_090 | 08.05.2017 | MT174834 | 1363 | 1363 | 100% | 0.0 | 99.21% | AY181170.1 | *B.terrestris* |
| Dobrovat | 46.99043 | 27.65404 | 2017_Bombus_091 | 08.05.2017 | MT174835 | 1487 | 1487 | 100% | 0.0 | 99.51% | AY181170.1 | *B.terrestris* |
| Dobrovat | 46.99043 | 27.65404 | 2017_Bombus_092 | 08.05.2017 | MT174836 | 1223 | 1223 | 99% | 0.0 | 99.70% | NC_045179.1 | *B.terrestris* |
| Dobrovat | 46.99043 | 27.65404 | 2017_Bombus_093 | 08.05.2017 | MT174837 | 1674 | 1674 | 99% | 0.0 | 99.14% | NC_045179.1 | *B.terrestris* |
| Dobrovat | 46.99043 | 27.65404 | 2017_Bombus_095 | 08.05.2017 | MT174838 | 929 | 929 | 99% | 0.0 | 99.42% | NC_045179.1 | *B.terrestris* |
| Dobrovat | 46.99043 | 27.65404 | 2017_Bombus_096 | 08.05.2017 | MT174839 | 1188 | 1188 | 100% | 0.0 | 99.69% | NC_045179.1 | *B.terrestris* |
| Dobrovat | 46.99043 | 27.65404 | 2017_Bombus_097 | 08.05.2017 | MT174840 | 1387 | 1387 | 100% | 0.0 | 99.35% | NC_045179.1 | *B.terrestris* |
| Dobrovat | 46.99043 | 27.65404 | 2017_Bombus_098 | 08.05.2017 | MT174841 | 1714 | 1714 | 99% | 0.0 | 98.08% | AY181170.1 | *B.terrestris* |
| Dobrovat | 46.99043 | 27.65404 | 2017_Bombus_099 | 08.05.2017 | MT174842 | 985 | 985 | 99% | 0.0 | 99.63% | AY181170.1 | *B.terrestris* |
| Dobrovat | 46.99043 | 27.65404 | 2017_Bombus_100 | 08.05.2017 | MT174843 | 1541 | 1541 | 99% | 0.0 | 99.30% | NC_045179.1 | *B.terrestris* |
|  |  |  |  |  |  |  |  |  |  |  |  |  |
| Drӑgusani | 46.29929 | 26.97973 | 2017_Bombus_102 | 10.05.2017 | MT174844 | 1751 | 1751 | 100% | 0.0 | 98.69% | NC_045179.1 | *B.terrestris* |
| Drӑgusani | 46.29929 | 26.97973 | 2017_Bombus_103 | 10.05.2017 | MT174845 | 1655 | 1655 | 99% | 0.0 | 97.25% | NC_045179. | *B.terrestris* |
| Drӑgusani | 46.29929 | 26.97973 | 2017_Bombus_104 | 10.05.2017 | MT174846 | 1812 | 1812 | 100% | 0.0 | 99.40% | NC_045179.1 | *B.terrestris* |
| Drӑgusani | 46.29929 | 26.97973 | 2017_Bombus_106 | 10.05.2017 | MT174847 | 1327 | 1327 | 100% | 0.0 | 99.59% | NC_045179.1 | *B.terrestris* |
| Drӑgusani | 46.29929 | 26.97973 | 2017_Bombus_108 | 10.05.2017 | MT174848 | 1552 | 1552 | 100% | 0.0 | 99.53% | KT164681.1 | *B.lucorum* |
| Drӑgusani | 46.29929 | 26.97973 | 2017_Bombus_109 | 10.05.2017 | MT174849 | 1620 | 1620 | 100% | 0.0 | 99.55% | NC_045179.1 | *B.terrestris* |
| Drӑgusani | 46.29929 | 26.97973 | 2017_Bombus_110 | 10.05.2017 | MT174850 | 1674 | 1674 | 99% | 0.0 | 99.56% | NC_045179.1 | *B.terrestris* |
| Drӑgusani | 46.29929 | 26.97973 | 2017_Bombus_111 | 10.05.2017 | MT174851 | 1552 | 1552 | 100% | 0.0 | 99.30% | AY181170.1 | *B.terrestris* |
| Drӑgusani | 46.29929 | 26.97973 | 2017_Bombus_112 | 10.05.2017 | MT174852 | 1417 | 1417 | 99% | 0.0 | 99.12% | NC_045179.1 | *B.terrestris* |
| Drӑgusani | 46.29929 | 26.97973 | 2017_Bombus_113 | 10.05.2017 | MT174853 | 1491 | 1491 | 99% | 0.0 | 98.81% | NC_045179.1 | *B.terrestris* |
| Drӑgusani | 46.29929 | 26.97973 | 2017_Bombus_114 | 10.05.2017 | MT174854 | 1554 | 1554 | 99% | 0.0 | 99.08% | AY181170.1 | *B.terrestris* |
| Drӑgusani | 46.29929 | 26.97973 | 2017_Bombus_115 | 10.05.2017 | MT174855 | 1786 | 1786 | 99% | 0.0 | 99.10% | NC_045179.1 | *B.terrestris* |
| Drӑgusani | 46.29929 | 26.97973 | 2017_Bombus_116 | 10.05.2017 | MT174856 | 1589 | 1589 | 100% | 0.0 | 99.43% | AY181170.1 | *B.terrestris* |
| Drӑgusani | 46.29929 | 26.97973 | 2017_Bombus_117 | 10.05.2017 | MT174857 | 1628 | 1628 | 100% | 0.0 | 99.55% | AY181170.1 | *B.terrestris* |
| Drӑgusani | 46.29929 | 26.97973 | 2017_Bombus_118 | 10.05.2017 | MT174858 | 1530 | 1530 | 100% | 0.0 | 99.29% | NC_045179.1 | *B.terrestris* |
| Drӑgusani | 46.29929 | 26.97973 | 2017_Bombus_119 | 10.05.2017 | MT174859 | 1539 | 1539 | 100% | 0.0 | 98.73% | AY181170.1 | *B.terrestris* |
| Drӑgusani | 46.29929 | 26.97973 | 2017_Bombus_120 | 10.05.2017 | MT174860 | 1609 | 1609 | 100% | 0.0 | 99.33% | AY181170.1 | *B.terrestris* |
| Drӑgusani | 46.29929 | 26.97973 | 2017_Bombus_121 | 10.05.2017 | MT174861 | 1140 | 1140 | 100% | 0.0 | 98.31% | NC_045179.1 | *B.terrestris* |
| Drӑgusani | 46.29929 | 26.97973 | 2017_Bombus_122 | 10.05.2017 | MT174862 | 1212 | 1212 | 99% | 0.0 | 99.55% | NC_045179.1 | *B.terrestris* |
| Drӑgusani | 46.29929 | 26.97973 | 2017_Bombus_123 | 10.05.2017 | MT174863 | 1393 | 1393 | 99% | 0.0 | 98.73% | KT164681.1 | *B.lucorum* |
| Drӑgusani | 46.29929 | 26.97973 | 2017_Bombus_124 | 10.05.2017 | MT174864 | 1764 | 1764 | 99% | 0.0 | 98.70% | NC_045179.1 | *B.terrestris* |
| Drӑgusani | 46.29929 | 26.97973 | 2017_Bombus_125 | 10.05.2017 | MT174865 | 1622 | 1622 | 99% | 0.0 | 96.94% | NC_045179.1 | *B.terrestris* |
| Drӑgusani | 46.29929 | 26.97973 | 2017_Bombus_126 | 10.05.2017 | MT174866 | 1831 | 1831 | 99% | 0.0 | 99.41% | NC_045179.1 | *B.terrestris* |
| Drӑgusani | 46.29929 | 26.97973 | 2017_Bombus_127 | 10.05.2017 | MT174867 | 1714 | 1714 | 99% | 0.0 | 97.35% | NC_045179.1 | *B.terrestris* |
|  |  |  |  |  |  |  |  |  |  |  |  |  |
| Föen | 45.51085 | 20.87627 | 2017_Bombus_342 | 03.06.2017 | MT175077 | 1722 | 1722 | 99% | 0.0 | 99.79% | NC_045179.1 | *B.terrestris* |
| Föen | 45.51085 | 20.87627 | 2017_Bombus_343 | 03.06.2017 | MT175078 | 1070 | 1070 | 100% | 0.0 | 99.83% | NC_045179.1 | *B.terrestris* |
| Föen | 45.51085 | 20.87627 | 2017_Bombus_344 | 03.06.2017 | MT175079 | 1334 | 1334 | 100% | 0.0 | 99.73% | AY181170.1 | *B.terrestris* |
| Föen | 45.51085 | 20.87627 | 2017_Bombus_345 | 03.06.2017 | MT175080 | 1602 | 1602 | 100% | 0.0 | 99.43% | NC_045179.1 | *B.terrestris* |
| Föen | 45.51085 | 20.87627 | 2017_Bombus_346 | 03.06.2017 | MT175081 | 985 | 985 | 100% | 0.0 | 100.00% | NC_045179.1 | *B.terrestris* |
| Föen | 45.51085 | 20.87627 | 2017_Bombus_347 | 03.06.2017 | MT175082 | 1472 | 1472 | 100% | 0.0 | 99.63% | NC_045179.1 | *B.terrestris* |
| Föen | 45.51085 | 20.87627 | 2017_Bombus_348 | 03.06.2017 | MT175083 | 1506 | 1506 | 99% | 0.0 | 96.72% | NC_045179.1 | *B.terrestris* |
| Föen | 45.51085 | 20.87627 | 2017_Bombus_349 | 03.06.2017 | MT175084 | 1829 | 1829 | 100% | 0.0 | 99.90% | NC_045179.1 | *B.terrestris* |
| Föen | 45.51085 | 20.87627 | 2017_Bombus_350 | 03.06.2017 | MT175085 | 1155 | 1155 | 99% | 0.0 | 100.00% | NC_045179.1 | *B.terrestris* |
| Föen | 45.51085 | 20.87627 | 2017_Bombus_351 | 03.06.2017 | MT175086 | 1282 | 1282 | 100% | 0.0 | 99.86% | NC_045179.1 | *B.terrestris* |
| Föen | 45.51085 | 20.87627 | 2017_Bombus_352 | 03.06.2017 | MT175087 | 1790 | 1790 | 100% | 0.0 | 98.91% | NC_045179.1 | *B.terrestris* |
| Föen | 45.51085 | 20.87627 | 2017_Bombus_353 | 03.06.2017 | MT175088 | 1851 | 1851 | 99% | 0.0 | 99.90% | NC_045179.1 | *B.terrestris* |
| Föen | 45.51085 | 20.87627 | 2017_Bombus_354 | 03.06.2017 | MT175089 | 1781 | 1781 | 100% | 0.0 | 99.59% | NC_045179.1 | *B.terrestris* |
| Föen | 45.51085 | 20.87627 | 2017_Bombus_355 | 03.06.2017 | MT175090 | 1391 | 1391 | 100% | 0.0 | 98.73% | AY181170.1 | *B.terrestris* |
| Föen | 45.51085 | 20.87627 | 2017_Bombus_356 | 03.06.2017 | MT175091 | 1722 | 1722 | 99% | 0.0 | 98.57% | NC_045179.1 | *B.terrestris* |
| Föen | 45.51085 | 20.87627 | 2017_Bombus_357 | 03.06.2017 | MT175092 | 1500 | 1500 | 99% | 0.0 | 98.93% | AY530009.1 | *B.lucorum* |
| Föen | 45.51085 | 20.87627 | 2017_Bombus_358 | 03.06.2017 | MT175093 | 1738 | 1738 | 100% | 0.0 | 98.87% | NC_045179.1 | *B.terrestris* |
| Föen | 45.51085 | 20.87627 | 2017_Bombus_359 | 03.06.2017 | MT175094 | 1430 | 1430 | 100% | 0.0 | 98.41% | NC_045179.1 | *B.terrestris* |
| Föen | 45.51085 | 20.87627 | 2017_Bombus_360 | 03.06.2017 | MT175095 | 1504 | 1504 | 99% | 0.0 | 98.38% | NC_045179.1 | *B.terrestris* |
| Föen | 45.51085 | 20.87627 | 2017_Bombus_361 | 03.06.2017 | MT175096 | 1434 | 1434 | 99% | 0.0 | 96.59% | NC_045179.1 | *B.terrestris* |
| Föen | 45.51085 | 20.87627 | 2017_Bombus_362 | 03.06.2017 | MT175097 | 1731 | 1731 | 100% | 0.0 | 99.48% | NC_045179.1 | *B.terrestris* |
| Föen | 45.51085 | 20.87627 | 2017_Bombus_363 | 03.06.2017 | MT175098 | 1500 | 1500 | 99% | 0.0 | 96.34% | AY181170.1 | *B.terrestris* |
| Föen | 45.51085 | 20.87627 | 2017_Bombus_364 | 03.06.2017 | MT175099 | 1515 | 1515 | 99% | 0.0 | 96.85% | NC_045179.1 | *B.terrestris* |
| Föen | 45.51085 | 20.87627 | 2017_Bombus_365 | 03.06.2017 | MT175100 | 1683 | 1683 | 100% | 0.0 | 99.89% | NC_045179.1 | *B.terrestris* |
| Föen | 45.51085 | 20.87627 | 2017_Bombus_366 | 03.06.2017 | MT175101 | 1701 | 1701 | 100% | 0.0 | 100.00% | AY181170.1 | *B.terrestris* |
| Föen | 45.51085 | 20.87627 | 2017_Bombus_367 | 03.06.2017 | MT175102 | 929 | 929 | 100% | 0.0 | 100.00% | NC_045179.1 | *B.terrestris* |
| Föen | 45.51085 | 20.87627 | 2017_Bombus_368 | 03.06.2017 | MT175103 | 1744 | 1744 | 99% | 0.0 | 99.08% | NC_045179.1 | *B.terrestris* |
| Föen | 45.51085 | 20.87627 | 2017_Bombus_369 | 03.06.2017 | MT175104 | 1554 | 1554 | 99% | 0.0 | 96.92% | NC_045179.1 | *B.terrestris* |
| Föen | 45.51085 | 20.87627 | 2017_Bombus_370 | 03.06.2017 | MT175105 | 1371 | 1371 | 100% | 0.0 | 99.21% | NC_045179.1 | *B.terrestris* |
| Föen | 45.51085 | 20.87627 | 2017_Bombus_371 | 03.06.2017 | MT175106 | 1430 | 1430 | 100% | 0.0 | 96.29% | NC_045179.1 | *B.terrestris* |
| Föen | 45.51085 | 20.87627 | 2017_Bombus_372 | 03.06.2017 | MT175107 | 1227 | 1227 | 100% | 0.0 | 98.43% | NC_045179.1 | *B.terrestris* |
| Föen | 45.51085 | 20.87627 | 2017_Bombus_373 | 03.06.2017 | MT175108 | 1437 | 1437 | 99% | 0.0 | 97.75% | AY181170.1 | *B.terrestris* |
|  |  |  |  |  |  |  |  |  |  |  |  |  |
| Golitsa | 42.90956 | 27.52514 | 2013_Bt_0127 | 28.05.2013 | MT174696 | 1825 | 1825 | 99% | 0.0 | 99.70% | NC_045179.1 | *B.terrestris* |
| Golitsa | 42.90956 | 27.52514 | 2013_Bt_0128 | 28.05.2013 | MT174697 | 1869 | 1869 | 100% | 0.0 | 99.71% | NC_045179.1 | *B.terrestris* |
| Golitsa | 42.90956 | 27.52514 | 2013_Bt_0129 | 28.05.2013 | MT174698 | 1858 | 1858 | 99% | 0.0 | 99.70% | NC_045179.1 | *B.terrestris* |
| Golitsa | 42.90956 | 27.52514 | 2013_Bt_0130 | 28.05.2013 | MT174699 | 1862 | 1862 | 99% | 0.0 | 99.71% | NC_045179.1 | *B.terrestris* |
| Golitsa | 42.90956 | 27.52514 | 2013_Bt_0131 | 28.05.2013 | MT174700 | 1866 | 1866 | 99% | 0.0 | 99.61% | NC_045179.1 | *B.terrestris* |
| Golitsa | 42.90956 | 27.52514 | 2013_Bt_0132 | 28.05.2013 | MT174701 | 1853 | 1853 | 99% | 0.0 | 99.70% | NC_045179.1 | *B.terrestris* |
| Golitsa | 42.90956 | 27.52514 | 2013_Bt_0133 | 28.05.2013 | MT174702 | 1184 | 1184 | 99% | 0.0 | 99.69% | NC_045179.1 | *B.terrestris* |
| Golitsa | 42.90956 | 27.52514 | 2013_Bt_0134 | 28.05.2013 | MT174703 | 1862 | 1862 | 99% | 0.0 | 99.71% | NC_045179.1 | *B.terrestris* |
| Golitsa | 42.90956 | 27.52514 | 2013_Bt_0135 | 28.05.2013 | MT174704 | 1864 | 1864 | 100% | 0.0 | 99.51% | NC_045179.1 | *B.terrestris* |
| Golitsa | 42.90956 | 27.52514 | 2013_Bt_0136 | 28.05.2013 | MT174705 | 1853 | 1853 | 99% | 0.0 | 99.70% | NC_045179.1 | *B.terrestris* |
| Golitsa | 42.90956 | 27.52514 | 2013_Bt_0137 | 28.05.2013 | MT174706 | 1881 | 1881 | 99% | 0.0 | 99.80% | NC_045179.1 | *B.terrestris* |
| Golitsa | 42.90956 | 27.52514 | 2013_Bt_0138 | 28.05.2013 | MT174707 | 1866 | 1866 | 100% | 0.0 | 99.71% | NC_045179. | *B.terrestris* |
| Golitsa | 42.90956 | 27.52514 | 2013_Bt_0139 | 28.05.2013 | MT174708 | 1881 | 1881 | 100% | 0.0 | 99.80% | NC_045179.1 | *B.terrestris* |
| Gothal | 45.40790 | 21.42069 | 2017_Bombus_313 | 03.06.2017 | MT175048 | 1799 | 1799 | 100% | 0.0 | 99.59% | NC_045179.1 | *B.terrestris* |
| Gothal | 45.40790 | 21.42069 | 2017_Bombus_314 | 03.06.2017 | MT175049 | 1709 | 1709 | 100% | 0.0 | 99.06% | NC_045179.1 | *B.terrestris* |
| Gothal | 45.40790 | 21.42069 | 2017_Bombus_315 | 03.06.2017 | MT175050 | 1668 | 1668 | 100% | 0.0 | 98.83% | AY181170.1 | *B.terrestris* |
| Gothal | 45.40790 | 21.42069 | 2017_Bombus_316 | 03.06.2017 | MT175051 | 1807 | 1807 | 99% | 0.0 | 99.20% | NC_045179.1 | *B.terrestris* |
| Gothal | 45.40790 | 21.42069 | 2017_Bombus_317 | 03.06.2017 | MT175052 | 1746 | 1746 | 100% | 0.0 | 99.17% | NC_045179.1 | *B.terrestris* |
| Gothal | 45.40790 | 21.42069 | 2017_Bombus_318 | 03.06.2017 | MT175053 | 1406 | 1406 | 100% | 0.0 | 99.48% | NC_045179.1 | *B.terrestris* |
| Gothal | 45.40790 | 21.42069 | 2017_Bombus_319 | 03.06.2017 | MT175054 | 1823 | 1823 | 100% | 0.0 | 99.50% | NC_045179.1 | *B.terrestris* |
| Gothal | 45.40790 | 21.42069 | 2017_Bombus_320 | 03.06.2017 | MT175055 | 1411 | 1411 | 100% | 0.0 | 99.74% | NC_045179.1 | *B.terrestris* |
| Gothal | 45.40790 | 21.42069 | 2017_Bombus_321 | 03.06.2017 | MT175056 | 1816 | 1816 | 100% | 0.0 | 99.90% | AY181170.1 | *B.terrestris* |
| Gothal | 45.40790 | 21.42069 | 2017_Bombus_322 | 03.06.2017 | MT175057 | 1666 | 1666 | 100% | 0.0 | 98.42% | NC_045179.1 | *B.terrestris* |
| Gothal | 45.40790 | 21.42069 | 2017_Bombus_323 | 03.06.2017 | MT175058 | 1677 | 1677 | 100% | 0.0 | 98.14% | AY181170.1 | *B.terrestris* |
| Gothal | 45.40790 | 21.42069 | 2017_Bombus_324 | 03.06.2017 | MT175059 | 1639 | 1639 | 100% | 0.0 | 99.78% | AY181170.1 | *B.terrestris* |
| Gothal | 45.40790 | 21.42069 | 2017_Bombus_325 | 03.06.2017 | MT175060 | 1818 | 1818 | 100% | 0.0 | 99.60% | NC_045179.1 | *B.terrestris* |
| Gothal | 45.40790 | 21.42069 | 2017_Bombus_326 | 03.06.2017 | MT175061 | 1871 | 1871 | 99% | 0.0 | 100.00% | NC_045179.1 | *B.terrestris* |
| Gothal | 45.40790 | 21.42069 | 2017_Bombus_327 | 03.06.2017 | MT175062 | 1491 | 1491 | 100% | 0.0 | 100.00% | NC_045179.1 | *B.terrestris* |
| Gothal | 45.40790 | 21.42069 | 2017_Bombus_328 | 03.06.2017 | MT175063 | 1773 | 1773 | 100% | 0.0 | 99.79% | NC_045179.1 | *B.terrestris* |
| Gothal | 45.40790 | 21.42069 | 2017_Bombus_329 | 03.06.2017 | MT175064 | 1552 | 1552 | 100% | 0.0 | 98.85% | AY181170.1 | *B.terrestris* |
| Gothal | 45.40790 | 21.42069 | 2017_Bombus_330 | 03.06.2017 | MT175065 | 1526 | 1526 | 100% | 0.0 | 99.41% | AY181170.1 | *B.terrestris* |
| Gothal | 45.40790 | 21.42069 | 2017_Bombus_331 | 03.06.2017 | MT175066 | 1731 | 1731 | 99% | 0.0 | 99.48% | NC_045179.1 | *B.terrestris* |
| Gothal | 45.40790 | 21.42069 | 2017_Bombus_332 | 03.06.2017 | MT175067 | 1712 | 1712 | 100% | 0.0 | 98.76% | AY181170.1 | *B.terrestris* |
| Gothal | 45.40790 | 21.42069 | 2017_Bombus_333 | 03.06.2017 | MT175068 | 1493 | 1493 | 100% | 0.0 | 98.14% | AY181170.1 | *B.terrestris* |
| Gothal | 45.40790 | 21.42069 | 2017_Bombus_334 | 03.06.2017 | MT175069 | 1526 | 1526 | 100% | 0.0 | 99.88% | AY181170.1 | *B.terrestris* |
| Gothal | 45.40790 | 21.42069 | 2017_Bombus_335 | 03.06.2017 | MT175070 | 1805 | 1805 | 99% | 0.0 | 99.70% | NC_045179.1 | *B.terrestris* |
| Gothal | 45.40790 | 21.42069 | 2017_Bombus_336 | 03.06.2017 | MT175071 | 1266 | 1266 | 100% | 0.0 | 99.71% | AY181170.1 | *B.terrestris* |
| Gothal | 45.40790 | 21.42069 | 2017_Bombus_337 | 03.06.2017 | MT175072 | 1666 | 1666 | 99% | 0.0 | 99.03% | AY181170.1 | *B.terrestris* |
| Gothal | 45.40790 | 21.42069 | 2017_Bombus_338 | 03.06.2017 | MT175073 | 1618 | 1618 | 100% | 0.0 | 99.89% | AY181170.1 | *B.terrestris* |
| Gothal | 45.40790 | 21.42069 | 2017_Bombus_339 | 03.06.2017 | MT175074 | 1812 | 1812 | 100% | 0.0 | 99.60% | NC_045179.1 | *B.terrestris* |
| Gothal | 45.40790 | 21.42069 | 2017_Bombus_340 | 03.06.2017 | MT175075 | 1568 | 1568 | 100% | 0.0 | 98.97% | AY181170.1 | *B.terrestris* |
| Gothal | 45.40790 | 21.42069 | 2017_Bombus_341 | 03.06.2017 | MT175076 | 1349 | 1349 | 100% | 0.0 | 99.73% | NC_045179.1 | *B.terrestris* |
|  |  |  |  |  |  |  |  |  |  |  |  |  |
| Grohotno | 41.70118 | 24.38684 | 2017_Bombus_205 | 31.05.2017 | MT174945 | 1869 | 1869 | 100% | 0.0 | 99.61% | KT164681.1 | *B.lucorum* |
| Grohotno | 41.70118 | 24.38684 | 2017_Bombus_206 | 31.05.2017 | MT174946 | 1866 | 1866 | 100% | 0.0 | 99.90% | NC_045179.1 | *B.terrestris* |
| Grohotno | 41.70118 | 24.38684 | 2017_Bombus_207 | 31.05.2017 | MT174947 | 1866 | 1866 | 100% | 0.0 | 100.00% | KT164681.1 | *B.lucorum* |
| Grohotno | 41.70118 | 24.38684 | 2017_Bombus_208 | 31.05.2017 | MT174948 | 1860 | 1860 | 100% | 0.0 | 99.90% | KT164681.1 | *B.lucorum* |
| Grohotno | 41.70118 | 24.38684 | 2017_Bombus_209 | 31.05.2017 | MT174949 | 1855 | 1855 | 100% | 0.0 | 99.90% | KT164681.1 | *B.lucorum* |
| Grohotno | 41.70118 | 24.38684 | 2017_Bombus_210 | 31.05.2017 | MT174950 | 1847 | 1847 | 100% | 0.0 | 99.90% | KT164681.1 | *B.lucorum* |
| Grohotno | 41.70118 | 24.38684 | 2017_Bombus_211 | 31.05.2017 | MT174951 | 1818 | 1818 | 100% | 0.0 | 99.70% | NC_045179.1 | *B.terrestris* |
| Grohotno | 41.70118 | 24.38684 | 2017_Bombus_212 | 31.05.2017 | MT174952 | 1642 | 1642 | 100% | 0.0 | 96.87% | NC_045179.1 | *B.terrestris* |
| Grohotno | 41.70118 | 24.38684 | 2017_Bombus_213 | 31.05.2017 | MT174953 | 1884 | 1884 | 100% | 0.0 | 99.90% | KT164681.1 | *B.lucorum* |
| Grohotno | 41.70118 | 24.38684 | 2017_Bombus_214 | 31.05.2017 | MT174954 | 1580 | 1580 | 99% | 0.0 | 98.66% | KT164681.1 | *B.lucorum* |
| Grohotno | 41.70118 | 24.38684 | 2017_Bombus_215 | 31.05.2017 | MT174955 | 1847 | 1847 | 100% | 0.0 | 99.90% | NC_045179.1 | *B.terrestris* |
| Grohotno | 41.70118 | 24.38684 | 2017_Bombus_216 | 31.05.2017 | MT174956 | 1864 | 1864 | 99% | 0.0 | 99.80% | KT164681.1 | *B.lucorum* |
| Grohotno | 41.70118 | 24.38684 | 2017_Bombus_217 | 31.05.2017 | MT174957 | 1881 | 1881 | 100% | 0.0 | 99.90% | KT164681.1 | *B.lucorum* |
| Grohotno | 41.70118 | 24.38684 | 2017_Bombus_218 | 31.05.2017 | MT174958 | 1853 | 1853 | 100% | 0.0 | 100.00% | KT164681.1 | *B.lucorum* |
| Grohotno | 41.70118 | 24.38684 | 2017_Bombus_219 | 31.05.2017 | MT174959 | 1866 | 1866 | 100% | 0.0 | 99.80% | NC_045179.1 | *B.terrestris* |
| Grohotno | 41.70118 | 24.38684 | 2017_Bombus_220 | 31.05.2017 | MT174960 | 1858 | 1858 | 99% | 0.0 | 100.00% | KT164681.1 | *B.terrestris* |
| Grohotno | 41.70118 | 24.38684 | 2017_Bombus_221 | 31.05.2017 | MT174961 | 1853 | 1853 | 100% | 0.0 | 100.00% | KT164681.1 | *B.lucorum* |
| Grohotno | 41.70118 | 24.38684 | 2017_Bombus_222 | 31.05.2017 | MT174962 | 1858 | 1858 | 99% | 0.0 | 99.80% | KT164681.1 | *B.lucorum* |
| Grohotno | 41.70118 | 24.38684 | 2017_Bombus_223 | 31.05.2017 | MT174963 | 1858 | 1858 | 99% | 0.0 | 99.90% | KT164681.1 | *B.lucorum* |
| Grohotno | 41.70118 | 24.38684 | 2017_Bombus_224 | 31.05.2017 | MT174964 | 1853 | 1853 | 100% | 0.0 | 99.51% | NC_045179.1 | *B.terrestris* |
| Grohotno | 41.70118 | 24.38684 | 2017_Bombus_225 | 31.05.2017 | MT174965 | 1862 | 1862 | 100% | 0.0 | 99.51% | KT164681.1 | *B.lucorum* |
| Grohotno | 41.70118 | 24.38684 | 2017_Bombus_226 | 31.05.2017 | MT174966 | 1875 | 1875 | 99% | 0.0 | 99.80% | NC_045179.1 | *B.terrestris* |
| Grohotno | 41.70118 | 24.38684 | 2017_Bombus_227 | 31.05.2017 | MT174967 | 1855 | 1855 | 100% | 0.0 | 99.90% | KT164681.1 | *B.lucorum* |
| Grohotno | 41.70118 | 24.38684 | 2017_Bombus_228 | 31.05.2017 | MT174968 | 1869 | 1869 | 100% | 0.0 | 99.90% | KT164681.1 | *B.lucorum* |
| Grohotno | 41.70118 | 24.38684 | 2017_Bombus_229 | 31.05.2017 | MT174969 | 1845 | 1845 | 100% | 0.0 | 99.31% | KT164681.1 | *B.lucorum* |
| Grohotno | 41.70118 | 24.38684 | 2017_Bombus_230 | 31.05.2017 | MT174970 | 1845 | 1845 | 100% | 0.0 | 99.60% | NC_045179.1 | *B.terrestris* |
| Grohotno | 41.70118 | 24.38684 | 2017_Bombus_231 | 31.05.2017 | MT174971 | 1801 | 1801 | 99% | 0.0 | 98.81% | KT164681.1 | *B.lucorum* |
| Grohotno | 41.70118 | 24.38684 | 2017_Bombus_232 | 31.05.2017 | MT174972 | 1746 | 1746 | 100% | 0.0 | 99.48% | KT164681.1 | *B.lucorum* |
| Grohotno | 41.70118 | 24.38684 | 2017_Bombus_233 | 31.05.2017 | MT174973 | 1820 | 1820 | 100% | 0.0 | 99.50% | NC_045179.1 | *B.terrestris* |
| Grohotno | 41.70118 | 24.38684 | 2017_Bombus_234 | 31.05.2017 | MT174974 | 1844 | 1844 | 100% | 0.0 | 99.60% | KT164681.1 | *B.lucorum* |
| Grohotno | 41.70118 | 24.38684 | 2017_Bombus_235 | 31.05.2017 | MT174975 | 1842 | 1842 | 100% | 0.0 | 99.90% | KT164681.1 | *B.lucorum* |
| Grohotno | 41.70118 | 24.38684 | 2017_Bombus_236 | 31.05.2017 | MT174976 | 1849 | 1849 | 100% | 0.0 | 100.00% | KT164681.1 | *B.lucorum* |
| Grohotno | 41.70118 | 24.38684 | 2017_Bombus_237 | 31.05.2017 | MT174977 | 1851 | 1851 | 100% | 0.0 | 99.80% | KT164681.1 | *B.lucorum* |
| Grohotno | 41.70118 | 24.38684 | 2017_Bombus_238 | 31.05.2017 | MT174978 | 1777 | 1777 | 100% | 0.0 | 99.79% | KT164681.1 | *B.lucorum* |
| Grohotno | 41.70118 | 24.38684 | 2017_Bombus_239 | 31.05.2017 | MT174979 | 1480 | 1480 | 100% | 0.0 | 99.88% | KT164681.1 | *B.lucorum* |
|  |  |  |  |  |  |  |  |  |  |  |  |  |
| Gura Glodului | 47.13575 | 25.50107 | 2017_Bombus_001 | 04.05.2017 | MT174751 | 1445 | 1445 | 100% | 0.0 | 99.62% | AY181119.1 | *B.lucorum* |
| Gura Glodului | 47.13575 | 25.50107 | 2017_Bombus_002 | 04.05.2017 | MT174752 | 1847 | 1847 | 100% | 0.0 | 99.80% | KT164681.1 | *B.lucorum* |
| Gura Glodului | 47.13575 | 25.50107 | 2017_Bombus_003 | 04.05.2017 | MT174753 | 1768 | 1768 | 99% | 0.0 | 98.51% | KT164681.1 | *B.lucorum* |
| Gura Glodului | 47.13575 | 25.50107 | 2017_Bombus_004 | 04.05.2017 | MT174754 | 1772 | 1772 | 100% | 0.0 | 99.19% | KT164681. | *B.lucorum* |
| Gura Glodului | 47.13575 | 25.50107 | 2017_Bombus_005 | 04.05.2017 | MT174755 | 1836 | 1836 | 100% | 0.0 | 99.41% | KT164681.1 | *B.lucorum* |
| Gura Glodului | 47.13575 | 25.50107 | 2017_Bombus_006 | 04.05.2017 | MT174756 | 1794 | 1794 | 99% | 0.0 | 98.72% | KT164681.1 | *B.lucorum* |
| Gura Glodului | 47.13575 | 25.50107 | 2017_Bombus_007 | 04.05.2017 | MT174757 | 1842 | 1842 | 99% | 0.0 | 99.80% | KT164681.1 | *B.lucorum* |
| Gura Glodului | 47.13575 | 25.50107 | 2017_Bombus_008 | 04.05.2017 | MT174758 | 654 | 654 | 91% | 0.0 | 80.17% | KT164681.1 | *B.terrestris* |
| Gura Glodului | 47.13575 | 25.50107 | 2017_Bombus_009 | 04.05.2017 | MT174759 | 1812 | 1812 | 100% | 0.0 | 99.30% | KT164681.1 | *B.lucorum* |
| Gura Glodului | 47.13575 | 25.50107 | 2017_Bombus_011 | 04.05.2017 | MT174760 | 1762 | 1762 | 100% | 0.0 | 99.08% | KT164681.1 | *B.lucorum* |
| Gura Glodului | 47.13575 | 25.50107 | 2017_Bombus_012 | 04.05.2017 | MT174761 | 1650 | 1650 | 100% | 0.0 | 97.71% | KT164681.1 | *B.lucorum* |
| Gura Glodului | 47.13575 | 25.50107 | 2017_Bombus_014 | 04.05.2017 | MT174762 | 1790 | 1790 | 99% | 0.0 | 99.59% | KT164681.1 | *B.lucorum* |
| Gura Glodului | 47.13575 | 25.50107 | 2017_Bombus_015 | 04.05.2017 | MT174763 | 1059 | 1059 | 99% | 0.0 | 96.29% | KT164681.1 | *B.lucorum* |
| Gura Glodului | 47.13575 | 25.50107 | 2017_Bombus_017 | 04.05.2017 | MT174764 | 1823 | 1823 | 99% | 0.0 | 99.60% | KT164681.1 | *B.lucorum* |
| Gura Glodului | 47.13575 | 25.50107 | 2017_Bombus_018 | 04.05.2017 | MT174765 | 1862 | 1862 | 100% | 0.0 | 99.42% | KT164681. | *B.lucorum* |
| Gura Glodului | 47.13575 | 25.50107 | 2017_Bombus_019 | 04.05.2017 | MT174766 | 1844 | 1844 | 100% | 0.0 | 99.41% | NC_045179.1 | *B.terrestris* |
| Gura Glodului | 47.13575 | 25.50107 | 2017_Bombus_020 | 04.05.2017 | MT174767 | 1860 | 1860 | 99% | 0.0 | 100.00% | KT16468 | *B.lucorum* |
| Gura Glodului | 47.13575 | 25.50107 | 2017_Bombus_021 | 04.05.2017 | MT174768 | 1838 | 1838 | 99% | 0.0 | 99.70% | KT164681.1 | *B.lucorum* |
| Gura Glodului | 47.13575 | 25.50107 | 2017_Bombus_022 | 04.05.2017 | MT174769 | 1844 | 1844 | 100% | 0.0 | 99.51% | KT164681.1 | *B.lucorum* |
| Gura Glodului | 47.13575 | 25.50107 | 2017_Bombus_023 | 04.05.2017 | MT174770 | 1779 | 1779 | 100% | 0.0 | 99.79% | KT164681.1 | *B.lucorum* |
| Gura Glodului | 47.13575 | 25.50107 | 2017_Bombus_024 | 04.05.2017 | MT174771 | 1831 | 1831 | 100% | 0.0 | 99.02% | KT164681.1 | *B.lucorum* |
| Gura Glodului | 47.13575 | 25.50107 | 2017_Bombus_025 | 04.05.2017 | MT174772 | 1836 | 1836 | 100% | 0.0 | 99.80% | KT164681.1 | *B.lucorum* |
|  |  |  |  |  |  |  |  |  |  |  |  |  |
| Gura Haitii | 47.17505 | 25.25018 | 2013_Bt_0247 | 20.07.2013 | MT175175 | 1748 | 1748 | 100% | 0.0 | 98.12% | KT164681.1 | *B.lucorum* |
| Gura Haitii | 47.17505 | 25.25018 | 2013_Bt_0248 | 20.07.2013 | MT175176 | 1783 | 1783 | 100% | 0.0 | 98.80% | KT164681.1 | *B.lucorum* |
| Gura Haitii | 47.17505 | 25.25018 | 2013_Bt_0249 | 20.07.2013 | MT175177 | 1722 | 1722 | 97% | 0.0 | 98.86% | KT164681.1 | *B.lucorum* |
| Gura Haitii | 47.17505 | 25.25018 | 2013_Bt_0252 | 20.07.2013 | MT175178 | 1731 | 1731 | 99% | 0.0 | 98.77% | KT164681.1 | *B.lucorum* |
| Gura Haitii | 47.17505 | 25.25018 | 2013_Bt_0253 | 20.07.2013 | MT175179 | 1794 | 1794 | 100% | 0.0 | 99.10% | KT164681.1 | *B.lucorum* |
| Gura Haitii | 47.17505 | 25.25018 | 2013_Bt_0254 | 20.07.2013 | MT175180 | 1591 | 1591 | 100% | 0.0 | 98.56% | KT164681.1 | *B.lucorum* |
| Gura Haitii | 47.17505 | 25.25018 | 2013_Bt_0255 | 20.07.2013 | MT175181 | 1731 | 1731 | 100% | 0.0 | 97.64% | KT164681.1 | *B.lucorum* |
| Gura Haitii | 47.17505 | 25.25018 | 2013_Bt_0256 | 20.07.2013 | MT175182 | 1827 | 1827 | 100% | 0.0 | 99.31% | KT164681.1 | *B.lucorum* |
| Gura Haitii | 47.17505 | 25.25018 | 2013_Bt_0257 | 20.07.2013 | MT175183 | 1808 | 1808 | 99% | 0.0 | 98.92% | KT164681.1 | *B.lucorum* |
| Gura Haitii | 47.17505 | 25.25018 | 2013_Bt_0258 | 20.07.2013 | MT175184 | 1550 | 1550 | 100% | 0.0 | 98.10% | KT164681.1 | *B.lucorum* |
| Gura Haitii | 47.17505 | 25.25018 | 2013_Bt_0259 | 20.07.2013 | MT175185 | 1653 | 1653 | 98% | 0.0 | 97.51% | KT164681.1 | *B.lucorum* |
| Gura Haitii | 47.17505 | 25.25018 | 2013_Bt_0260 | 20.07.2013 | MT175186 | 1685 | 1685 | 98% | 0.0 | 98.05% | KT164681.1 | *B.lucorum* |
| Gura Haitii | 47.17505 | 25.25018 | 2013_Bt_0261 | 20.07.2013 | MT175187 | 1507 | 1507 | 99% | 0.0 | 98.94% | KT164681.1 | *B.lucorum* |
| Gura Haitii | 47.17505 | 25.25018 | 2013_Bt_0262 | 20.07.2013 | MT175188 | 1733 | 1733 | 100% | 0.0 | 98.76% | KT164681.1 | *B.lucorum* |
| Gura Haitii | 47.17505 | 25.25018 | 2013_Bt_0263 | 20.07.2013 | MT175189 | 747 | 747 | 100% | 0.0 | 98.58% | KT164681.1 | *B.lucorum* |
|  |  |  |  |  |  |  |  |  |  |  |  |  |
| Handal | 47.65028 | 23.89441 | 2013_Bt_0216 | 18.07.2013 | MT175154 | 1013 | 1013 | 100% | 0.0 | 98.44% | KT164681.1 | *B.lucorum* |
| Handal | 47.65028 | 23.89441 | 2013_Bt_0217 | 18.07.2013 | MT175155 | 542 | 542 | 100% | 4e-150 | 98.69% | KT164681.1 | *B.lucorum* |
| Handal | 47.65028 | 23.89441 | 2013_Bt_0218 | 18.07.2013 | MT175156 | 1277 | 1277 | 100% | 0.0 | 99.71% | KT164681.1 | *B.lucorum* |
| Handal | 47.65028 | 23.89441 | 2013_Bt_0220 | 18.07.2013 | MT175157 | 1038 | 1038 | 100% | 0.0 | 99.82% | KT164681.1 | *B.lucorum* |
| Handal | 47.65028 | 23.89441 | 2013_Bt_0221 | 18.07.2013 | MT175158 | 1264 | 1264 | 100% | 0.0 | 99.01% | KT164681.1 | *B.lucorum* |
| Handal | 47.65028 | 23.89441 | 2013_Bt_0222 | 18.07.2013 | MT175159 | 568 | 568 | 100% | 7e-158 | 100.00% | KT164681.1 | *B.lucorum* |
| Handal | 47.65028 | 23.89441 | 2013_Bt_0223 | 18.07.2013 | MT175160 | 1290 | 1290 | 100% | 0.0 | 99.16% | KT164681.1 | *B.lucorum* |
| Handal | 47.65028 | 23.89441 | 2013_Bt_0224 | 18.07.2013 | MT175161 | 732 | 732 | 99% | 0.0 | 99.75% | KT164681.1 | *B.lucorum* |
| Handal | 47.65028 | 23.89441 | 2013_Bt_0225 | 18.07.2013 | MT175162 | 1328 | 1328 | 99% | 0.0 | 97.93% | KT164681.1 | *B.lucorum* |
| Handal | 47.65028 | 23.89441 | 2013_Bt_0226 | 18.07.2013 | MT175163 | 896 | 896 | 100% | 0.0 | 98.80% | KT164681.1 | *B.lucorum* |
| Handal | 47.65028 | 23.89441 | 2013_Bt_0227 | 18.07.2013 | MT175164 | 305 | 305 | 97% | 3e-79 | 96.72% | KT164681.1 | *B.lucorum* |
| Handal | 47.65028 | 23.89441 | 2013_Bt_0228 | 18.07.2013 | MT175165 | 505 | 505 | 100% | 5e-139 | 97.63% | KT164681.1 | *B.lucorum* |
| Handal | 47.65028 | 23.89441 | 2013_Bt_0229 | 18.07.2013 | MT175166 | 1513 | 1513 | 99% | 0.0 | 98.16% | KT164681.1 | *B.lucorum* |
| Handal | 47.65028 | 23.89441 | 2013_Bt_0230 | 18.07.2013 | MT175167 | 926 | 926 | 100% | 0.0 | 99.61% | KT164681.1 | *B.lucorum* |
| Handal | 47.65028 | 23.89441 | 2013_Bt_0233 | 18.07.2013 | MT175168 | 1186 | 1186 | 100% | 0.0 | 99.24% | KT164681.1 | *B.lucorum* |
| Handal | 47.65028 | 23.89441 | 2013_Bt_0234 | 18.07.2013 | MT175169 | 845 | 845 | 100% | 0.0 | 99.57% | KT164681.1 | *B.lucorum* |
| Handal | 47.65028 | 23.89441 | 2013_Bt_0235 | 18.07.2013 | MT175170 | 1430 | 1430 | 100% | 0.0 | 99.00% | KT164681.1 | *B.lucorum* |
| Handal | 47.65028 | 23.89441 | 2013_Bt_0236 | 18.07.2013 | MT175171 | 1417 | 1417 | 100% | 0.0 | 99.11% | KT164681.1 | *B.lucorum* |
| Handal | 47.65028 | 23.89441 | 2013_Bt_0237 | 18.07.2013 | MT175172 | 837 | 837 | 100% | 0.0 | 99.14% | KT164681.1 | *B.lucorum* |
| Handal | 47.65028 | 23.89441 | 2013_Bt_0238 | 18.07.2013 | MT175173 | 1554 | 1554 | 100% | 0.0 | 99.08% | KT164681.1 | *B.lucorum* |
| Handal | 47.65028 | 23.89441 | 2013_Bt_0239 | 18.07.2013 | MT175174 | 1062 | 1062 | 100% | 0.0 | 98.99% | KT164681.1 | *B.lucorum* |
|  |  |  |  |  |  |  |  |  |  |  |  |  |
| Hlyabovo | 42.06055 | 26.26459 | 2013_Bt_0108 | 25.05.2013 | MT174685 | 1866 | 1866 | 100% | 0.0 | 99.71% | NC_045179.1 | *B.terrestris* |
| Hlyabovo | 42.06055 | 26.26459 | 2013_Bt_0109 | 25.05.2013 | MT174686 | 1334 | 1334 | 94% | 0.0 | 91.71% | AY181170.1 | *B.terrestris* |
| Hlyabovo | 42.06055 | 26.26459 | 2013_Bt_0111 | 25.05.2013 | MT174687 | 1868 | 1868 | 100% | 0.0 | 99.90% | NC_045179.1 | *B.terrestris* |
| Hlyabovo | 42.06055 | 26.26459 | 2013_Bt_0112 | 25.05.2013 | MT174688 | 1895 | 1895 | 100% | 0.0 | 100.00% | NC_045179.1 | *B.terrestris* |
| Hlyabovo | 42.06055 | 26.26459 | 2013_Bt_0113 | 25.05.2013 | MT174689 | 1868 | 1868 | 99% | 0.0 | 99.71% | NC_045179.1 | *B.terrestris* |
| Hlyabovo | 42.06055 | 26.26459 | 2013_Bt_0114 | 25.05.2013 | MT174690 | 1882 | 1882 | 100% | 0.0 | 99.71% | NC_045179.1 | *B.terrestris* |
| Hlyabovo | 42.06055 | 26.26459 | 2013_Bt_0115 | 25.05.2013 | MT174691 | 1888 | 1888 | 100% | 0.0 | 99.81% | NC_045179.1 | *B.terrestris* |
| Hlyabovo | 42.06055 | 26.26459 | 2013_Bt_0116 | 25.05.2013 | MT174692 | 1892 | 1892 | 99% | 0.0 | 99.81% | NC_045179.1 | *B.terrestris* |
| Hlyabovo | 42.06055 | 26.26459 | 2013_Bt_0117 | 25.05.2013 | MT174693 | 1853 | 1853 | 99% | 0.0 | 99.61% | NC_045179.1 | *B.terrestris* |
| Hlyabovo | 42.06055 | 26.26459 | 2013_Bt_0118 | 25.05.2013 | MT174694 | 1873 | 1873 | 100% | 0.0 | 99.80% | NC_045179.1 | *B.terrestris* |
| Hlyabovo | 42.06055 | 26.26459 | 2013_Bt_0119 | 25.05.2013 | MT174695 | 1812 | 1812 | 100% | 0.0 | 99.60% | NC_045179.1 | *B.terrestris* |
|  |  |  |  |  |  |  |  |  |  |  |  |  |
| Iesle | 47.31038 | 25.89774 | 2017_Bombus_028 | 06.05.2017 | MT174773 | 1855 | 1855 | 100% | 0.0 | 99.22% | KT164681.1 | *B.lucorum* |
| Iesle | 47.31038 | 25.89774 | 2017_Bombus_029 | 06.05.2017 | MT174774 | 1838 | 1838 | 100% | 0.0 | 99.90% | KT164681.1 | *B.lucorum* |
| Iesle | 47.31038 | 25.89774 | 2017_Bombus_030 | 06.05.2017 | MT174775 | 1858 | 1858 | 100% | 0.0 | 99.41% | KT164681.1 | *B.lucorum* |
| Iesle | 47.31038 | 25.89774 | 2017_Bombus_031 | 06.05.2017 | MT174776 | 1879 | 1879 | 100% | 0.0 | 99.52% | KT164681.1 | *B.lucorum* |
| Iesle | 47.31038 | 25.89774 | 2017_Bombus_032 | 06.05.2017 | MT174777 | 1857 | 1857 | 100% | 0.0 | 99.90% | KT164681.1 | *B.lucorum* |
| Iesle | 47.31038 | 25.89774 | 2017_Bombus_033 | 06.05.2017 | MT174778 | 1823 | 1823 | 100% | 0.0 | 99.90% | NC_045179.1 | *B.terrestris* |
| Iesle | 47.31038 | 25.89774 | 2017_Bombus_034 | 06.05.2017 | MT174779 | 1834 | 1834 | 100% | 0.0 | 99.50% | KT164681.1 | *B.lucorum* |
| Iesle | 47.31038 | 25.89774 | 2017_Bombus_035 | 06.05.2017 | MT174780 | 1829 | 1829 | 100% | 0.0 | 99.70% | KT164681.1 | *B.lucorum* |
| Iesle | 47.31038 | 25.89774 | 2017_Bombus_037 | 06.05.2017 | MT174781 | 1855 | 1855 | 100% | 0.0 | 99.80% | KT164681.1 | *B.lucorum* |
| Iesle | 47.31038 | 25.89774 | 2017_Bombus_038 | 06.05.2017 | MT174782 | 1844 | 1844 | 100% | 0.0 | 99.80% | KT164681.1 | *B.lucorum* |
| Iesle | 47.31038 | 25.89774 | 2017_Bombus_039 | 06.05.2017 | MT174783 | 1825 | 1825 | 100% | 0.0 | 99.40% | KT164681.1 | *B.lucorum* |
| Iesle | 47.31038 | 25.89774 | 2017_Bombus_040 | 06.05.2017 | MT174784 | 1797 | 1797 | 100% | 0.0 | 98.81% | KT164681.1 | *B.lucorum* |
| Iesle | 47.31038 | 25.89774 | 2017_Bombus_041 | 06.05.2017 | MT174785 | 1858 | 1858 | 100% | 0.0 | 99.80% | KT164681.1 | *B.lucorum* |
| Iod | 46.93652 | 25.00172 | 2017_Bombus_395 | 27.08.2017 | MT175130 | 1784 | 1784 | 100% | 0.0 | 99.49% | KT164681.1 | *B.lucorum* |
| Iod | 46.93652 | 25.00172 | 2017_Bombus_396 | 27.08.2017 | MT175131 | 1371 | 1371 | 100% | 0.0 | 99.87% | KT164681.1 | *B.lucorum* |
| Iod | 46.93652 | 25.00172 | 2017_Bombus_397 | 27.08.2017 | MT175132 | 1821 | 1821 | 99% | 0.0 | 99.21% | KT164681.1 | *B.lucorum* |
| Iod | 46.93652 | 25.00172 | 2017_Bombus_398 | 27.08.2017 | MT175133 | 1375 | 1375 | 100% | 0.0 | 99.87% | KT164681.1 | *B.lucorum* |
| Iod | 46.93652 | 25.00172 | 2017_Bombus_399 | 27.08.2017 | MT175134 | 1834 | 1834 | 99% | 0.0 | 99.70% | KT164681.1 | *B.lucorum* |
| Iod | 46.93652 | 25.00172 | 2017_Bombus_400 | 27.08.2017 | MT175135 | 1659 | 1659 | 100% | 0.0 | 98.22% | KT164681.1 | *B.lucorum* |
| Iod | 46.93652 | 25.00172 | 2017_Bombus_401 | 27.08.2017 | MT175136 | 1182 | 1182 | 100% | 0.0 | 99.69% | KT164681.1 | *B.lucorum* |
| Iod | 46.93652 | 25.00172 | 2017_Bombus_402 | 27.08.2017 | MT175137 | 1600 | 1600 | 100% | 0.0 | 97.96% | KT164681.1 | *B.lucorum* |
| Iod | 46.93652 | 25.00172 | 2017_Bombus_403 | 27.08.2017 | MT175138 | 1557 | 1557 | 100% | 0.0 | 97.60% | KT164681.1 | *B.lucorum* |
| Iod | 46.93652 | 25.00172 | 2017_Bombus_404 | 27.08.2017 | MT175139 | 1279 | 1279 | 100% | 0.0 | 98.37% | KT164681.1 | *B.lucorum* |
| Iod | 46.93652 | 25.00172 | 2017_Bombus_405 | 27.08.2017 | MT175140 | 1858 | 1858 | 99% | 0.0 | 100.00% | KT164681.1 | *B.lucorum* |
| Iod | 46.93652 | 25.00172 | 2017_Bombus_406 | 27.08.2017 | MT175141 | 1435 | 1435 | 99% | 0.0 | 98.53% | KT164681.1 | *B.lucorum* |
|  |  |  |  |  |  |  |  |  |  |  |  |  |
| Kamenitsa | 41.64449 | 23.17299 | 2017_Bombus_276 | 01.06.2017 | MT175015 | 1775 | 1775 | 100% | 0.0 | 99.69% | NC_045179.1 | *B.terrestris* |
| Kamenitsa | 41.64449 | 23.17299 | 2017_Bombus_277 | 01.06.2017 | MT175016 | 1836 | 1836 | 99% | 0.0 | 99.50% | NC_045179.1 | *B.terrestris* |
| Kamenitsa | 41.64449 | 23.17299 | 2017_Bombus_278 | 01.06.2017 | MT175017 | 1735 | 1735 | 99% | 0.0 | 99.58% | NC_045179.1 | *B.terrestris* |
| Kamenitsa | 41.64449 | 23.17299 | 2017_Bombus_279 | 01.06.2017 | MT175018 | 1683 | 1683 | 100% | 0.0 | 99.25% | NC_045179.1 | *B.terrestris* |
| Kamenitsa | 41.64449 | 23.17299 | 2017_Bombus_280 | 01.06.2017 | MT175019 | 1831 | 1831 | 99% | 0.0 | 98.56% | NC_045179.1 | *B.terrestris* |
| Kamenitsa | 41.64449 | 23.17299 | 2017_Bombus_282 | 01.06.2017 | MT175020 | 1807 | 1807 | 100% | 0.0 | 99.50% | NC_045179.1 | *B.terrestris* |
| Kamenitsa | 41.64449 | 23.17299 | 2017_Bombus_283 | 01.06.2017 | MT175021 | 1622 | 1622 | 100% | 0.0 | 99.89% | NC_045179.1 | *B.terrestris* |
| Kamenitsa | 41.64449 | 23.17299 | 2017_Bombus_284 | 01.06.2017 | MT175022 | 1832 | 1832 | 99% | 0.0 | 99.41% | NC_045179.1 | *B.terrestris* |
| Kamenitsa | 41.64449 | 23.17299 | 2017_Bombus_285 | 01.06.2017 | MT175023 | 1823 | 1823 | 100% | 0.0 | 98.93% | NC_045179.1 | *B.terrestris* |
| Kamenitsa | 41.64449 | 23.17299 | 2017_Bombus_286 | 01.06.2017 | MT175024 | 1773 | 1773 | 100% | 0.0 | 99.79% | NC_045179.1 | *B.terrestris* |
| Kamenitsa | 41.64449 | 23.17299 | 2017_Bombus_287 | 01.06.2017 | MT175025 | 1768 | 1768 | 100% | 0.0 | 99.79% | NC_045179.1 | *B.terrestris* |
| Kamenitsa | 41.64449 | 23.17299 | 2017_Bombus_288 | 01.06.2017 | MT175026 | 1784 | 1784 | 99% | 0.0 | 99.79% | NC_045179.1 | *B.terrestris* |
|  |  |  |  |  |  |  |  |  |  |  |  |  |
| Koevtsi | 43.15832 | 25.09082 | 2013_Bt_0068 | 24.05.2013 | MT174655 | 1866 | 1866 | 100% | 0.0 | 99.61% | NC_045179.1 | *B.terrestris* |
| Koevtsi | 43.15832 | 25.09082 | 2013_Bt_0069 | 24.05.2013 | MT174656 | 1875 | 1875 | 100% | 0.0 | 99.90% | NC_045179.1 | *B.terrestris* |
| Koevtsi | 43.15832 | 25.09082 | 2013_Bt_0070 | 24.05.2013 | MT174657 | 1847 | 1847 | 100% | 0.0 | 99.61% | NC_045179.1 | *B.terrestris* |
| Koevtsi | 43.15832 | 25.09082 | 2013_Bt_0071 | 24.05.2013 | MT174658 | 1775 | 1775 | 99% | 0.0 | 99.90% | AY181170.1 | *B.terrestris* |
| Koevtsi | 43.15832 | 25.09082 | 2013_Bt_0073 | 24.05.2013 | MT174659 | 1834 | 1834 | 97% | 0.0 | 98.65% | NC_045179.1 | *B.terrestris* |
| Koevtsi | 43.15832 | 25.09082 | 2013_Bt_0074 | 24.05.2013 | MT174660 | 1864 | 1864 | 99% | 0.0 | 99.71% | NC_045179.1 | *B.terrestris* |
| Koevtsi | 43.15832 | 25.09082 | 2013_Bt_0075 | 24.05.2013 | MT174661 | 1838 | 1838 | 100% | 0.0 | 100.00% | NC_045179.1 | *B.terrestris* |
| Koevtsi | 43.15832 | 25.09082 | 2013_Bt_0076 | 24.05.2013 | MT174662 | 1853 | 1853 | 100% | 0.0 | 99.61% | NC_045179.1 | *B.terrestris* |
| Koevtsi | 43.15832 | 25.09082 | 2013_Bt_0077 | 24.05.2013 | MT174663 | 1857 | 1857 | 100% | 0.0 | 99.90% | NC_045179.1 | *B.terrestris* |
| Koevtsi | 43.15832 | 25.09082 | 2013_Bt_0078 | 24.05.2013 | MT174664 | 1860 | 1860 | 100% | 0.0 | 99.71% | NC_045179.1 | *B.terrestris* |
| Koevtsi | 43.15832 | 25.09082 | 2013_Bt_0079 | 24.05.2013 | MT174665 | 1844 | 1844 | 100% | 0.0 | 99.90% | NC_045179.1 | *B.terrestris* |
| Koevtsi | 43.15832 | 25.09082 | 2013_Bt_0081 | 24.05.2013 | MT174666 | 1873 | 1873 | 100% | 0.0 | 99.80% | NC_045179.1 | *B.terrestris* |
| Koevtsi | 43.15832 | 25.09082 | 2013_Bt_0082 | 24.05.2013 | MT174667 | 1685 | 1685 | 100% | 0.0 | 99.89% | AY181170.1 | *B.terrestris* |
| Koevtsi | 43.15832 | 25.09082 | 2013_Bt_0083 | 24.05.2013 | MT174668 | 1788 | 1788 | 100% | 0.0 | 99.90% | AY181170.1 | *B.terrestris* |
| Koevtsi | 43.15832 | 25.09082 | 2013_Bt_0084 | 24.05.2013 | MT174669 | 1700 | 1700 | 100% | 0.0 | 99.89% | NC_045179.1 | *B.terrestris* |
| Koevtsi | 43.15832 | 25.09082 | 2013_Bt_0085 | 24.05.2013 | MT174670 | 1858 | 1858 | 100% | 0.0 | 99.70% | NC_045179.1 | *B.terrestris* |
| Koevtsi | 43.15832 | 25.09082 | 2013_Bt_0086 | 24.05.2013 | MT174671 | 1866 | 1866 | 99% | 0.0 | 99.71% | NC_045179.1 | *B.terrestris* |
| Koevtsi | 43.15832 | 25.09082 | 2013_Bt_0087 | 24.05.2013 | MT174672 | 1775 | 1775 | 98% | 0.0 | 99.29% | NC_045179.1 | *B.terrestris* |
| Koevtsi | 43.15832 | 25.09082 | 2013_Bt_0088 | 24.05.2013 | MT174673 | 1749 | 1749 | 100% | 0.0 | 99.89% | NC_045179.1 | *B.terrestris* |
| Koevtsi | 43.15832 | 25.09082 | 2013_Bt_0089 | 24.05.2013 | MT174674 | 1823 | 1823 | 100% | 0.0 | 99.60% | NC_045179.1 | *B.terrestris* |
| Koevtsi | 43.15832 | 25.09082 | 2013_Bt_0090 | 24.05.2013 | MT174675 | 1552 | 1552 | 100% | 0.0 | 100.00% | AY181170.1 | *B.terrestris* |
|  |  |  |  |  |  |  |  |  |  |  |  |  |
| Levochevo | 41.60707 | 24.72302 | 2017_Bombus_175 | 31.05.2017 | MT174915 | 1888 | 1888 | 100% | 0.0 | 99.61% | KT164681.1 | *B.lucorum* |
| Levochevo | 41.60707 | 24.72302 | 2017_Bombus_176 | 31.05.2017 | MT174916 | 1869 | 1869 | 100% | 0.0 | 99.80% | KT164681.1 | *B.lucorum* |
| Levochevo | 41.60707 | 24.72302 | 2017_Bombus_177 | 31.05.2017 | MT174917 | 1849 | 1849 | 100% | 0.0 | 99.70% | KT164681.1 | *B.lucorum* |
| Levochevo | 41.60707 | 24.72302 | 2017_Bombus_178 | 31.05.2017 | MT174918 | 1888 | 1888 | 100% | 0.0 | 99.81% | NC_045179.1 | *B.terrestris* |
| Levochevo | 41.60707 | 24.72302 | 2017_Bombus_179 | 31.05.2017 | MT174919 | 1881 | 1881 | 100% | 0.0 | 99.90% | KT164681.1 | *B.lucorum* |
| Levochevo | 41.60707 | 24.72302 | 2017_Bombus_180 | 31.05.2017 | MT174920 | 1864 | 1864 | 100% | 0.0 | 99.80% | KT164681.1 | *B.lucorum* |
| Levochevo | 41.60707 | 24.72302 | 2017_Bombus_181 | 31.05.2017 | MT174921 | 1858 | 1858 | 100% | 0.0 | 99.90% | KT164681.1 | *B.lucorum* |
| Levochevo | 41.60707 | 24.72302 | 2017_Bombus_182 | 31.05.2017 | MT174922 | 1858 | 1858 | 99% | 0.0 | 99.70% | KT164681.1 | *B.lucorum* |
| Levochevo | 41.60707 | 24.72302 | 2017_Bombus_183 | 31.05.2017 | MT174923 | 1868 | 1868 | 100% | 0.0 | 99.90% | KT164681.1 | *B.lucorum* |
| Levochevo | 41.60707 | 24.72302 | 2017_Bombus_184 | 31.05.2017 | MT174924 | 1853 | 1853 | 99% | 0.0 | 99.80% | KT164681.1 | *B.lucorum* |
| Levochevo | 41.60707 | 24.72302 | 2017_Bombus_185 | 31.05.2017 | MT174925 | 1871 | 1871 | 99% | 0.0 | 99.90% | KT164681.1 | *B.lucorum* |
| Levochevo | 41.60707 | 24.72302 | 2017_Bombus_186 | 31.05.2017 | MT174926 | 1879 | 1879 | 99% | 0.0 | 99.90% | KT164681.1 | *B.lucorum* |
| Levochevo | 41.60707 | 24.72302 | 2017_Bombus_187 | 31.05.2017 | MT174927 | 1829 | 1829 | 99% | 0.0 | 99.50% | KT164681.1 | *B.lucorum* |
| Levochevo | 41.60707 | 24.72302 | 2017_Bombus_188 | 31.05.2017 | MT174928 | 1831 | 1831 | 100% | 0.0 | 99.70% | KT164681.1 | *B.lucorum* |
| Levochevo | 41.60707 | 24.72302 | 2017_Bombus_189 | 31.05.2017 | MT174929 | 1849 | 1849 | 100% | 0.0 | 99.61% | KT164681.1 | *B.lucorum* |
| Levochevo | 41.60707 | 24.72302 | 2017_Bombus_190 | 31.05.2017 | MT174930 | 1851 | 1851 | 99% | 0.0 | 99.90% | NC_045179.1 | *B.terrestris* |
| Levochevo | 41.60707 | 24.72302 | 2017_Bombus_191 | 31.05.2017 | MT174931 | 1821 | 1821 | 100% | 0.0 | 99.50% | KT164681.1 | *B.lucorum* |
| Levochevo | 41.60707 | 24.72302 | 2017_Bombus_192 | 31.05.2017 | MT174932 | 1844 | 1844 | 100% | 0.0 | 99.60% | KT164681.1 | *B.lucorum* |
| Levochevo | 41.60707 | 24.72302 | 2017_Bombus_193 | 31.05.2017 | MT174933 | 1849 | 1849 | 100% | 0.0 | 99.80% | KT164681.1 | *B.lucorum* |
| Levochevo | 41.60707 | 24.72302 | 2017_Bombus_194 | 31.05.2017 | MT174934 | 1849 | 1849 | 99% | 0.0 | 99.70% | KT164681.1 | *B.lucorum* |
| Levochevo | 41.60707 | 24.72302 | 2017_Bombus_195 | 31.05.2017 | MT174935 | 1871 | 1871 | 99% | 0.0 | 99.90% | KT164681.1 | *B.lucorum* |
| Levochevo | 41.60707 | 24.72302 | 2017_Bombus_196 | 31.05.2017 | MT174936 | 1851 | 1851 | 99% | 0.0 | 99.80% | KT164681.1 | *B.lucorum* |
| Levochevo | 41.60707 | 24.72302 | 2017_Bombus_197 | 31.05.2017 | MT174937 | 1840 | 1840 | 99% | 0.0 | 99.51% | KT164681.1 | *B.lucorum* |
| Levochevo | 41.60707 | 24.72302 | 2017_Bombus_198 | 31.05.2017 | MT174938 | 1879 | 1879 | 100% | 0.0 | 99.90% | KT164681.1 | *B.lucorum* |
| Levochevo | 41.60707 | 24.72302 | 2017_Bombus_199 | 31.05.2017 | MT174939 | 1869 | 1869 | 99% | 0.0 | 99.90% | KT164681.1 | *B.lucorum* |
| Levochevo | 41.60707 | 24.72302 | 2017_Bombus_200 | 31.05.2017 | MT174940 | 1873 | 1873 | 100% | 0.0 | 99.90% | KT164681.1 | *B.lucorum* |
| Levochevo | 41.60707 | 24.72302 | 2017_Bombus_201 | 31.05.2017 | MT174941 | 1851 | 1851 | 100% | 0.0 | 99.90% | KT164681.1 | *B.lucorum* |
| Levochevo | 41.60707 | 24.72302 | 2017_Bombus_202 | 31.05.2017 | MT174942 | 1862 | 1862 | 99% | 0.0 | 100.00% | KT164681.1 | *B.lucorum* |
| Levochevo | 41.60707 | 24.72302 | 2017_Bombus_203 | 31.05.2017 | MT174943 | 1864 | 1864 | 100% | 0.0 | 99.90% | KT164681.1 | *B.lucorum* |
| Levochevo | 41.60707 | 24.72302 | 2017_Bombus_204 | 31.05.2017 | MT174944 | 1849 | 1849 | 99% | 0.0 | 100.00% | KT164681.1 | *B.lucorum* |
|  |  |  |  |  |  |  |  |  |  |  |  |  |
| Mengishevo | 43.03566 | 26.64753 | 2013_Bt_0141 | 29.05.2013 | MT174709 | 1845 | 1845 | 100% | 0.0 | 99.51% | NC_045179.1 | *B.terrestris* |
| Mengishevo | 43.03566 | 26.64753 | 2013_Bt_0142 | 29.05.2013 | MT174710 | 1857 | 1857 | 99% | 0.0 | 99.80% | NC_045179.1 | *B.terrestris* |
| Mengishevo | 43.03566 | 26.64753 | 2013_Bt_0143 | 29.05.2013 | MT174711 | 1845 | 1845 | 100% | 0.0 | 99.90% | AY181170.1 | *B.terrestris* |
| Mengishevo | 43.03566 | 26.64753 | 2013_Bt_0144 | 29.05.2013 | MT174712 | 1862 | 1862 | 100% | 0.0 | 99.71% | NC_045179.1 | *B.terrestris* |
| Mengishevo | 43.03566 | 26.64753 | 2013_Bt_0145 | 29.05.2013 | MT174713 | 1853 | 1853 | 99% | 0.0 | 99.80% | NC_045179.1 | *B.terrestris* |
| Mengishevo | 43.03566 | 26.64753 | 2013_Bt_0146 | 29.05.2013 | MT174714 | 1866 | 1866 | 100% | 0.0 | 99.80% | NC_045179.1 | *B.terrestris* |
| Mengishevo | 43.03566 | 26.64753 | 2013_Bt_0147 | 29.05.2013 | MT174715 | 1866 | 1866 | 100% | 0.0 | 99.80% | NC_045179.1 | *B.terrestris* |
| Mengishevo | 43.03566 | 26.64753 | 2013_Bt_0148 | 29.05.2013 | MT174716 | 1869 | 1869 | 100% | 0.0 | 99.90% | NC_045179.1 | *B.terrestris* |
| Mengishevo | 43.03566 | 26.64753 | 2013_Bt_0149 | 29.05.2013 | MT174717 | 1875 | 1875 | 100% | 0.0 | 99.90% | NC_045179.1 | *B.terrestris* |
| Mengishevo | 43.03566 | 26.64753 | 2013_Bt_0150 | 29.05.2013 | MT174718 | 1853 | 1853 | 100% | 0.0 | 99.61% | NC_045179.1 | *B.terrestris* |
| Mengishevo | 43.03566 | 26.64753 | 2013_Bt_0151 | 29.05.2013 | MT174719 | 1796 | 1796 | 100% | 0.0 | 99.90% | NC_045179.1 | *B.terrestris* |
| Mengishevo | 43.03566 | 26.64753 | 2013_Bt_0152 | 29.05.2013 | MT174720 | 1853 | 1853 | 99% | 0.0 | 99.41% | NC_045179.1 | *B.terrestris* |
| Mengishevo | 43.03566 | 26.64753 | 2013_Bt_0153 | 29.05.2013 | MT174721 | 1873 | 1873 | 100% | 0.0 | 99.71% | NC_045179.1 | *B.terrestris* |
|  |  |  |  |  |  |  |  |  |  |  |  |  |
| Ojdula | 45.98988 | 26.29976 | 2013_Bt_0265 | 22.07.2013 | MT175190 | 1496 | 1496 | 100% | 0.0 | 98.25% | KT164681.1 | *B.lucorum* |
| Ojdula | 45.98988 | 26.29976 | 2013_Bt_0266 | 22.07.2013 | MT175191 | 1334 | 1334 | 100% | 0.0 | 99.32% | KT164681.1 | *B.lucorum* |
| Ojdula | 45.98988 | 26.29976 | 2013_Bt_0267 | 22.07.2013 | MT175192 | 1515 | 1515 | 100% | 0.0 | 98.49% | NC_045179.1 | *B.terrestris* |
| Ojdula | 45.98988 | 26.29976 | 2013_Bt_0268 | 22.07.2013 | MT175193 | 1378 | 1378 | 100% | 0.0 | 99.34% | KT164681.1 | *B.lucorum* |
| Ojdula | 45.98988 | 26.29976 | 2013_Bt_0269 | 22.07.2013 | MT175194 | 1443 | 1443 | 99% | 0.0 | 96.96% | KT164681.1 | *B.lucorum* |
| Ojdula | 45.98988 | 26.29976 | 2013_Bt_0270 | 22.07.2013 | MT175195 | 1712 | 1712 | 99% | 0.0 | 99.36% | KT164681.1 | *B.lucorum* |
| Ojdula | 45.98988 | 26.29976 | 2013_Bt_0272 | 22.07.2013 | MT175196 | 1493 | 1493 | 99% | 0.0 | 98.36% | KT164681.1 | *B.lucorum* |
| Ojdula | 45.98988 | 26.29976 | 2013_Bt_0273 | 22.07.2013 | MT175197 | 1482 | 1482 | 99% | 0.0 | 99.03% | KT164681.1 | *B.lucorum* |
| Ojdula | 45.98988 | 26.29976 | 2013_Bt_0274 | 22.07.2013 | MT175198 | 1688 | 1688 | 100% | 0.0 | 97.96% | KT164681.1 | *B.lucorum* |
| Ojdula | 45.98988 | 26.29976 | 2013_Bt_0275 | 22.07.2013 | MT175199 | 902 | 902 | 100% | 0.0 | 99.20% | KT164681.1 | *B.lucorum* |
| Ojdula | 45.98988 | 26.29976 | 2013_Bt_0276 | 22.07.2013 | MT175200 | 1349 | 1349 | 100% | 0.0 | 99.20% | KT164681.1 | *B.lucorum* |
| Ojdula | 45.98988 | 26.29976 | 2013_Bt_0278 | 22.07.2013 | MT175201 | 1480 | 1480 | 99% | 0.0 | 98.92% | KT164681.1 | *B.lucorum* |
| Ojdula | 45.98988 | 26.29976 | 2013_Bt_0279 | 22.07.2013 | MT175202 | 1751 | 1751 | 99% | 0.0 | 98.59% | KT164681.1 | *B.lucorum* |
| Ojdula | 45.98988 | 26.29976 | 2013_Bt_0280 | 22.07.2013 | MT175203 | 1461 | 1461 | 100% | 0.0 | 98.55% | KT164681.1 | *B.lucorum* |
| Ojdula | 45.98988 | 26.29976 | 2013_Bt_0281 | 22.07.2013 | MT175204 | 1347 | 1347 | 100% | 0.0 | 99.46% | KT164681.1 | *B.lucorum* |
| Ojdula | 45.98988 | 26.29976 | 2013_Bt_0283 | 22.07.2013 | MT175205 | 1507 | 1507 | 100% | 0.0 | 98.48% | KT164681.1 | *B.lucorum* |
|  |  |  |  |  |  |  |  |  |  |  |  |  |
| Orsova | 44.75420 | 22.3948 | 2013_Bt_0175 | 04.06.2013 | MT174735 | 1783 | 1783 | 100% | 0.0 | 100.00% | AY181170.1 | *B.terrestris* |
| Orsova | 44.75420 | 22.3948 | 2013_Bt_0176 | 04.06.2013 | MT174736 | 1770 | 1770 | 100% | 0.0 | 99.90% | NC_045179.1 | *B.terrestris* |
| Orsova | 44.75420 | 22.3948 | 2013_Bt_0177 | 04.06.2013 | MT174737 | 1871 | 1871 | 99% | 0.0 | 99.90% | NC_045179.1 | *B.terrestris* |
| Orsova | 44.75420 | 22.3948 | 2013_Bt_0178 | 04.06.2013 | MT174738 | 1881 | 1881 | 99% | 0.0 | 99.80% | NC_045179.1 | *B.terrestris* |
| Orsova | 44.75420 | 22.3948 | 2013_Bt_0179 | 04.06.2013 | MT174739 | 1881 | 1881 | 99% | 0.0 | 99.90% | NC_045179.1 | *B.terrestris* |
| Orsova | 44.75420 | 22.3948 | 2013_Bt_0181 | 04.06.2013 | MT174740 | 1868 | 1868 | 100% | 0.0 | 99.61% | NC_045179.1 | *B.terrestris* |
| Orsova | 44.75420 | 22.3948 | 2013_Bt_0182 | 04.06.2013 | MT174741 | 1792 | 1792 | 100% | 0.0 | 99.90% | NC_045179.1 | *B.terrestris* |
| Orsova | 44.75420 | 22.3948 | 2013_Bt_0183 | 04.06.2013 | MT174742 | 1869 | 1869 | 100% | 0.0 | 99.90% | NC_045179.1 | *B.terrestris* |
| Orsova | 44.75420 | 22.3948 | 2013_Bt_0184 | 04.06.2013 | MT174743 | 1810 | 1810 | 100% | 0.0 | 99.80% | NC_045179.1 | *B.terrestris* |
| Orsova | 44.75420 | 22.3948 | 2013_Bt_0185 | 04.06.2013 | MT174744 | 1840 | 1840 | 99% | 0.0 | 99.90% | AY181170.1 | *B.terrestris* |
| Orsova | 44.75420 | 22.3948 | 2013_Bt_0186 | 04.06.2013 | MT174745 | 1855 | 1855 | 99% | 0.0 | 99.90% | NC_045179.1 | *B.terrestris* |
| Orsova | 44.75420 | 22.3948 | 2013_Bt_0187 | 04.06.2013 | MT174746 | 1842 | 1842 | 100% | 0.0 | 99.41% | NC_045179.1 | *B.terrestris* |
| Orsova | 44.75420 | 22.3948 | 2013_Bt_0188 | 04.06.2013 | MT174747 | 1853 | 1853 | 99% | 0.0 | 99.70% | NC_045179.1 | *B.terrestris* |
| Orsova | 44.75420 | 22.3948 | 2013_Bt_0189 | 04.06.2013 | MT174748 | 1868 | 1868 | 100% | 0.0 | 99.80% | KT164681.1 | *B.lucorum* |
| Orsova | 44.75420 | 22.3948 | 2013_Bt_0190 | 04.06.2013 | MT174749 | 1881 | 1881 | 99% | 0.0 | 99.80% | KT164681.1 | *B.lucorum* |
| Orsova | 44.75420 | 22.3948 | 2013_Bt_0191 | 04.06.2013 | MT174750 | 1879 | 1879 | 100% | 0.0 | 99.90% | NC_045179.1 | *B.terrestris* |
|  |  |  |  |  |  |  |  |  |  |  |  |  |
| Pastra | 42.12283 | 23.20023 | 2013_Bt_0004 | 18.05.2013 | MT174625 | 1784 | 1784 | 100% | 0.0 | 99.69% | NC_045179.1 | *B.terrestris* |
| Pastra | 42.12283 | 23.20023 | 2013_Bt_0005 | 18.05.2013 | MT174626 | 1770 | 1770 | 99% | 0.0 | 99.69% | NC_045179.1 | *B.terrestris* |
| Pastra | 42.12283 | 23.20023 | 2013_Bt_0011 | 18.05.2013 | MT174627 | 1550 | 1550 | 99% | 0.0 | 96.36% | NC_045179.1 | *B.terrestris* |
|  |  |  |  |  |  |  |  |  |  |  |  |  |
| Pietroasa | 46.58998 | 22.58807 | Bt_2014_153 | 02.07.2014 | MT175302 | 1827 | 1827 | 99% | 0.0 | 99.80% | AY181170.1 | *B.terrestris* |
| Pietroasa | 46.58998 | 22.58807 | Bt_2014_154 | 02.07.2014 | MT175303 | 1834 | 1834 | 99% | 0.0 | 99.70% | NC_045179.1 | *B.terrestris* |
| Pietroasa | 46.58998 | 22.58807 | Bt_2014_156 | 02.07.2014 | MT175304 | 1821 | 1821 | 99% | 0.0 | 98.93% | NC_045179. | *B.terrestris* |
| Pietroasa | 46.58998 | 22.58807 | Bt_2014_157 | 02.07.2014 | MT175305 | 1655 | 1655 | 99% | 0.0 | 96.81% | NC_045179.1 | *B.terrestris* |
| Pietroasa | 46.58998 | 22.58807 | Bt_2014_158 | 02.07.2014 | MT175306 | 1851 | 1851 | 99% | 0.0 | 99.70% | NC_045179.1 | *B.terrestris* |
| Pietroasa | 46.58998 | 22.58807 | Bt_2014_159 | 02.07.2014 | MT175307 | 1609 | 1609 | 99% | 0.0 | 96.92% | AY181170.1 | *B.terrestris* |
| Pietroasa | 46.58998 | 22.58807 | Bt_2014_161 | 02.07.2014 | MT175308 | 1812 | 1812 | 100% | 0.0 | 99.21% | NC_045179.1 | *B.terrestris* |
| Pietroasa | 46.58998 | 22.58807 | Bt_2014_163 | 02.07.2014 | MT175309 | 1825 | 1825 | 100% | 0.0 | 99.60% | NC_045179.1 | *B.terrestris* |
| Pietroasa | 46.58998 | 22.58807 | Bt_2014_164 | 02.07.2014 | MT175310 | 1759 | 1759 | 100% | 0.0 | 98.04% | NC_045179.1 | *B.terrestris* |
| Pietroasa | 46.58998 | 22.58807 | Bt_2014_166 | 02.07.2014 | MT175311 | 1845 | 1845 | 100% | 0.0 | 99.41% | NC_045179.1 | *B.terrestris* |
| Pietroasa | 46.58998 | 22.58807 | Bt_2014_167 | 02.07.2014 | MT175312 | 1834 | 1834 | 100% | 0.0 | 99.41% | NC_045179.1 | *B.terrestris* |
| Pietroasa | 46.58998 | 22.58807 | Bt_2014_170 | 02.07.2014 | MT175313 | 1801 | 1801 | 100% | 0.0 | 98.63% | NC_045179.1 | *B.terrestris* |
|  |  |  |  |  |  |  |  |  |  |  |  |  |
| Pirin | 41.52480 | 23.5879 | 2017_Bombus_240 | 01.06.2017 | MT174980 | 1842 | 1842 | 100% | 0.0 | 99.90% | KT164681.1 | *B.lucorum* |
| Pirin | 41.52480 | 23.5879 | 2017_Bombus_241 | 01.06.2017 | MT174981 | 833 | 833 | 99% | 0.0 | 99.78% | KT164681.1 | *B.lucorum* |
| Pirin | 41.52480 | 23.5879 | 2017_Bombus_242 | 01.06.2017 | MT174982 | 1845 | 1845 | 100% | 0.0 | 99.90% | KT164681.1 | *B.lucorum* |
| Pirin | 41.52480 | 23.5879 | 2017_Bombus_243 | 01.06.2017 | MT174983 | 1801 | 1801 | 100% | 0.0 | 99.80% | NC_045179.1 | *B.terrestris* |
| Pirin | 41.52480 | 23.5879 | 2017_Bombus_244 | 01.06.2017 | MT174984 | 1330 | 1330 | 98% | 0.0 | 98.05% | AY530010.1 | *B.lucorum* |
| Pirin | 41.52480 | 23.5879 | 2017_Bombus_245 | 01.06.2017 | MT174985 | 1821 | 1821 | 99% | 0.0 | 99.40% | NC_045179.1 | *B.terrestris* |
| Pirin | 41.52480 | 23.5879 | 2017_Bombus_246 | 01.06.2017 | MT174986 | 1831 | 1831 | 100% | 0.0 | 99.80% | KT164681.1 | *B.lucorum* |
| Pirin | 41.52480 | 23.5879 | 2017_Bombus_247 | 01.06.2017 | MT174987 | 1701 | 1701 | 100% | 0.0 | 99.05% | KT164681.1 | *B.lucorum* |
| Pirin | 41.52480 | 23.5879 | 2017_Bombus_248 | 01.06.2017 | MT174988 | 1701 | 1701 | 99% | 0.0 | 100.00% | KT164681.1 | *B.lucorum* |
| Pirin | 41.52480 | 23.5879 | 2017_Bombus_249 | 01.06.2017 | MT174989 | 1413 | 1413 | 100% | 0.0 | 99.74% | AY181170.1 | *B.terrestris* |
| Pirin | 41.52480 | 23.5879 | 2017_Bombus_250 | 01.06.2017 | MT174990 | 1810 | 1810 | 100% | 0.0 | 99.50% | KT164681.1 | *B.lucorum* |
| Pirin | 41.52480 | 23.5879 | 2017_Bombus_251 | 01.06.2017 | MT174991 | 603 | 603 | 90% | 2e-168 | 99.10% | KT164681.1 | *B.lucorum* |
| Pirin | 41.52480 | 23.5879 | 2017_Bombus_252 | 01.06.2017 | MT174992 | 1506 | 1506 | 100% | 0.0 | 99.88% | KT164681.1 | *B.lucorum* |
| Pirin | 41.52480 | 23.5879 | 2017_Bombus_253 | 01.06.2017 | MT174993 | 1356 | 1356 | 99% | 0.0 | 97.74% | AY181170.1 | *B.terrestris* |
| Pirin | 41.52480 | 23.5879 | 2017_Bombus_254 | 01.06.2017 | MT174994 | 1821 | 1821 | 100% | 0.0 | 100.00% | AY181170.1 | *B.terrestris* |
|  |  |  |  |  |  |  |  |  |  |  |  |  |
| Poienita | 45.82299 | 24.57591 | 2013_Bt_0312 | 27.07.2013 | MT175206 | 1489 | 1489 | 100% | 0.0 | 98.46% | NC_045179.1 | *B.terrestris* |
| Poienita | 45.82299 | 24.57591 | 2013_Bt_0313 | 27.07.2013 | MT175207 | 1419 | 1419 | 99% | 0.0 | 99.74% | NC_045179.1 | *B.terrestris* |
| Poienita | 45.82299 | 24.57591 | 2013_Bt_0314 | 27.07.2013 | MT175208 | 1391 | 1391 | 100% | 0.0 | 98.73% | AY181170.1 | *B.terrestris* |
| Poienita | 45.82299 | 24.57591 | 2013_Bt_0315 | 27.07.2013 | MT175209 | 1391 | 1391 | 100% | 0.0 | 98.73% | AY181170.1 | *B.terrestris* |
| Poienita | 45.82299 | 24.57591 | 2013_Bt_0316 | 27.07.2013 | MT175210 | 1655 | 1655 | 100% | 0.0 | 99.45% | NC_045179.1 | *B.terrestris* |
| Poienita | 45.82299 | 24.57591 | 2013_Bt_0317 | 27.07.2013 | MT175211 | 1635 | 1635 | 100% | 0.0 | 98.60% | AY181170.1 | *B.terrestris* |
| Poienita | 45.82299 | 24.57591 | 2013_Bt_0318 | 27.07.2013 | MT175212 | 1580 | 1580 | 100% | 0.0 | 99.43% | AY181170.1 | *B.terrestris* |
| Poienita | 45.82299 | 24.57591 | 2013_Bt_0319 | 27.07.2013 | MT175213 | 1037 | 1037 | 100% | 0.0 | 99.30% | NC_045179.1 | *B.terrestris* |
| Poienita | 45.82299 | 24.57591 | 2013_Bt_0320 | 27.07.2013 | MT175214 | 1642 | 1642 | 100% | 0.0 | 99.56% | AY181170.1 | *B.terrestris* |
| Poienita | 45.82299 | 24.57591 | 2013_Bt_0321 | 27.07.2013 | MT175215 | 1629 | 1629 | 100% | 0.0 | 99.33% | AY181170.1 | *B.terrestris* |
| Poienita | 45.82299 | 24.57591 | 2013_Bt_0322 | 27.07.2013 | MT175216 | 990 | 990 | 100% | 0.0 | 98.74% | AY181170.1 | *B.terrestris* |
| Poienita | 45.82299 | 24.57591 | 2013_Bt_0323 | 27.07.2013 | MT175217 | 1489 | 1489 | 99% | 0.0 | 99.39% | NC_045179.1 | *B.terrestris* |
| Poienita | 45.82299 | 24.57591 | 2013_Bt_0324 | 27.07.2013 | MT175218 | 1173 | 1173 | 100% | 0.0 | 99.38% | NC_045179.1 | *B.terrestris* |
| Poienita | 45.82299 | 24.57591 | 2013_Bt_0325 | 27.07.2013 | MT175219 | 1629 | 1629 | 100% | 0.0 | 98.49% | NC_045179.1 | *B.terrestris* |
| Poienita | 45.82299 | 24.57591 | 2013_Bt_0326 | 27.07.2013 | MT175220 | 1351 | 1351 | 99% | 0.0 | 99.20% | AY181170.1 | *B.terrestris* |
| Poienita | 45.82299 | 24.57591 | 2013_Bt_0327 | 27.07.2013 | MT175221 | 1716 | 1716 | 99% | 0.0 | 99.58% | AY181170.1 | *B.terrestris* |
| Poienita | 45.82299 | 24.57591 | 2013_Bt_0328 | 27.07.2013 | MT175222 | 1371 | 1371 | 100% | 0.0 | 98.71% | AY181170.1 | *B.terrestris* |
| Poienita | 45.82299 | 24.57591 | 2013_Bt_0329 | 27.07.2013 | MT175223 | 1550 | 1550 | 98% | 0.0 | 98.85% | NC_045179.1 | *B.terrestris* |
| Poienita | 45.82299 | 24.57591 | 2013_Bt_0330 | 27.07.2013 | MT175224 | 1260 | 1260 | 100% | 0.0 | 98.47% | AY181170.1 | *B.terrestris* |
| Poienita | 45.82299 | 24.57591 | 2013_Bt_0331 | 27.07.2013 | MT175225 | 1515 | 1515 | 99% | 0.0 | 99.05% | KT164681.1 | *B.lucorum* |
|  |  |  |  |  |  |  |  |  |  |  |  |  |
| Polovragi | 45.21492 | 23.77486 | 2013_Bt_0332 | 30.07.2013 | MT175226 | 920 | 920 | 100% | 0.0 | 99.22% | KT164681.1 | *B.lucorum* |
| Polovragi | 45.21492 | 23.77486 | 2013_Bt_0333 | 30.07.2013 | MT175227 | 1271 | 1271 | 100% | 0.0 | 97.97% | KT164681.1 | *B.lucorum* |
| Polovragi | 45.21492 | 23.77486 | 2013_Bt_0334 | 30.07.2013 | MT175228 | 1081 | 1081 | 100% | 0.0 | 99.33% | KT164681.1 | *B.lucorum* |
| Polovragi | 45.21492 | 23.77486 | 2013_Bt_0335 | 30.07.2013 | MT175229 | 1332 | 1332 | 99% | 0.0 | 99.19% | KT164681.1 | *B.lucorum* |
| Polovragi | 45.21492 | 23.77486 | 2013_Bt_0336 | 30.07.2013 | MT175230 | 924 | 924 | 100% | 0.0 | 99.60% | KT164681.1 | *B.lucorum* |
| Polovragi | 45.21492 | 23.77486 | 2013_Bt_0337 | 30.07.2013 | MT175231 | 1260 | 1260 | 100% | 0.0 | 98.74% | KT164681.1 | *B.lucorum* |
| Polovragi | 45.21492 | 23.77486 | 2013_Bt_0338 | 30.07.2013 | MT175232 | 1701 | 1701 | 99% | 0.0 | 98.55% | KT164681.1 | *B.lucorum* |
| Polovragi | 45.21492 | 23.77486 | 2013_Bt_0339 | 30.07.2013 | MT175233 | 1506 | 1506 | 99% | 0.0 | 98.15% | KT164681.1 | *B.lucorum* |
| Polovragi | 45.21492 | 23.77486 | 2013_Bt_0341 | 30.07.2013 | MT175234 | 1062 | 1062 | 99% | 0.0 | 99.32% | KT164681.1 | *B.lucorum* |
| Polovragi | 45.21492 | 23.77486 | 2013_Bt_0342 | 30.07.2013 | MT175235 | 1286 | 1286 | 100% | 0.0 | 99.03% | KT164681.1 | *B.lucorum* |
| Polovragi | 45.21492 | 23.77486 | 2013_Bt_0343 | 30.07.2013 | MT175236 | 1596 | 1596 | 100% | 0.0 | 98.26% | KT164681.1 | *B.lucorum* |
| Polovragi | 45.21492 | 23.77486 | 2013_Bt_0344 | 30.07.2013 | MT175237 | 455 | 455 | 100% | 5e-124 | 98.83% | KT164681.1 | *B.lucorum* |
|  |  |  |  |  |  |  |  |  |  |  |  |  |
| Razdelna | 42.18144 | 25.90854 | 2013_Bt_0099 | 25.05.2013 | MT174681 | 1784 | 1784 | 100% | 0.0 | 100.00% | NC_045179.1 | *B.terrestris* |
| Razdelna | 42.18144 | 25.90854 | 2013_Bt_0102 | 25.05.2013 | MT174682 | 1783 | 1783 | 100% | 0.0 | 100.00% | AY181170.1 | *B.terrestris* |
| Razdelna | 42.18144 | 25.90854 | 2013_Bt_0103 | 25.05.2013 | MT174683 | 741 | 741 | 100% | 0.0 | 87.98% | AY181170.1 | *B.terrestris* |
| Razdelna | 42.18144 | 25.90854 | 2013_Bt_0105 | 25.05.2013 | MT174684 | 1635 | 1635 | 100% | 0.0 | 100.00% | AY181170.1 | *B.terrestris* |
|  |  |  |  |  |  |  |  |  |  |  |  |  |
| Rilski Manastir | 42.09243 | 23.38633 | 2013_Bt_0016 | 18.05.2013 | MT174628 | 1825 | 1825 | 100% | 0.0 | 99.80% | KT164681.1 | *B.lucorum* |
| Rilski Manastir | 42.09243 | 23.38633 | 2013_Bt_0021 | 18.05.2013 | MT174629 | 1773 | 1773 | 100% | 0.0 | 100.00% | KT164681.1 | *B.lucorum* |
| Rilski Manastir | 42.09243 | 23.38633 | 2013_Bt_0022 | 18.05.2013 | MT174630 | 1755 | 1755 | 100% | 0.0 | 100.00% | KT164681.1 | *B.lucorum* |
|  |  |  |  |  |  |  |  |  |  |  |  |  |
| Rish | 42.97442 | 26.90731 | 2017_Bombus_128 | 29.05.2017 | MT174868 | 1327 | 1327 | 100% | 0.0 | 99.19% | AY181170.1 | *B.terrestris* |
| Rish | 42.97442 | 26.90731 | 2017_Bombus_129 | 29.05.2017 | MT174869 | 1794 | 1794 | 100% | 0.0 | 98.91% | NC_045179.1 | *B.terrestris* |
| Rish | 42.97442 | 26.90731 | 2017_Bombus_130 | 29.05.2017 | MT174870 | 1879 | 1879 | 99% | 0.0 | 100.00% | NC_045179.1 | *B.terrestris* |
| Rish | 42.97442 | 26.90731 | 2017_Bombus_131 | 29.05.2017 | MT174871 | 1650 | 1650 | 99% | 0.0 | 99.89% | AY181170.1 | *B.terrestris* |
| Rish | 42.97442 | 26.90731 | 2017_Bombus_132 | 29.05.2017 | MT174872 | 1596 | 1596 | 100% | 0.0 | 99.43% | NC_045179.1 | *B.terrestris* |
| Rish | 42.97442 | 26.90731 | 2017_Bombus_133 | 29.05.2017 | MT174873 | 1315 | 1315 | 100% | 0.0 | 99.32% | AY181170.1 | *B.terrestris* |
| Rish | 42.97442 | 26.90731 | 2017_Bombus_134 | 29.05.2017 | MT174874 | 1799 | 1799 | 100% | 0.0 | 99.69% | NC_045179.1 | *B.terrestris* |
| Rish | 42.97442 | 26.90731 | 2017_Bombus_135 | 29.05.2017 | MT174875 | 1759 | 1759 | 99% | 0.0 | 98.69% | NC_045179.1 | *B.terrestris* |
| Rish | 42.97442 | 26.90731 | 2017_Bombus_136 | 29.05.2017 | MT174876 | 1714 | 1714 | 99% | 0.0 | 98.27% | AY181170.1 | *B.terrestris* |
| Rish | 42.97442 | 26.90731 | 2017_Bombus_137 | 29.05.2017 | MT174877 | 1836 | 1836 | 100% | 0.0 | 99.31% | NC_045179.1 | *B.terrestris* |
| Rish | 42.97442 | 26.90731 | 2017_Bombus_138 | 29.05.2017 | MT174878 | 1736 | 1736 | 100% | 0.0 | 99.07% | AY181170.1 | *B.terrestris* |
| Rish | 42.97442 | 26.90731 | 2017_Bombus_139 | 29.05.2017 | MT174879 | 1816 | 1816 | 100% | 0.0 | 99.70% | NC_045179.1 | *B.terrestris* |
| Rish | 42.97442 | 26.90731 | 2017_Bombus_140 | 29.05.2017 | MT174880 | 1879 | 1879 | 100% | 0.0 | 100.00% | NC_045179.1 | *B.terrestris* |
| Rish | 42.97442 | 26.90731 | 2017_Bombus_141 | 29.05.2017 | MT174881 | 1868 | 1868 | 99% | 0.0 | 99.71% | NC_045179.1 | *B.terrestris* |
| Rish | 42.97442 | 26.90731 | 2017_Bombus_142 | 29.05.2017 | MT174882 | 1700 | 1700 | 100% | 0.0 | 98.07% | NC_045179.1 | *B.terrestris* |
| Rish | 42.97442 | 26.90731 | 2017_Bombus_143 | 29.05.2017 | MT174883 | 1687 | 1687 | 98% | 0.0 | 99.25% | NC_045179.1 | *B.terrestris* |
| Rish | 42.97442 | 26.90731 | 2017_Bombus_144 | 29.05.2017 | MT174884 | 1836 | 1836 | 100% | 0.0 | 99.80% | NC_045179.1 | *B.terrestris* |
| Rish | 42.97442 | 26.90731 | 2017_Bombus_145 | 29.05.2017 | MT174885 | 1855 | 1855 | 100% | 0.0 | 99.70% | NC_045179.1 | *B.terrestris* |
| Rish | 42.97442 | 26.90731 | 2017_Bombus_146 | 29.05.2017 | MT174886 | 1844 | 1844 | 100% | 0.0 | 99.70% | NC_045179.1 | *B.terrestris* |
| Rish | 42.97442 | 26.90731 | 2017_Bombus_147 | 29.05.2017 | MT174887 | 1773 | 1773 | 99% | 0.0 | 98.70% | NC_045179.1 | *B.terrestris* |
| Rish | 42.97442 | 26.90731 | 2017_Bombus_148 | 29.05.2017 | MT174888 | 1725 | 1725 | 100% | 0.0 | 98.19% | AY181170.1 | *B.terrestris* |
| Rish | 42.97442 | 26.90731 | 2017_Bombus_149 | 29.05.2017 | MT174889 | 1877 | 1877 | 100% | 0.0 | 99.71% | NC_045179.1 | *B.terrestris* |
| Rish | 42.97442 | 26.90731 | 2017_Bombus_150 | 29.05.2017 | MT174890 | 1832 | 1832 | 100% | 0.0 | 99.60% | NC_045179.1 | *B.terrestris* |
| Rish | 42.97442 | 26.90731 | 2017_Bombus_151 | 29.05.2017 | MT174891 | 1844 | 1844 | 100% | 0.0 | 99.80% | NC_045179.1 | *B.terrestris* |
| Rish | 42.97442 | 26.90731 | 2017_Bombus_152 | 29.05.2017 | MT174892 | 1836 | 1836 | 100% | 0.0 | 99.80% | NC_045179.1 | *B.terrestris* |
| Rish | 42.97442 | 26.90731 | 2017_Bombus_153 | 29.05.2017 | MT174893 | 1858 | 1858 | 99% | 0.0 | 99.51% | NC_045179.1 | *B.terrestris* |
| Rish | 42.97442 | 26.90731 | 2017_Bombus_154 | 29.05.2017 | MT174894 | 1849 | 1849 | 99% | 0.0 | 99.90% | NC_045179.1 | *B.terrestris* |
| Rish | 42.97442 | 26.90731 | 2017_Bombus_155 | 29.05.2017 | MT174895 | 1877 | 1877 | 100% | 0.0 | 99.71% | NC_045179.1 | *B.terrestris* |
| Rish | 42.97442 | 26.90731 | 2017_Bombus_156 | 29.05.2017 | MT174896 | 1864 | 1864 | 100% | 0.0 | 99.80% | NC_045179.1 | *B.terrestris* |
| Rish | 42.97442 | 26.90731 | 2017_Bombus_157 | 29.05.2017 | MT174897 | 1851 | 1851 | 100% | 0.0 | 99.90% | NC_045179.1 | *B.terrestris* |
| Rish | 42.97442 | 26.90731 | 2017_Bombus_158 | 29.05.2017 | MT174898 | 1855 | 1855 | 99% | 0.0 | 99.70% | NC_045179.1 | *B.terrestris* |
| Rish | 42.97442 | 26.90731 | 2017_Bombus_159 | 29.05.2017 | MT174899 | 1857 | 1857 | 100% | 0.0 | 99.90% | NC_045179.1 | *B.terrestris* |
|  |  |  |  |  |  |  |  |  |  |  |  |  |
| Sinemorets | 42.04499 | 27.95808 | 2017_Bombus_436 | 03.09.2017 | MT175142 | 1740 | 1740 | 99% | 0.0 | 99.89% | NC_045179.1 | *B.terrestris* |
| Sinemorets | 42.04499 | 27.95808 | 2017_Bombus_437 | 03.09.2017 | MT175143 | 1663 | 1663 | 100% | 0.0 | 98.72% | NC_045179.1 | *B.terrestris* |
| Sinemorets | 42.04499 | 27.95808 | 2017_Bombus_438 | 03.09.2017 | MT175144 | 1554 | 1554 | 100% | 0.0 | 99.76% | NC_045179.1 | *B.terrestris* |
| Sinemorets | 42.04499 | 27.95808 | 2017_Bombus_439 | 03.09.2017 | MT175145 | 1474 | 1474 | 100% | 0.0 | 99.03% | AY181170.1 | *B.terrestris* |
| Sinemorets | 42.04499 | 27.95808 | 2017_Bombus_440 | 03.09.2017 | MT175146 | 1507 | 1507 | 100% | 0.0 | 99.88% | NC_045179.1 | *B.terrestris* |
| Sinemorets | 42.04499 | 27.95808 | 2017_Bombus_441 | 03.09.2017 | MT175147 | 915 | 915 | 100% | 0.0 | 100.00% | NC_045179.1 | *B.terrestris* |
| Sinemorets | 42.04499 | 27.95808 | 2017_Bombus_442 | 03.09.2017 | MT175148 | 1491 | 1491 | 100% | 0.0 | 99.04% | NC_045179.1 | *B.terrestris* |
| Sinemorets | 42.04499 | 27.95808 | 2017_Bombus_443 | 03.09.2017 | MT175149 | 1652 | 1652 | 100% | 0.0 | 99.45% | NC_045179.1 | *B.terrestris* |
| Sinemorets | 42.04499 | 27.95808 | 2017_Bombus_444 | 03.09.2017 | MT175150 | 1544 | 1544 | 100% | 0.0 | 98.96% | NC_045179.1 | *B.terrestris* |
| Sinemorets | 42.04499 | 27.95808 | 2017_Bombus_445 | 03.09.2017 | MT175151 | 1393 | 1393 | 100% | 0.0 | 100.00% | KT164681.1 | *B.lucorum* |
| Sinemorets | 42.04499 | 27.95808 | 2017_Bombus_446 | 03.09.2017 | MT175152 | 833 | 833 | 100% | 0.0 | 100.00% | EF362742.1 | *B.terrestris* |
| Sinemorets | 42.04499 | 27.95808 | 2017_Bombus_447 | 03.09.2017 | MT175153 | 1360 | 1360 | 100% | 0.0 | 99.60% | NC_045179.1 | *B.terrestris* |
|  |  |  |  |  |  |  |  |  |  |  |  |  |
| Stambolovo | 41.78435 | 25.63166 | 2017_Bombus_160 | 30.05.2017 | MT174900 | 1840 | 1840 | 99% | 0.0 | 99.60% | NC_045179.1 | *B.terrestris* |
| Stambolovo | 41.78435 | 25.63166 | 2017_Bombus_161 | 30.05.2017 | MT174901 | 1803 | 1803 | 100% | 0.0 | 99.20% | NC_045179.1 | *B.terrestris* |
| Stambolovo | 41.78435 | 25.63166 | 2017_Bombus_162 | 30.05.2017 | MT174902 | 1842 | 1842 | 100% | 0.0 | 99.80% | NC_045179.1 | *B.terrestris* |
| Stambolovo | 41.78435 | 25.63166 | 2017_Bombus_163 | 30.05.2017 | MT174903 | 1851 | 1851 | 100% | 0.0 | 99.90% | NC_045179.1 | *B.terrestris* |
| Stambolovo | 41.78435 | 25.63166 | 2017_Bombus_164 | 30.05.2017 | MT174904 | 1829 | 1829 | 100% | 0.0 | 99.90% | NC_045179.1 | *B.terrestris* |
| Stambolovo | 41.78435 | 25.63166 | 2017_Bombus_165 | 30.05.2017 | MT174905 | 1860 | 1860 | 100% | 0.0 | 99.90% | NC_045179.1 | *B.terrestris* |
| Stambolovo | 41.78435 | 25.63166 | 2017_Bombus_166 | 30.05.2017 | MT174906 | 1866 | 1866 | 100% | 0.0 | 100.00% | NC_045179.1 | *B.terrestris* |
| Stambolovo | 41.78435 | 25.63166 | 2017_Bombus_167 | 30.05.2017 | MT174907 | 1845 | 1845 | 99% | 0.0 | 99.80% | NC_045179.1 | *B.terrestris* |
| Stambolovo | 41.78435 | 25.63166 | 2017_Bombus_168 | 30.05.2017 | MT174908 | 1860 | 1860 | 100% | 0.0 | 99.90% | NC_045179.1 | *B.terrestris* |
| Stambolovo | 41.78435 | 25.63166 | 2017_Bombus_169 | 30.05.2017 | MT174909 | 1847 | 1847 | 100% | 0.0 | 99.90% | NC_045179.1 | *B.terrestris* |
| Stambolovo | 41.78435 | 25.63166 | 2017_Bombus_170 | 30.05.2017 | MT174910 | 1857 | 1857 | 99% | 0.0 | 99.61% | NC_045179.1 | *B.terrestris* |
| Stambolovo | 41.78435 | 25.63166 | 2017_Bombus_171 | 30.05.2017 | MT174911 | 911 | 911 | 100% | 0.0 | 99.80% | KP025802.1 | *B.terrestris* |
| Stambolovo | 41.78435 | 25.63166 | 2017_Bombus_172 | 30.05.2017 | MT174912 | 1829 | 1829 | 99% | 0.0 | 99.70% | NC_045179.1 | *B.terrestris* |
| Stambolovo | 41.78435 | 25.63166 | 2017_Bombus_173 | 30.05.2017 | MT174913 | 1871 | 1871 | 100% | 0.0 | 99.90% | NC_045179.1 | *B.terrestris* |
| Stambolovo | 41.78435 | 25.63166 | 2017_Bombus_174 | 30.05.2017 | MT174914 | 1890 | 1890 | 99% | 0.0 | 99.81% | NC_045179.1 | *B.terrestris* |
|  |  |  |  |  |  |  |  |  |  |  |  |  |
| Strumeshnitsa | 41.39833 | 23.06046 | 2017_Bombus_255 | 01.06.2017 | MT174995 | 1742 | 1742 | 100% | 0.0 | 100.00% | NC_045179.1 | *B.terrestris* |
| Strumeshnitsa | 41.39833 | 23.06046 | 2017_Bombus_256 | 01.06.2017 | MT174996 | 1733 | 1733 | 100% | 0.0 | 99.58% | NC_045179.1 | *B.terrestris* |
| Strumeshnitsa | 41.39833 | 23.06046 | 2017_Bombus_257 | 01.06.2017 | MT174997 | 1393 | 1393 | 100% | 0.0 | 99.87% | NC_045179.1 | *B.terrestris* |
| Strumeshnitsa | 41.39833 | 23.06046 | 2017_Bombus_258 | 01.06.2017 | MT174998 | 1712 | 1712 | 99% | 0.0 | 98.37% | NC_045179.1 | *B.terrestris* |
| Strumeshnitsa | 41.39833 | 23.06046 | 2017_Bombus_259 | 01.06.2017 | MT174999 | 1729 | 1729 | 99% | 0.0 | 99.27% | AY181170.1 | *B.terrestris* |
| Strumeshnitsa | 41.39833 | 23.06046 | 2017_Bombus_260 | 01.06.2017 | MT175000 | 1528 | 1528 | 100% | 0.0 | 99.41% | AY181170.1 | *B.terrestris* |
| Strumeshnitsa | 41.39833 | 23.06046 | 2017_Bombus_261 | 01.06.2017 | MT175001 | 1546 | 1546 | 100% | 0.0 | 99.30% | AY181170.1 | *B.terrestris* |
| Strumeshnitsa | 41.39833 | 23.06046 | 2017_Bombus_262 | 01.06.2017 | MT175002 | 1360 | 1360 | 99% | 0.0 | 99.34% | NC_045179.1 | *B.terrestris* |
| Strumeshnitsa | 41.39833 | 23.06046 | 2017_Bombus_263 | 01.06.2017 | MT175003 | 1448 | 1448 | 100% | 0.0 | 98.54% | NC_045179.1 | *B.terrestris* |
| Strumeshnitsa | 41.39833 | 23.06046 | 2017_Bombus_265 | 01.06.2017 | MT175004 | 1648 | 1648 | 100% | 0.0 | 99.02% | NC_045179.1 | *B.terrestris* |
| Strumeshnitsa | 41.39833 | 23.06046 | 2017_Bombus_266 | 01.06.2017 | MT175005 | 1742 | 1742 | 100% | 0.0 | 98.29% | NC_045179.1 | *B.terrestris* |
| Strumeshnitsa | 41.39833 | 23.06046 | 2017_Bombus_267 | 01.06.2017 | MT175006 | 1810 | 1810 | 99% | 0.0 | 99.20% | NC_045179.1 | *B.terrestris* |
| Strumeshnitsa | 41.39833 | 23.06046 | 2017_Bombus_268 | 01.06.2017 | MT175007 | 1755 | 1755 | 99% | 0.0 | 99.08% | NC_045179.1 | *B.terrestris* |
| Strumeshnitsa | 41.39833 | 23.06046 | 2017_Bombus_269 | 01.06.2017 | MT175008 | 1797 | 1797 | 100% | 0.0 | 99.69% | NC_045179.1 | *B.terrestris* |
| Strumeshnitsa | 41.39833 | 23.06046 | 2017_Bombus_270 | 01.06.2017 | MT175009 | 1879 | 1879 | 99% | 0.0 | 99.80% | NC_045179.1 | *B.terrestris* |
| Strumeshnitsa | 41.39833 | 23.06046 | 2017_Bombus_271 | 01.06.2017 | MT175010 | 1836 | 1836 | 100% | 0.0 | 99.70% | NC_045179.1 | *B.terrestris* |
| Strumeshnitsa | 41.39833 | 23.06046 | 2017_Bombus_272 | 01.06.2017 | MT175011 | 1779 | 1779 | 99% | 0.0 | 99.59% | NC_045179.1 | *B.terrestris* |
| Strumeshnitsa | 41.39833 | 23.06046 | 2017_Bombus_273 | 01.06.2017 | MT175012 | 1820 | 1820 | 100% | 0.0 | 99.50% | NC_045179.1 | *B.terrestris* |
| Strumeshnitsa | 41.39833 | 23.06046 | 2017_Bombus_274 | 01.06.2017 | MT175013 | 1812 | 1812 | 100% | 0.0 | 99.11% | NC_045179.1 | *B.terrestris* |
| Strumeshnitsa | 41.39833 | 23.06046 | 2017_Bombus_275 | 01.06.2017 | MT175014 | 1790 | 1790 | 99% | 0.0 | 99.30% | NC_045179.1 | *B.terrestris* |
|  |  |  |  |  |  |  |  |  |  |  |  |  |
| Topa Mica | 46.92851 | 23.40238 | Bt_2014_104 | 14.06.2014 | MT175260 | 1594 | 1594 | 100% | 0.0 | 96.46% | NC_045179.1 | *B.terrestris* |
| Topa Mica | 46.92851 | 23.40238 | Bt_2014_105 | 14.06.2014 | MT175261 | 1827 | 1827 | 95% | 0.0 | 99.12% | NC_045179.1 | *B.terrestris* |
| Topa Mica | 46.92851 | 23.40238 | Bt_2014_106 | 14.06.2014 | MT175262 | 1832 | 1832 | 99% | 0.0 | 99.31% | NC_045179.1 | *B.terrestris* |
| Topa Mica | 46.92851 | 23.40238 | Bt_2014_107 | 14.06.2014 | MT175263 | 1836 | 1836 | 100% | 0.0 | 99.80% | NC_045179.1 | *B.terrestris* |
| Topa Mica | 46.92851 | 23.40238 | Bt_2014_108 | 14.06.2014 | MT175264 | 1823 | 1823 | 100% | 0.0 | 99.60% | NC_045179.1 | *B.terrestris* |
| Topa Mica | 46.92851 | 23.40238 | Bt_2014_109 | 14.06.2014 | MT175265 | 1805 | 1805 | 99% | 0.0 | 98.35% | NC_045179.1 | *B.terrestris* |
| Topa Mica | 46.92851 | 23.40238 | Bt_2014_110 | 14.06.2014 | MT175266 | 1825 | 1825 | 99% | 0.0 | 99.40% | NC_045179.1 | *B.terrestris* |
| Topa Mica | 46.92851 | 23.40238 | Bt_2014_111 | 14.06.2014 | MT175267 | 1838 | 1838 | 100% | 0.0 | 98.93% | NC_045179.1 | *B.terrestris* |
| Topa Mica | 46.92851 | 23.40238 | Bt_2014_112 | 14.06.2014 | MT175268 | 1832 | 1832 | 100% | 0.0 | 99.50% | NC_045179.1 | *B.terrestris* |
| Topa Mica | 46.92851 | 23.40238 | Bt_2014_113 | 14.06.2014 | MT175269 | 1808 | 1808 | 99% | 0.0 | 99.30% | NC_045179.1 | *B.terrestris* |
| Topa Mica | 46.92851 | 23.40238 | Bt_2014_114 | 14.06.2014 | MT175270 | 1807 | 1807 | 100% | 0.0 | 98.73% | NC_045179.1 | *B.terrestris* |
| Topa Mica | 46.92851 | 23.40238 | Bt_2014_115 | 14.06.2014 | MT175271 | 1810 | 1810 | 99% | 0.0 | 99.50% | NC_045179.1 | *B.terrestris* |
| Topa Mica | 46.92851 | 23.40238 | Bt_2014_116 | 14.06.2014 | MT175272 | 1803 | 1803 | 99% | 0.0 | 99.40% | NC_045179.1 | *B.terrestris* |
| Topa Mica | 46.92851 | 23.40238 | Bt_2014_117 | 14.06.2014 | MT175273 | 1814 | 1814 | 97% | 0.0 | 99.40% | NC_045179.1 | *B.terrestris* |
| Topa Mica | 46.92851 | 23.40238 | Bt_2014_118 | 14.06.2014 | MT175274 | 1788 | 1788 | 100% | 0.0 | 99.10% | NC_045179.1 | *B.terrestris* |
| Topa Mica | 46.92851 | 23.40238 | Bt_2014_119 | 14.06.2014 | MT175275 | 1816 | 1816 | 99% | 0.0 | 99.50% | NC_045179.1 | *B.terrestris* |
| Topa Mica | 46.92851 | 23.40238 | Bt_2014_120 | 14.06.2014 | MT175276 | 1842 | 1842 | 100% | 0.0 | 98.75% | NC_045179.1 | *B.terrestris* |
| Topa Mica | 46.92851 | 23.40238 | Bt_2014_121 | 14.06.2014 | MT175277 | 1821 | 1821 | 99% | 0.0 | 99.70% | NC_045179.1 | *B.terrestris* |
| Topa Mica | 46.92851 | 23.40238 | Bt_2014_122 | 14.06.2014 | MT175278 | 1805 | 1805 | 99% | 0.0 | 99.20% | NC_045179.1 | *B.terrestris* |
| Topa Mica | 46.92851 | 23.40238 | Bt_2014_123 | 14.06.2014 | MT175279 | 1834 | 1834 | 100% | 0.0 | 99.70% | NC_045179.1 | *B.terrestris* |
| Topa Mica | 46.92851 | 23.40238 | Bt_2014_124 | 14.06.2014 | MT175280 | 1842 | 1842 | 95% | 0.0 | 99.03% | NC_045179.1 | *B.terrestris* |
|  |  |  |  |  |  |  |  |  |  |  |  |  |
| Toplita | 46.98115 | 25.40812 | Bt_2014_079 | 09.06.2014 | MT175258 | 1162 | 1162 | 100% | 0.0 | 99.69% | KT164681.1 | *B.lucorum* |
| Toplita | 46.98115 | 25.40812 | Bt_2014_082 | 09.06.2014 | MT175259 | 1657 | 1657 | 100% | 0.0 | 99.78% | KT164681.1 | *B.lucorum* |
|  |  |  |  |  |  |  |  |  |  |  |  |  |
| Valea Hotarului | 47.93870 | 23.83761 | Bt_2014_192 | 07.07.2014 | MT175314 | 1849 | 1849 | 100% | 0.0 | 99.03% | NC_045179. | *B.terrestris* |
| Valea Hotarului | 47.93870 | 23.83761 | Bt_2014_193 | 07.07.2014 | MT175315 | 1834 | 1834 | 100% | 0.0 | 99.60% | NC_045179.1 | *B.terrestris* |
| Valea Hotarului | 47.93870 | 23.83761 | Bt_2014_194 | 07.07.2014 | MT175316 | 1853 | 1853 | 100% | 0.0 | 99.80% | NC_045179. | *B.terrestris* |
| Valea Hotarului | 47.93870 | 23.83761 | Bt_2014_195 | 07.07.2014 | MT175317 | 1832 | 1832 | 99% | 0.0 | 99.60% | NC_045179.1 | *B.terrestris* |
| Valea Hotarului | 47.93870 | 23.83761 | Bt_2014_196 | 07.07.2014 | MT175318 | 1832 | 1832 | 100% | 0.0 | 98.93% | NC_045179.1 | *B.terrestris* |
| Valea Hotarului | 47.93870 | 23.83761 | Bt_2014_197 | 07.07.2014 | MT175319 | 1845 | 1845 | 100% | 0.0 | 99.70% | NC_045179.1 | *B.terrestris* |
| Valea Hotarului | 47.93870 | 23.83761 | Bt_2014_198 | 07.07.2014 | MT175320 | 1844 | 1844 | 99% | 0.0 | 99.70% | NC_045179.1 | *B.terrestris* |
| Valea Hotarului | 47.93870 | 23.83761 | Bt_2014_199 | 07.07.2014 | MT175321 | 1864 | 1864 | 100% | 0.0 | 99.80% | NC_045179.1 | *B.terrestris* |
| Valea Hotarului | 47.93870 | 23.83761 | Bt_2014_200 | 07.07.2014 | MT175322 | 1844 | 1844 | 100% | 0.0 | 99.60% | NC_045179.1 | *B.terrestris* |
| Valea Hotarului | 47.93870 | 23.83761 | Bt_2014_201 | 07.07.2014 | MT175323 | 1847 | 1847 | 100% | 0.0 | 99.90% | NC_045179.1 | *B.terrestris* |
| Valea Hotarului | 47.93870 | 23.83761 | Bt_2014_202 | 07.07.2014 | MT175324 | 1855 | 1855 | 99% | 0.0 | 99.70% | NC_045179.1 | *B.terrestris* |
| Valea Hotarului | 47.93870 | 23.83761 | Bt_2014_203 | 07.07.2014 | MT175325 | 1868 | 1868 | 100% | 0.0 | 100.00% | KT164681.1 | *B.lucorum* |
| Valea Hotarului | 47.93870 | 23.83761 | Bt_2014_204 | 07.07.2014 | MT175326 | 1842 | 1842 | 100% | 0.0 | 99.51% | NC_045179.1 | *B.terrestris* |
| Valea Hotarului | 47.93870 | 23.83761 | Bt_2014_205 | 07.07.2014 | MT175327 | 1821 | 1821 | 100% | 0.0 | 99.80% | NC_045179.1 | *B.terrestris* |
| Valea Hotarului | 47.93870 | 23.83761 | Bt_2014_206 | 07.07.2014 | MT175328 | 1855 | 1855 | 100% | 0.0 | 99.41% | NC_045179.1 | *B.terrestris* |
| Valea Hotarului | 47.93870 | 23.83761 | Bt_2014_207 | 07.07.2014 | MT175329 | 1849 | 1849 | 100% | 0.0 | 99.61% | NC_045179.1 | *B.terrestris* |
| Valea Hotarului | 47.93870 | 23.83761 | Bt_2014_208 | 07.07.2014 | MT175330 | 1853 | 1853 | 99% | 0.0 | 99.90% | NC_045179.1 | *B.terrestris* |
| Valea Hotarului | 47.93870 | 23.83761 | Bt_2014_209 | 07.07.2014 | MT175331 | 1823 | 1823 | 100% | 0.0 | 99.40% | NC_045179.1 | *B.terrestris* |
| Valea Hotarului | 47.93870 | 23.83761 | Bt_2014_210 | 07.07.2014 | MT175332 | 1853 | 1853 | 98% | 0.0 | 99.61% | NC_045179.1 | *B.terrestris* |
| Valea Hotarului | 47.93870 | 23.83761 | Bt_2014_211 | 07.07.2014 | MT175333 | 1832 | 1832 | 99% | 0.0 | 99.90% | NC_045179.1 | *B.terrestris* |
| Valea Hotarului | 47.93870 | 23.83761 | Bt_2014_212 | 07.07.2014 | MT175334 | 1840 | 1840 | 97% | 0.0 | 99.31% | NC_045179.1 | *B.terrestris* |
|  |  |  |  |  |  |  |  |  |  |  |  |  |
| Valea Pӑdurii | 46.62236 | 24.02727 | 2017_Bombus_383 | 27.08.2017 | MT175118 | 1074 | 1074 | 100% | 0.0 | 100.00% | EF362742.1 | *B.terrestris* |
| Valea Pӑdurii | 46.62236 | 24.02727 | 2017_Bombus_384 | 27.08.2017 | MT175119 | 1772 | 1772 | 100% | 0.0 | 99.59% | AY181170.1 | *B.terrestris* |
| Valea Pӑdurii | 46.62236 | 24.02727 | 2017_Bombus_385 | 27.08.2017 | MT175120 | 1127 | 1127 | 100% | 0.0 | 99.84% | NC_045179.1 | *B.terrestris* |
| Valea Pӑdurii | 46.62236 | 24.02727 | 2017_Bombus_386 | 27.08.2017 | MT175121 | 1424 | 1424 | 100% | 0.0 | 99.12% | NC_045179.1 | *B.terrestris* |
| Valea Pӑdurii | 46.62236 | 24.02727 | 2017_Bombus_387 | 27.08.2017 | MT175122 | 1452 | 1452 | 100% | 0.0 | 95.58% | NC_045179.1 | *B.terrestris* |
| Valea Pӑdurii | 46.62236 | 24.02727 | 2017_Bombus_388 | 27.08.2017 | MT175123 | 1504 | 1504 | 100% | 0.0 | 97.42% | NC_045179.1 | *B.terrestris* |
| Valea Pӑdurii | 46.62236 | 24.02727 | 2017_Bombus_389 | 27.08.2017 | MT175124 | 1526 | 1526 | 100% | 0.0 | 98.40% | NC_045179.1 | *B.terrestris* |
| Valea Pӑdurii | 46.62236 | 24.02727 | 2017_Bombus_390 | 27.08.2017 | MT175125 | 1513 | 1513 | 99% | 0.0 | 97.43% | NC_045179.1 | *B.terrestris* |
| Valea Pӑdurii | 46.62236 | 24.02727 | 2017_Bombus_391 | 27.08.2017 | MT175126 | 1714 | 1714 | 100% | 0.0 | 99.47% | NC_045179.1 | *B.terrestris* |
| Valea Pӑdurii | 46.62236 | 24.02727 | 2017_Bombus_392 | 27.08.2017 | MT175127 | 1836 | 1836 | 100% | 0.0 | 99.70% | NC_045179.1 | *B.terrestris* |
| Valea Pӑdurii | 46.62236 | 24.02727 | 2017_Bombus_393 | 27.08.2017 | MT175128 | 1742 | 1742 | 99% | 0.0 | 99.28% | NC_045179.1 | *B.terrestris* |
| Valea Pӑdurii | 46.62236 | 24.02727 | 2017_Bombus_394 | 27.08.2017 | MT175129 | 1548 | 1548 | 100% | 0.0 | 96.44% | NC_045179.1 | *B.terrestris* |
|  |  |  |  |  |  |  |  |  |  |  |  |  |
| Zdravets | 42.94361 | 24.15964 | 2013_Bt_0051 | 22.05.2013 | MT174640 | 1777 | 1777 | 99% | 0.0 | 99.79% | NC_045179.1 | *B.terrestris* |
| Zdravets | 42.94361 | 24.15964 | 2013_Bt_0052 | 22.05.2013 | MT174641 | 1349 | 1349 | 99% | 0.0 | 98.20% | NC_045179.1 | *B.terrestris* |
| Zdravets | 42.94361 | 24.15964 | 2013_Bt_0053 | 22.05.2013 | MT174642 | 1773 | 1773 | 100% | 0.0 | 99.69% | NC_045179.1 | *B.terrestris* |
| Zdravets | 42.94361 | 24.15964 | 2013_Bt_0054 | 22.05.2013 | MT174643 | 1772 | 1772 | 100% | 0.0 | 99.69% | KT164681.1 | *B.lucorum* |
| Zdravets | 42.94361 | 24.15964 | 2013_Bt_0055 | 22.05.2013 | MT174644 | 1877 | 1877 | 99% | 0.0 | 99.80% | NC_045179.1 | *B.terrestris* |
| Zdravets | 42.94361 | 24.15964 | 2013_Bt_0056 | 22.05.2013 | MT174645 | 1744 | 1744 | 100% | 0.0 | 99.48% | KT164681.1 | *B.lucorum* |
| Zdravets | 42.94361 | 24.15964 | 2013_Bt_0057 | 22.05.2013 | MT174646 | 1369 | 1369 | 99% | 0.0 | 98.46% | KT164681.1 | *B.lucorum* |
| Zdravets | 42.94361 | 24.15964 | 2013_Bt_0058 | 22.05.2013 | MT174647 | 1805 | 1805 | 100% | 0.0 | 99.90% | KT164681.1 | *B.lucorum* |
| Zdravets | 42.94361 | 24.15964 | 2013_Bt_0061 | 22.05.2013 | MT174648 | 1498 | 1498 | 100% | 0.0 | 99.64% | KT164681.1 | *B.lucorum* |
| Zdravets | 42.94361 | 24.15964 | 2013_Bt_0063 | 22.05.2013 | MT174649 | 1646 | 1646 | 99% | 0.0 | 99.45% | NC_045179.1 | *B.terrestris* |
| Zdravets | 42.94361 | 24.15964 | 2013_Bt_0064 | 22.05.2013 | MT174650 | 1519 | 1519 | 99% | 0.0 | 99.88% | KT164681.1 | *B.lucorum* |
| Zdravets | 42.94361 | 24.15964 | 2013_Bt_0065a | 22.05.2013 | MT174651 | 1679 | 1679 | 100% | 0.0 | 100.00% | KT164681.1 | *B.lucorum* |
| Zdravets | 42.94361 | 24.15964 | 2013_Bt_0065b | 22.05.2013 | MT174652 | 1892 | 1892 | 99% | 0.0 | 99.90% | KT164681.1 | *B.lucorum* |
| Zdravets | 42.94361 | 24.15964 | 2013_Bt_0066 | 22.05.2013 | MT174653 | 1881 | 1881 | 100% | 0.0 | 99.80% | NC_045179.1 | *B.terrestris* |
| Zdravets | 42.94361 | 24.15964 | 2013_Bt_0067 | 22.05.2013 | MT174654 | 1875 | 1875 | 100% | 0.0 | 99.80% | KT164681.1 | *B.lucorum* |
|  |  |  |  |  |  |  |  |  |  |  |  |  |
| Zlatitza | 42.70908 | 24.12053 | 2013_Bt_0041 | 21.05.2013 | MT174631 | 1321 | 1321 | 99% | 0.0 | 99.59% | NC_045179.1 | *B.terrestris* |
| Zlatitza | 42.70908 | 24.12053 | 2013_Bt_0042 | 21.05.2013 | MT174632 | 1628 | 1628 | 98% | 0.0 | 99.55% | KT164681.1 | *B.lucorum* |
| Zlatitza | 42.70908 | 24.12053 | 2013_Bt_0043 | 21.05.2013 | MT174633 | 1480 | 1480 | 100% | 0.0 | 99.75% | KT164681.1 | *B.lucorum* |
| Zlatitza | 42.70908 | 24.12053 | 2013_Bt_0044 | 21.05.2013 | MT174634 | 1875 | 1875 | 100% | 0.0 | 99.90% | NC_045179.1 | *B.terrestris* |
| Zlatitza | 42.70908 | 24.12053 | 2013_Bt_0045 | 21.05.2013 | MT174635 | 1869 | 1869 | 99% | 0.0 | 99.61% | NC_045179.1 | *B.terrestris* |
| Zlatitza | 42.70908 | 24.12053 | 2013_Bt_0046 | 21.05.2013 | MT174636 | 1784 | 1784 | 100% | 0.0 | 99.59% | KT164681.1 | *B.lucorum* |
| Zlatitza | 42.70908 | 24.12053 | 2013_Bt_0047 | 21.05.2013 | MT174637 | 1781 | 1781 | 100% | 0.0 | 99.69% | KT164681.1 | *B.lucorum* |
| Zlatitza | 42.70908 | 24.12053 | 2013_Bt_0048 | 21.05.2013 | MT174638 | 1447 | 1447 | 99% | 0.0 | 98.54% | KT164681.1 | *B.lucorum* |
| Zlatitza | 42.70908 | 24.12053 | 2013_Bt_0049 | 21.05.2013 | MT174639 | 1522 | 1522 | 99% | 0.0 | 95.76% | KT164681.1 | *B.lucorum* |
